# Chitosan and its Derivatives for Ocular Delivery Formulations: Recent Advances and Developments

**DOI:** 10.3390/polym12071519

**Published:** 2020-07-08

**Authors:** Alexandra Zamboulis, Stavroula Nanaki, Georgia Michailidou, Ioanna Koumentakou, Maria Lazaridou, Nina Maria Ainali, Eleftheria Xanthopoulou, Dimitrios N. Bikiaris

**Affiliations:** Laboratory of Polymer Chemistry & Technology, Department of Chemistry, Aristotle University of Thessaloniki, 54124 Thessaloniki, Greece; sgnanaki@chem.auth.gr (S.N.); michailidougeorgia18@gmail.com (G.M.); iwanna.koumentakou@gmail.com (I.K.); marlazach@chem.auth.gr (M.L.); ainali.nina@gmail.com (N.M.A.); elefthxanthopoulou@gmail.com (E.X.)

**Keywords:** chitosan, derivatives, ocular drug delivery, ophthalmic applications, mucoadhesion, antibacterial, nanoparticles, hydrogels, coatings

## Abstract

Chitosan (CS) is a hemi-synthetic cationic linear polysaccharide produced by the deacetylation of chitin. CS is non-toxic, highly biocompatible, and biodegradable, and it has a low immunogenicity. Additionally, CS has inherent antibacterial properties and a mucoadhesive character and can disrupt epithelial tight junctions, thus acting as a permeability enhancer. As such, CS and its derivatives are well-suited for the challenging field of ocular drug delivery. In the present review article, we will discuss the properties of CS that contribute to its successful application in ocular delivery before reviewing the latest advances in the use of CS for the development of novel ophthalmic delivery systems. Colloidal nanocarriers (nanoparticles, micelles, liposomes) will be presented, followed by CS gels and lenses and ocular inserts. Finally, instances of CS coatings, aiming at conferring mucoadhesiveness to other matrixes, will be presented.

## 1. Introduction

Ocular diseases affect a growing number of people across the globe. Some pathological ophthalmic conditions, such as glaucoma, diabetic retinopathy, or age-related macular degeneration, cause severe visual impairment that can ultimately lead to blindness. In spite of their relative accessibility, or rather because of it, eyes are well-protected organs and successful ocular drug delivery is one of the important challenges the pharmaceutical industry has to face.

**Figure 1:**
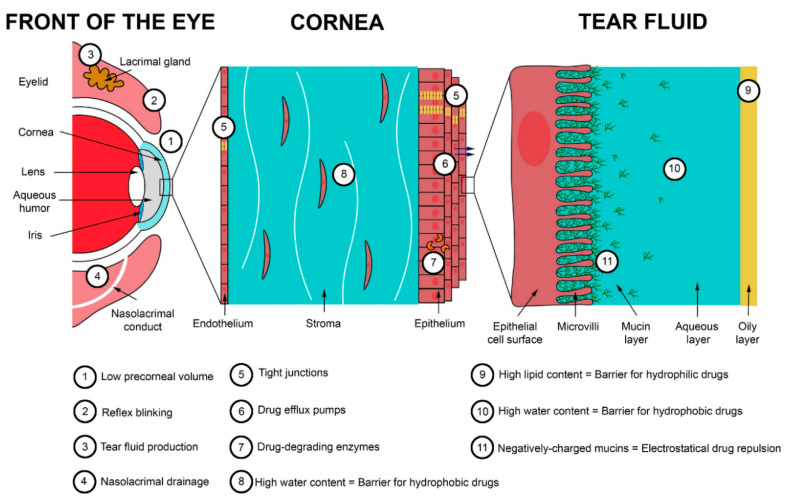
Main barriers to ocular delivery. Reprinted from ref. [2]. Copyright 2020 with permission from Elsevier.

Ocular tissues are protected from foreign substances by a series of static and dynamic barriers and protective mechanisms, as illustrated in Figure 1 [1]. Tear turnover, reflex blinking, and nasolacrimal drainage drain agents away from the eye surface. The corneal epithelium and conjunctiva cover and protect the ocular surface. Additionally, the blood–ocular barriers (blood–aqueous and blood–retina) limit the access of compounds from systemic circulation. This defensive system is further assisted by enzymes and other barriers (corneal stroma, sclera, etc.).

**Figure 2:**
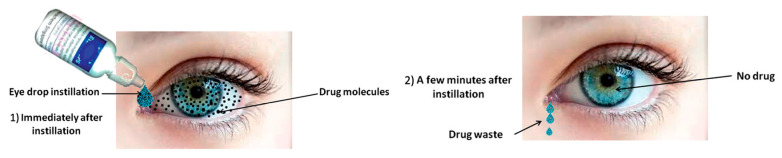
Drugs delivered through conventional eye drops. The main proportion of drug is typically removed after minutes whereas only a few percent absorbed into the eye. Reprinted from ref. [24]. Rights managed by Taylor & Francis.

Eye drops are the most frequently prescribed form of ocular treatment [3]. This is due to several inherent advantages of topical instillation of eye drops such as their non-invasive character and easy administration, resulting in high patient compliance and their immediate action. Despite their convenience, it is generally accepted that due to the ocular barriers, only 5% of the administered drug actually reaches its target, as shown in Figure 2 [4,5,6,7,8]. Moreover, only a small volume (approximately 30 μL) of eye drop formulation can be instilled in the eye. As a result, concentrated solutions have to be administered, and frequent instillations are necessary to reach a therapeutic result [2,9,10,11]. Systemic delivery is an alternative delivery method, yet not an ideal one as other limitations arise: first-pass metabolism, side effects, blood–ocular barriers, low eye vascularization, etc. In a quest toward more efficient drug delivery systems, the efforts have focused on increasing the ocular bioavailability of topical formulations. The generally accepted and less harmful solution to improve therapeutic treatment and drug bioavailability is to the increase the residence time of drop formulations on the ocular surface, thus increasing drug concentration on the cornea and reducing drug waste [12]. This has been achieved with drug-impregnated contact lenses, patches, colloidal carriers (nanoparticles, different kind of liposomes, etc.,), microneedles, films, gels, or solutions of mucoadhesive polymers, which is the simplest method [3,13,14,15,16,17,18,19,20,21,22,23]. A variety of materials has been used to prepare these smart delivery systems: natural or synthetic polymeric matrices, which can be modified and tuned for specific applications. Chitosan is one of them.

Chitosan (CS) is a hemi-synthetic cationic linear polysaccharide made of randomly distributed D-glucosamine and *N*-acetyl-D-glucosamine units, linked via β-(1→4) glycosidic bonds. It is synthesized by the deacetylation of chitin, which is a naturally occurring polysaccharide. CS is water-soluble in acidic pH due to the protonation of the free amino groups. It is an easily accessible polymer, but most importantly it is non-toxic, highly biocompatible, and biodegradable, and it has a low immunogenicity [25]. As such, it is an excellent candidate for pharmaceutical and biomedical applications [26]. Besides, several CS-containing products have already been approved by the US Food and Drug Administration. CS has inherent antibacterial properties, a mucoadhesive character [27], and can disrupt epithelial tight junctions, thus acting as a permeability enhancer. Along its polymeric chains, CS bears free hydroxyl and amino groups, which can be used for its chemical modification, allowing for an easy tuning of its physicochemical properties. Finally, CS can easily be formulated in a wide range of forms such as micro and nanoparticles, films, membranes, sponges, gels, etc. [28,29,30,31,32]. All these characteristics render CS a particularly well-adapted polymer for ocular drug delivery.

In the present review, we will discuss the properties of CS that contribute to its successful application in ocular delivery, before reviewing the latest advances in the use of CS for the development of novel ophthalmic delivery systems. In the first part, colloidal nanocarriers will be presented, followed by CS gels and finally lenses and ocular implants. CS has also been used as a coating, to confer mucoadhesiveness to other matrixes, and such examples will be discussed as well.

## 2. Chitosan Properties

### 2.1. Mucoadhesion of CS and Its Derivatives

Several synthetic and natural polymers have mucoadhesive properties. These properties can be affected by numerous factors, such as the polymer’s chemical structure and the reactive groups concentration, which affect the hydrogen or ionic bonding ability, its molecular weight, chain flexibility, and its swelling and hydration ability. Mucoadhesive polymers have numerous reactive or charged groups (–COOH, –NH_2_, –OH, –SO_3_H, etc.) that are able to form non-covalent bonds with mucin and adhere to the mucosal surface. This process is called bioadhesion [33,34,35]. However, the mechanism of bioadhesion is not so simple and in order to understand it better, the mucus structure should be discussed beforehand.

Mucus is a weak viscoelastic gel that adheres to and covers the internal tracts of the body. Its main functions are to protect and lubricate epithelium damage and to impair microorganisms and other substances from passing into the body. From several studies, it was found that it consists of a mixture of water (about 95 wt %), 0.5–5 wt % glycoproteins (mucins), 0.5–1 wt % other proteins, about 1 wt % inorganic salts, and some small amounts of lipids and mucopolysaccharides [36]. Even though mucins are the main structural component of mucus, their detailed composition remains unclarified. However, it is estimated that the glycoproteins of mucin have a molecular weight ranging from 500 kDa to 20 MDa and are able to form a gel matrix due their association with each other via non-covalent hydrophobic interactions, hydrogen bonding between sugar units, and disulfide linkages between cysteine residues. This gel is responsible for the viscoelastic properties of mucus.

Mucus, due to the carbohydrate-bound ester sulfate residues and the carboxyl groups of sialic acids on the mucin proteoglycans, has a net negative charge. The glycosylated regions of mucins are hydrophilic, whereas the non-glycosylated protein domains are hydrophobic. The interactions between mucoadhesive polymers and the mucus surface involve first the wetting and adsorption of the two surfaces to create an intimate surface contact. This first step is promoted by the hydrophilic groups that the mucoadhesive polymers bear, creating non-covalent bonds such as hydrogen or ionic bonds with mucus charged groups. In a second step, polymeric macromolecules and mucus glycoproteins can interfuse or interpenetrate to a certain extent across the formed interface, thus strengthening the mucoadhesion contact.

Even though ionic and hydrogen bonding interactions (electronic interactions) are responsible for this adhesion, there are several other theories proposed to explain the mucoadhesion mechanism between a polymer and mucus, including: wetting, adsorption, diffusion interlocking, mechanical and fracture theory [37,38,39,40]. Most of them, as already discussed, accept that two phenomena such as surface energy thermodynamics and interpenetration or diffusion are responsible for enhanced mucoadhesion [41,42,43].

Numerous in vitro methods have been developed to evaluate the mucoadhesive properties of a polymer or pharmaceutical formulation [44,45]. Most methods are based on calculating the mucoadhesive strength by mechanical tests, and mainly by measuring the necessary force for the detachment of the formed interface between the mucus and polymer [40]. Incubation time is very important in order to allow enough time for hydrophilic and hydrophobic interactions to develop and achieve substantial interaction between a mucoadhesive polymer and a mucous surface. As reported in a recent study, after 10 min of incubation, the number of adhered CS nanoparticles (NPs) on a mucous surface is much higher, compared to a one-minute incubation or no incubation at all [46]. Furthermore, after washing, a longer incubation time results in an increased number of NPs remaining on the mucous surface.

Due to its cationic nature, CS has been extensively used as a mucoadhesive polymer [47,48,49]. The mechanism of molecular interactions between mucin and CS has recently been thoroughly described [50]. It was reported that mucoadhesion depends on external factors that include environmental conditions, such as the pH, the concentration, ionic strength, the ratio between macromolecules, the temperature and incubation time, as well as the properties of mucin (kind of mucus) and CS, such as surface charges, molecular weight, spatial conformation, flexibility of macromolecular chains, degree of deacetylation, ability of hydration and swelling, etc. [51]. CS and mucins interact predominantly electrostatically, yielding protein–polysaccharide complexes. However, the mucoadhesion of CS is pH-dependent. At very low pH, such as 1.2, CS shows poor mucoadhesion, since though its amino groups are completely protonated to –NH_3_^+^, the –COOH and –SO_3_H groups of the sialic acids of mucins are uncharged. At pH close to 4.5, the amino groups of CS are still protonated and thus electrostatic interactions such as ionic bonds take place with the negatively charged carboxylic and sulfate groups of mucin, which is the main mechanism of CS mucoadhesion [52]. At pH 7.0, all CS amino groups are deprotonated and their ability to electrostatically interact with the carboxylic and sulfonate groups of mucins is limited. Due to this behavior, it has been mentioned that mucoadhesive interactions are stronger at pH of 5.2 than 6.3; the calculated work forces of adhesion were 0.3–0.5 mN and 0.2 mN, respectively [53]. So, it is accepted that the protonated amino groups of CS are responsible for its mucoadhesivity, and when the number of these groups is reduced, as in the case of half-acetylated CS derivative, mucoadhesion is reduced, too [54,55]. In addition, electrostatic interactions cannot take place at neutral pH where the only existing interactions are hydrogen bonding and weak van der Waal’s forces.

The mucoadhesion strength of CS is 0.58 N/cm^2^, which is slightly lower than that of hydroxyethylcellulose (0.88 N/cm^2^) and much lower compared with poly(vinyl alcohol) (PVA) (5.11 N/cm^2^) [56]. However, it is high compared with poly(vinyl pyrrolidone) (PVP), which has a negligible mucoadhesive force, (6 mN/cm^2^), and hydroxypropyl methylcellulose (HPMC) (157 mN/cm^2^) [57]. In another study, the detachment force of neat CS from pig intestinal mucosa was found to depend on the kind of CS and ranged between 3.9 (low viscosity) and 6.7 mN/cm^2^ (high viscosity) [58]. In the same study, it was found that poly(acrylic acid) (PAA) had better mucoadhesive properties (11.7 mN/cm^2^), while other natural polymers such as pectin, starch and xanthan gum were not mucoadhesive. However, for PAA, much different mucoadhesive strength values have been reported such as 26 mN/cm^2^, which is comparable with that of poly(2-ethylhexyl acrylate) (21 mN/cm^2^) [59]. In the latter study, the effect of film contact time to mucus surface (10–300 s) and crosshead speed used for detachment (3–30 mm/min) were evaluated, and it was proved that the mucoadhesive strength increased by increasing both variables.

Taking into account that secondary bonding arises mainly from the existence of reactive groups, comparing CS and other mucoadhesive polymers, it was observed that those with –COOH groups such as carboxymethyl cellulose (CMC), alginic acid, and PAA had much better mucoadhesive properties than neat CS [51]. Frequently used mucoadhesive polymers have been classified according to their mucoadhesion strength: poly(acrylic acid)s (1–3.8 N/cm^2^) > alginate (1.1–2.8 N/cm^2^) > CS (0.42–0.85 N/cm^2^) > CMC (0.1–0.4 N/cm^2^) = HPMC (0.1–0.34 N/cm^2^) > gums (xanthan, badam, gellan, guar) (0.08–0.3 N/cm^2^) [60]. However, in another comparison of mucoadhesive properties of different polymers such as 2-hydroxyethyl ether cellulose, cellulose, HPMC, Kollidon VA 64, CS, carbopol 974 P NF, and Noveon AA-1, it was found that 2-hydroxyethyl ether cellulose and CS had the highest values of adhesion work, and that was attributed to the higher wettability of these polymers [61].

Besides its lower mucoadhesive strength, at neutral pH, CS has some additional limitations such as a low water solubility and low swelling properties. For example, the mucoadhesive strength of neat CS was estimated to 0.34 N/cm^2^ [62], while PAA (Carbopol 934) had a much higher mucoadhesive strength (about 0.51 N/cm^2^) [63]. To overcome these problems, CS has been modified with appropriate monomers, which have additional reactive groups to ensure mucoadhesion at pH 7 [64,65,66]. This is very important for ocular release formulations, since eye mucus has a pH about 7.8 and thus can be characterized as slightly basic surface, while nasal mucus has a neutral pH and in gastric mucus pH ranges from approximately 1–2 to 7 with pH rising from the luminal to the epithelial surface [67].

Trimethyl CS (TMCS) is maybe the most studied CS derivative. Due to its high cationic charge (-N^+^(CH_3_)_3_), it is one of the strongest mucoadhesive polymers [68]. The adhesion strength of TMCS is about 7–11 N/m^2^, and its mucoadhesion is due to interactions taking place between its positively charged quaternary ammonium groups and the negatively charged sulfate and sialic acid groups of mucosa [69]. An additional advantage of TMCS is its solubility in neutral and basic pH due to the alkylation of all amino groups with methyl groups, resulting in permanent positive charges. There are also amphoteric derivatives such as carboxymethyl CS, which due to the existence of both carboxyl and amino groups can act as an acidic or basic material, depending on the pH [70,71]. CS derivatives with additional reactive groups were also found to have enhanced mucoadhesive properties. For example, derivatives prepared with methylacrylate and acrylic acid (AA) exhibited improved mucoadhesion. This is due to the strong hydrogen bonds that the –COOH groups of AA can form with the –COOH and –SO_3_H groups of mucus glycoproteins; the improvement was much higher at pH 4 than at 6.4. For this reason, PAA is reported to have the highest mean adhesive force amongst other polymers [72].

It has been suggested that weak ionic or hydrogen bonding interactions between CS reactive groups and mucus groups are not able to provide sufficient mucoadhesion. For this reason, thiolated CS derivatives have been extensively studied [73,74,75,76,77,78,79,80]. These derivatives have free thiol groups that lead to the formation of covalent bonds with cysteine-rich subdomains of mucus glycoproteins [81]. Due to these stable bonds, thiolated CS derivatives possess excellent mucoadhesive properties and can increase the residence time of a polymer on a mucus surface. However, these polymers can only be applied in tissues that have a cysteine-rich mucus layer, since they need sulfhydryl groups to react. When N-hydroxysuccinimide was added on a polymer backbone, stable covalent bonds were formed with mucus amino groups, indicating that this could be an alternative method to prepare novel mucoadhesive excipients [82].

Similar interactions were also suggested between CS derivatives grafted with poly(ethylene glycol)diacrylate and a mucus surface [46,83]. These polyacrylate derivatives have free vinyl end groups, which may interact with the thiol groups of a mucin glycoprotein by a Michael-type reaction, forming covalent bonds. Due to these bonds, it was found that acrylated CS had better mucoadhesive properties than both CS and thiolated CS derivatives [84]. Other CS derivatives with catechol showed also enhanced mucoadhesive properties compared with CS due to the molecular complexation between the formed derivative surface and mucin [85]. Similar complexations have been reported in CS methacrylated derivatives, which were also found to have enhanced mucoadhesive properties [86].

Mucoadhesion seems to be a very promising approach to enhance drug effectiveness. However, there are some fundamental limitations to drug delivery through mucosal tissues [87]. Firstly, due to its high hydrophilicity and viscosity, mucus has multiple barrier properties and does not favor the diffusion of hydrophobic drugs [88]. Secondly, even though mucus gel is generally a stable system, it has a very short lifetime, since it is reformed dynamically through the secretion of mucins from goblet cells. It was found that the clearance period is about 5.0–7.7 min in the eye, 10–20 min in the respiratory tract, and much higher in the gastrointestinal tract: 4–6 h [39,89,90]. Thus, for several delivery systems, the mucoadhesive particles are not expected to adhere to mucus for long. Furthermore, despite the high mucoadhesive properties of some polymers, the capacity of mucus to immobilize foreign particles may become saturated. In other words, particles can only adhere on a mucus surface until the available surface area for adsorption is saturated [91]. It is currently accepted that while mucoadhesion is important to achieve drug bioavailability, the ability of polymeric structures to penetrate into mucus also plays an important role for drug effectiveness [92].

### 2.2. Antibacterial Properties

CS has antimicrobial activity since it can disrupt or destabilize the barrier properties of the outer membranes of Gram-negative bacteria or permeate the microbial plasma membrane [93,94,95,96]. The mechanism is based on interactions taking place between the positively charged amino groups of CS and the negatively charged microbial cell membranes [97]. These ionic interactions disrupt the microbial membrane, ultimately resulting in a leakage of intracellular constituents. The concentration of CS is very crucial, since when it is used at <0.2 mg/mL, the amino groups of CS interact with the negatively charged bacterial surface, leading to agglutination [98]. At higher concentrations, the number of CS amino groups is too high and can form a net positive charge onto the bacterial surfaces, resulting in a suspension.

CS derivatives have also antimicrobial properties against various microorganisms and, depending on the side groups, these properties can be substantially enhanced [97,99]. It is well known that the quaternization of CS can result in derivatives with enhanced antibacterial properties against several negative and Gram-positive bacteria compared to neat CS [100,101,102,103]. When comparing the inhibition of neat CS, TMCS, and *N*-diethylmethyl CS (DEMC) against *Staphylococcus aureus*, it was found that TMCS had the highest inhibition effect, followed by DEMC and CS [104]. This was attributed to the stronger positive charge of quaternary ammonium groups in TMCS compared with CS. These groups form strong polyelectrolyte complexes with negative peptidoglycans of the bacterial cell wall, leading to cell wall disruption and thus bacterial death. Quaternary CS derivatives prepared with 2-hydroxypropyltrimethyl ammonium chloride also showed high antimicrobial activity against *S. aureus* and *Escherichia coli* [105]. Furthermore, it was found that the antimicrobial activity increased by increasing the degree of quaternization. *N*,*N*,*N*-Trimethyl-*O*-(2-hydroxy-3-trimethylammonium propyl)-chitosans with different degrees of *O*-substitution were synthesized and demonstrated enhanced bacteriostatic properties against *E. coli* and *S. aureus* [106].

Except for quaternary CS derivatives, it was found that when carboxyl groups were grafted on CS chains, such as in the case of *O*-carboxymethyl CS (CMCS), antibacterial properties were further enhanced [99]. The introduction of sulfoxyamine groups in CS resulted in a derivative with simultaneously enhanced mucoadhesive and antibacterial properties [107].

### 2.3. Penetration Enhancement

Apart from antimicrobial and mucoadhesive properties, it was found that CS has also penetration-enhancing properties, since it can open the tight junctions located in the epithelial cells [108]. Cornea and conjunctiva have a negative charge; thus, the amino groups of CS could interact with these extraocular structures. This interaction could increase the drug concentration and its residence time, resulting in a higher accuracy of instilled drop solution and reproducibility of dosing [109,110]. According to numerous research works, CS-coated NPs can prolong the residence time in cornea and also enhance the intraocular penetration of drugs [111,112,113]. In a previous work, four quaternized CS derivatives (TMCS, dimethylethyl CS (DMEC), DEMC, and triethyl CS (TECS)) were prepared, and their properties as penetration enhancers evaluated [114]. It was found that transepithelial electrical resistance increased following the order: TMCS > DMEC > DEMC = TECS > CS, indicating their ability to open the tight junctions. In a comparative study, Mei et al. investigated the nasal absorption promoting effect of 2,3,5,6-tetramethylpyrazine phosphate using CS, TMCS, and thiolated CS of different molecular weights, for intranasal absorption [115]. It was found that TMCS was the strongest absorption enhancer, followed by neat CS, while thiolated CS could not improve the absorption properties of CS.

## 3. Nanocarriers

### 3.1. Nanoparticles

CS NPs are particularly appropriate for ocular delivery due to their nanometric size and mucoadhesive properties [15,116,117]. Drug formulations in NPs show many advantages compared to conventional delivery systems, such as particle size control, protection of the active substance from in vivo and in vitro degradation, targeting, improved therapeutic effect, prolonged biological activity, controlled drug release rate, and a decreased frequency of administration [118,119]. One additional advantage is that due to the high specific surface area of NPs, their mucoadhesion is much higher compared to bulk polymers [120]. Owing to the high specific area of NPs, the available interface for hydrogen bonding, ionic bonding, or hydrophobic interactions with a mucus surface increases dramatically. It was found that drug-loaded NPs with sizes ranging between 50 and 500 nm are the most suitable for ocular drug delivery [121]. NPs in this range have the ability to overcome ocular physiological barriers and penetrate the mucin mesh [122,123], while NPs with sizes higher than 1000 nm can only superficially adhere on mucus, due to their inability to fit in mucus channels [91]. These, or larger particles, which do not adhere, are cleared from mucus surfaces. On the other hand, a recent study by Ding et al. with CS-coated poly(lactic-*co*-glycolic acid) (PLGA) NPs pointed out a threshold diameter under which mucoadhesion does not increase any more independently of the decreasing particle size [124]. It is generally accepted that particles over 10 μm can cause ocular irritation and provoke a “foreign body sensation” to the patient. NPs possess the merits of simple administration in fluid formula and swift dispersal through ocular tissues. The encapsulation of ophthalmic drugs in CS NPs and its use in solution formulations was found to significantly increase the residence time of antibiotic drugs in the precorneal area [111,125,126].

Multiple methods have been used to produce CS NPs including ionotropic gelation, spray drying, water-in-oil emulsion crosslinking, reverse micelle formation, emulsion droplet coalescence, nanoprecipitation, or self-assembly [127]. The selection of the most appropriate method should take into account the physicochemical characteristics of the drug (hydrophilic, lipophilic, ionic…) and the specific application that dictates the NP requirements, personnel safety, etc. Amongst these techniques, ionotropic gelation is generally preferred due to its relative simplicity and convenience. Non-toxic solvents/excipients and safe crosslinkers are employed, while high temperature is avoided.

#### 3.1.1. Ionotropic Gelation with Sodium Tripolyphosphate

##### Nanoparticle Formation

The mechanism of ionotropic gelation is based on ionic interactions between the positively charged amino groups of CS and anionic small molecules, e.g., sodium sulfate, sodium tripolyphosphate (TPP), or anionic polymers, e.g., hyaluronic acid, alginates, chondroitin sulfate, and sodium cellulose sulfate, which causes the formation of inter- and intramolecular crosslinks, resulting in the formation of NPs. Bodmeier et al. were the first to investigate the ionotropic gelation of CS with TPP [128], and while various polyanions have been reported in the literature for the preparation of CS NPs by ionic gelation since then, TPP is by far the most commonly used one.

According to the generally applied experimental protocol, CS is dissolved in aqueous acetic acid (pH about 4.5). TPP is dissolved in water separately, and the TPP solution is added dropwise to the CS solution under continuous stirring. The active pharmaceutical ingredient (API) is separately dissolved in water or an organic solvent and added to either CS or TPP solutions. For example, Silva et al. added daptomycin in both CS or TPP solutions without noticing significant differences in the obtained NPs [129]. Finally, NPs are washed and collected by centrifugation and/or freeze-drying. The particular details of this general experimental procedure vary amongst research groups, giving very different results. Some indicative experimental details and NPs characteristics are summarized in Table 1.

**Figure 3:**
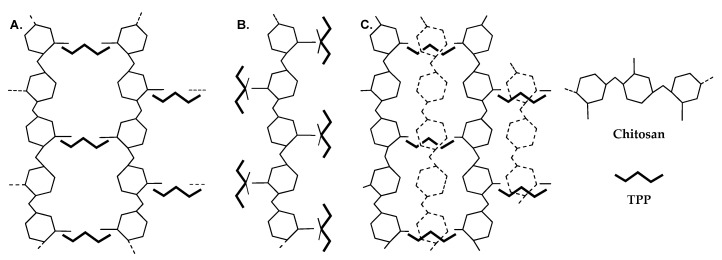
Simplified schematics of the primary ionic crosslinking configurations (**A**) H-link and (**B**) T-link. Combinations of primary linking types lead to (**C**) secondary linking types. In configuration (**C**), the dashed monomer structures are off-plane and form T-links with the tripolyphosphate (TPP) units below them. Reprinted with permission from ref. [130]. Copyright 2012, American Chemical Society.

When CS is dissolved in acidic media, the amino groups are protonated, and CS chains are thus subjected to repulsions between positively charged nitrogen atoms, as well as attractive forces due to the formation of hydrogen bonds. When TPP is added, the formation of CS NPs results from the interactions between the negatively charged phosphate groups of TPP and the positively charged amino groups of CS [130,141]. Koukaras et al. using all-electron density functional theory studied the molecular interactions taking place during the ionic crosslinking of CS with TPP [130]. A multitude of CS–TPP configurations were studied, and three types were identified as possible configurations. In the first crosslinking configuration (H-link, Figure 3A), a TPP molecule acted as a linking axis between two CS chains, which were oriented almost perpendicularly to the TPP molecule, forming a ladder-like structure. This configuration was presumed to be the most probable on the basis of the high interaction energy that was calculated for it and the accessibility of TPP allowing such interactions. In the other two possible configurations, CS and TPP were parallel and TPP interacted with CS amino groups at one or two points. In the first case, a single interaction was described with the nonbridging oxygen atom bonded to the central phosphorus atom (T-link, Figure 3B). In the second case, two individual interactions, stemming from two oxygen atoms, one on each side of TPP, took place with CS (M-link). Of course, a combination of those resulted in secondary linking patterns (Figure 3C). The occurrence of such interactions depended on the CS/TPP ratio, and their density affected nanoparticle formation (vide infra).

When it comes to drug delivery, size is one of the most important characteristics of NPs. Smaller particles penetrate more easily and offer a greater surface. In parallel, the particle size of ocular formulations should not exceed 10 μm in order to avoid ocular irritation and patient discomfort. CS NPs have been prepared in a wide range of sizes (100–700 nm), and numerous research groups have studied how experimental conditions (CS and TPP concentrations, stirring, pH, etc.) affect the size of NPs.

A direct correlation of CS and TPP concentrations with particle size has been observed. Most authors report that increasing CS concentration results in an augmentation in particle size [133,135,136,140,142]. As CS concentration increases, CS molecules are closer to each other, resulting in an increase in intermolecular crosslinking (H-bonding) and thus larger particles [133,143,144]. However, the formation of smaller, more compact particles [138,145] has also been observed, as well as an initial decrease in size followed by an increase or vice versa [139,144]. According to some authors, particle size is not significantly affected by CS concentration [129,132]. Similarly, an increase in TPP concentration has been reported to result either in an increase in NP size [133,140,142,146] or a decrease [129,132,145] due to stronger electrostatic interactions [132], or in variation [144].

**Figure 4:**
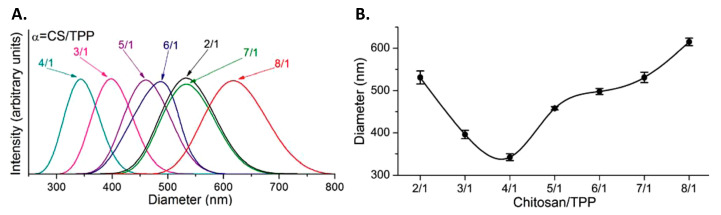
(**A**) Particle size distribution obtained by dynamic light scattering (DLS). (**B**) Extracted particle sizes at various chitosan (CS)/sodium tripolyphosphate (TPP) weight ratios. Reprinted with permission from ref. [130]. Copyright 2012, American Chemical Society.

Some authors have focused on the CS/TPP ratio rather than their isolated concentrations. Studying CS NPs’ formation in those terms should give a better understanding of the phenomenon as it is the interplay between CS and TPP that affects the formation of NPs rather than each one separately. A decrease in the CS/TPP ratio has been related to a reduction in particle size [129,135,139]. Koukaras et al., who studied a large range of CS/TPP ratios, from 2/1 to 8/1, observed an initial decrease in the particle size of the prepared NPs followed by an increase, as illustrated in Figure 4 [130]. They did not interpret this trend in terms of CS/TPP ratio but rather in terms of the CS monomer/TPP ratio. According to them, a low CS monomer/TPP ratio (high TPP concentration) led to a dense crosslinking. As a result, individual CS chains were linked in longer nanofibers that ultimately led to the formation of larger and stiffer NPs. A higher ratio, up to 4/1 CS/TPP, resulted in smaller, less stiff NPs. A further increase (low TPP concentration) produced big NPs with a low density of crosslinks due to the low amount of TPP. In accordance with those observations, Morsi et al. have reported that TPP concentration should be higher, within certain limits, than CS concentration to obtain NPs [138]. Otherwise, no particles ([TPP] ≤ [CS]) or a precipitating suspension ([TPP] >> [CS]) were obtained. The outcome of Chiesa et al. work is also along those lines, demonstrating that intermediate CS/TPP mass ratios are more beneficial to the formation of smaller particles [146]. Finally, Han et al. rationalized the formation of NPs based on the molar ratio of nitrogen in the repeating unit of CS and phosphorus in TPP [147,148]. According to them, when the ratio is around 1, large unstable particles were obtained (1–10 μm), while increasing this ratio above 2 at least allowed the formation of more stable particles in the nanometric range (200–400 nm).

The seemingly contrasting results reported in the previous paragraphs may be attributed to differences in the concentration ranges of CS and TPP, different CS molecular weights or deacetylation degrees, as well as other differences in experimental protocols. Another important observation is that as the molecular weight of CS increased, the size of the NPs increased too, since longer molecular chains entangle together, resulting in a larger size [131,136,149]. Finally, API entrapment usually results in an augmentation in size compared to blank NPs.

The influence of the stirring rate and duration in the preparation of CS NPs was also studied [132,133,137,144,150]. Generally speaking, stirring at 100–1200 rpm for 10 min to 18 h has been reported. In contrast to Kalam et al. [133] and Barwal et al. [144], Lazaridou et al. [137] and Badiee et al. [132] reported that stirring duration and speed did not significantly affect the size of NPs. While investigating the feasibility of applying microfluidics to the preparation of CS NPs, Chiesa et al. noted that an increase in the total flow rate resulted in significantly smaller NPs [146]. This was correlated to the mixing time, which affected NPs aggregation as well as the balance between inter- and intra-molecular crosslinking and hence the compactness of the obtained NPs. CS NPs formation by ionotropic gelation occurs between charged species and, thus, not unexpectedly, pH was also reported to affect this process [141].

Computer-assisted modeling has been used to rationalize the influence of experimental parameters, resulting in equations that would allow predicting the final particle size or entrapment efficiency depending on CS, TPP concentrations, etc. [132,133,139,140,145]. Indicatively, Kalam et al. reported the optimization of CS NPs employing Box–Behnken surface methodology to evaluate the effect of TPP (*A*) and CS (*B*) concentrations and stirring time (*C*) (independent variables) on particle size, amongst other characteristics [133]. The generated model was expressed by the following equation:

*R* = 413 + 25.63*A* + 84.62*B* + 6.75*C* − 45.0*AB* + 27.25*AC* + 47.75*BC* + 17.4*A^2^* + 82.4*B^2^* + 21.65*C^2^* (1)

where *R* is the particle size, and *A*, *B*, *C* the independent variables, as defined previously. The terms *AB*, *AC,* and *BC* represent the interactions, while *A^2^*, *B^2^*, and *C^2^* are the quadratic terms. A positive value of regression coefficients was interpreted as a synergistic effect, while a negative value pointed out an antagonistic effect.

The synthesis of ultra-small particles (<50 nm) is more difficult. Sunkireddy et al. [150] and Barwal et al. [144] prepared ultra-small CS NPs by tuning the mechanochemical parameters in order to increase intermolecular distance and decrease interparticle crosslinking. Specifically, a small beaker (25 mL) and big magnetic stirrer (1.0 × 0.5 × 0.5 cm) were used, as well as the highest possible stirring speed (650 rpm). The height of CS solution was just enough to cover the magnetic stirrer. The TPP solution drops were 1.0 μL and the dropping speed was 1 drop/s. By applying these mechanical parameters, NPs of 20 ± 5 nm were obtained from 10 mL of 0.225% *w/v* CS solution and 5 mL of 2 mg/mL TPP solution.

Except for the size, other characteristics are important. The polydispersity index (PDI) is a measure of the homogeneity of the prepared NPs. PDI values that have been reported for CS NPs range between 0.17 and 1, with values over 0.7 indicating rather broad distributions. PDI has been found to be dependent on CS and TPP concentrations. Generally, as either concentration increases, the PDI increases [133,136,140,146]. Badiee et al. reported a slight increase in PDI from 0.25 to 0.42 when the CS concentration increased, but there was an important reduction from 0.41 to 0.24 when the TPP concentration increased [132]. Finally, according to Sabbagh et al., the highest PDI was predicted for simultaneous very high or very low CS and TPP concentrations [139].

ζ-potential is a measure of the surface charge of NPs. NPs having high absolute values of ζ-potential (>±30 mV) are more stable due to higher repulsions between NPs and present a smaller risk of particle aggregation. In the case of ocular delivery, prolonging drug residence on the ocular surface is critical. The mucoadhesion of CS is promoted by the positively charged amino groups that interact with the negatively charged corneal mucin layer. Thus, high positive ζ-potentials are generally advantageous. The ζ-potential of CS NPs range from 20 to 60 mV and is affected by the CS/TPP ratio, since they are oppositely charged. CS NPs have positive ζ-potentials due to the cationic nature of CS. Increasing the concentration of TPP leads to a decrease of ζ-potential [133,138,139,140,142]. For example, Morsi et al. studied the changes of ζ-potential and found that its values decreased from +19.3 to +15.1 mV while increasing the concentration of TPP from 0.4 to 0.6 mg/mL [138].

The morphology of NPs is also an important characteristic, as spherical NPs possess a higher surface area-to-volume ratio compared to other shapes, resulting in a more reactive surface and ultimately, more opportunities to produce a therapeutic effect. As confirmed by scanning electron microscopy (SEM) and transmission electron microscopy (TEM) photomicrographs, CS NPs are generally spherical and smooth.

Due to the CS cationic nature, the swelling ability of NPs is affected by pH (and proportional to CS concentration) [133]. Important swelling has been observed around pH 7, accompanied by a deswelling at higher pH due to the modification of electrostatic repulsions, as the protonation of CS amino groups is pH dependent. Other authors report a better swelling at pH 5 rather than 7 [146]. In some cases, drug release kinetics fitting suggested that release was not only diffusion-controlled but also swelling-controlled (non-Fickian diffusion) [131,133,151].

##### Drug Encapsulation and Release

CS NPs as a drug delivery system have the ability to adhere to mucous surfaces and thus be retained for a prolonged period on the ocular surface. As a result, CS NPs are excellent agents for API sustained release in topical applications. An ideal drug delivery system should have suitable size, enhanced stability, and controlled release, as well as being non-toxic. Furthermore, drug loading and entrapment efficiency are also critical when engineering NPs: high drug loading percentages result in more efficient NPs, while high entrapment efficiency reduces drug waste during formulation.

Both drug loading and entrapment efficiency are affected by CS and TPP concentrations [133]. Drug loading depends also heavily on the concentration of the drug used during the preparation of NPs. Increasing drug concentration results in a higher loading [139,140,151], although only up to a certain value [129,142]. An improvement in API entrapment efficiency has been reported with increasing CS [139,140,145] and TPP [145] concentrations. This may be attributed to the fact that more polymers can encapsulate more drugs, and more TPP accelerates entrapment by increasing the crosslinking contact [145]. As expected, decreasing the CS/TPP ratio leads to a decrease of entrapment [136,139,145].

Several drugs have been loaded in CS NPs aiming at treating various eye conditions: levofloxacin [135,152,153] and diclofenac [131] as antibacterial factors; lomefloxacin [140], ciprofloxacin [154], moxifloxacin hydrochloride [155], acetazolamide [136]; brimonidine for glaucoma therapy [144]; ganciclovir [156], indomethacin [133]; L-2-oxothiazolidine-4-carboxylic acid, which is involved in cataract treatment [157]; ketorolac tromethamine [138,142,145], bevacizumab [132], ranibizumab [158], celecoxib [159], doxorubicin [147,148,160], metronidazole [139]; dexamethasone [143,151,161], which is anti-inflammatory; bovine lactoferrin [150]; daptomycin for the treatment of bacterial endophthalmitis [129]; tobramycin sulfate [162], curcumin [146], rosmarinic acid [163], cardamom essential oil [164], and plant extracts [163,165]. Those NPs have been either administered as such or incorporated into other drug delivery formulations (see Chapter 3.4).

Drug release largely depends on the physical state of the compound that is encapsulated or dispersed in the polymeric matrix. It also depends on the extent of interactions with the polymeric matrix and particularly the ability to form hydrogen bonds. In most cases, a biphasic drug release is observed: an initial burst release, which is due to the release of superficially adsorbed molecules, followed by a sustained release of the active principle. The burst release may be advantageous to rapidly achieve a therapeutic concentration, followed by the sustained release to maintain the therapeutic concentration. Release is dominated by diffusion of the API molecules out of the NPs, but it can be assisted by dissolution of the drug in the release medium, the swelling of the particles, and some hydrolytic degradation/erosion of the CS matrix.

Levofloxacin shows good antibacterial activity on cornea and conjunctiva. In the work of Ameeduzzafar et al., levofloxacin was encapsulated in CS NPs, which were further incorporated in an in situ gelling formulation. Levofloxacin was entrapped in an amorphous state, and good entrapment efficiencies were achieved (up to 80%), although the final drug loading was low (3–5%) [135]. Similarly, Kong et al. prepared CS microspheres which were loaded with levofloxacin and incorporated to a CS-based hydrogel [153]. Diclofenac sodium is another drug that has anti-inflammatory and antimicrobial activity. Diclofenac sodium was loaded in CS NPs using low and high molecular weight CS (LMWCS and HMWCS respectively). Encapsulation efficiency was slightly, though not significantly, increased by increasing the CS molecular weight (29.3% and 31.1% for LMWCS and HMWCS, respectively). The release rate of diclofenac sodium decreased when the molecular weight of CS increased. The antibacterial activity of diclofenac sodium-loaded CS NPs against *S. aureus* and *Bacillus Subtilis* depended on the molecular weight of CS and the pH of the medium, but it was higher than pure diclofenac sodium due to the synergy with CS [131]. Lomefloxacin hydrochloride is another widely used antibacterial agent. It was loaded in CS NPs with good encapsulation efficiency (57–69%) for ocular applications. The release of lomefloxacin hydrochloride from NPs was extended over 8 h (approximately 94% of released drug) while release from lomefloxacin hydrochloride powder was almost complete within 30 min [140]. Ciprofloxacin, another antibacterial factor, was also loaded in CS NPs from different sources (shrimps, locust, beetles, and honey bees). High entrapment efficiency was achieved (approximately 99%), and a 12-h drug release pattern was observed *in vitro*. The antibacterial activity was assessed against both Gram-positive and Gram-negative bacterial strains (methicillin-resistant *S. aureus*, *Pseudomonas aeruginosa*, *E. coli*, and *Bacillus thuringiensis*). Better performances were demonstrated for the drug-loaded NPs compared to either free ciprofloxacin or empty CS NPs [154]. The antibacterial drug metronidazole was also encapsulated in CS NPs. Ionic interactions between CS and metronidazole were evidenced by Fourier transformation infrared spectroscopy (FTIR). Biphasic release was observed and depending on formulations, drug release could be extended up to 24 h [139]. Daptomycin is a natural lipopeptide with antibiotic activities, which was loaded in CS NPs for the treatment of bacterial endophthalmitis [129]. High encapsulation efficiencies, up to 97%, were achieved. In vitro daptomycin was completely released within 4 h, according to a biphasic pattern. The antimicrobial activity of daptomycin was preserved after encapsulation, and the antimicrobial susceptibility of the drug-loaded CS NPs was satisfactory, although it was lower than for free daptomycin.

CS NPs have also been applied to the treatment of glaucoma. More specifically, acetazolamide was loaded in LMWCS and HMWCS NPs (entrapment efficiencies: 60–74% and 73–80%, respectively) [136]. A sustained in vitro release was observed compared to the free drug, which was attributed to dissolution and diffusion. Accordingly, intraocular pressure (IOP) reduction was observed in in vivo ocular hypotensive efficacy studies for at least 5 h. Similarly, brimonidine was encapsulated in ultra-small CS NPs for IOP reduction (encapsulation efficiency 39%) [144]. Ultra-small CS NPs revealed a prolonged release of brimonidine, with an initial burst release (0.5 h) and complete release within 100 h.

Ganciclovir [156], indomethacin [133], and L-2-oxothiazolidine-4-carboxylic acid [157] were also encapsulated in CS NPs for cataract treatment. Kalam et al. studied the encapsulation and in vitro release of indomethacin from CS NPs [133]. A fast initial release was observed for the first hour (30–50% of indomethacin release), followed by a slower release, which was attributed to a different release mechanism—i.e., diffusion instead of adsorption. Release wad fitted to the Higuchi kinetic model.

Ketorolac tromethamine is effective in inhibiting post-operative eye inflammation, reducing conjunctivitis with no alteration of cornea. Its ocular administration through CS NPs and other formulations has been extensively studied [138,142,145]. Reported entrapment efficiencies of ketorolac tromethamine in CS NPs were roughly 40–70%. Release behavior varied from 6 to 24 h, amongst the different groups that studied ketorolac tromethamine encapsulation. This could be attributed to different NP sizes and to different CS and TPP concentrations and ratios. Morsi et al. reported that an increase in CS or TPP concentration resulted in a decrease in the percentage of released drug [138]. Fathalla et al. reported that CS NPs had the ability to retain ketorolac tromethamine for a longer time on the ocular surface [142]. Finally, Morsi et al. further incorporated ketorolac tromethamine-loaded CS NPs in films that could be used as ocular inserts [138].

Dexamethasone is a well-known anti-inflammatory agent that has also been loaded in CS NPs for topical ocular administration. Kalam reported an entrapment efficiency around 70% for the optimized formulation [143,161]. These NPs were further coated by hyaluronic acid (HA) to contribute to the cellular uptake of NPs by receptor-mediated endocytosis. According to Behl et al., dexamethasone release from CS NPs followed diffusion and swelling controlled mechanisms and was impacted by the electrostatic interaction between the drug and the polymeric matrix [151]. In that work, dexamethasone-loaded CS NPs were ultimately used for the preparation of contact lenses that demonstrated a continuously increasing drug release (55.7%) for approximately 22 days (*vide infra*).

Bevacizumab, a drug that has demonstrated promising effects on choroidal neovascularization treatment, was loaded in CS NPs to increase its residence time on the ocular surface [132]. It is noteworthy that the drug release was extended over 60 days, with a low burst release. The total cumulative release was around 84%.

As evidenced by the above examples, drug encapsulation in CS NPs can contribute to a higher therapeutic efficiency in various ophthalmic diseases. It is also shown that the encapsulation and release of active agents depend on both the characteristics of the CS NPs and the nature of the drug.

#### 3.1.2. Ionotropic Gelation with Other Polymers

In the previous paragraphs, we focused our attention on the NPs produced by crosslinking CS with TPP; however, there are other polyelectrolytes that have been used to this intent as well: lecithin, pectin, sodium alginate, etc. (Table 2). Lecithin (L), a naturally occurring blend of phospholipids found in the cell environment, could potentially enhance loading and improve the release of lipophilic APIs. Chhonker et al. prepared CS/L NPs for the encapsulation of amphotericin B, which is a natural antibiotic that is widely used for the treatment of fungal keratitis [149]. The formation of CS/L NPs was based on ionic interactions between the phosphate groups of L and the amino groups of CS (ionic gelation). Three different molecular weights of CS were used and spherical NPs, ranging between 160 and 230 nm, were obtained. Furthermore, the increasing molecular weight of CS incrementally affected several properties of NPs, such as their size, ζ-potential, and entrapment efficiency. In vivo precorneal retention tests revealed increased bioavailability in comparison with the marketed formulation. Hafner et al. compared CS/L NPs and Pluronic F127/CS micelles for the ocular delivery of photosensitive melatonin, which is a neurohormone that has been proposed for IOP reduction [166]. The F127/CS micelles were formulated via a direct dissolution method and melatonin-loaded CS/L NPs by the injection of a lecithin alcoholic solution into a CS aqueous solution. Permeability was evaluated with an in vitro corneal epithelial model. The presence of CS in F127 micelles weakened the permeation-enhancing effect of the micelles due to the electrostatic interactions between CS and F127. On the other hand, CS/L NPs showed a controllable melatonin permeation efficacy. NPs resulted in an improved pre-corneal presence of melatonin, thus contributing to an enhanced ocular bioavailability and a prolonged IOP decrease.

**Figure 5:**
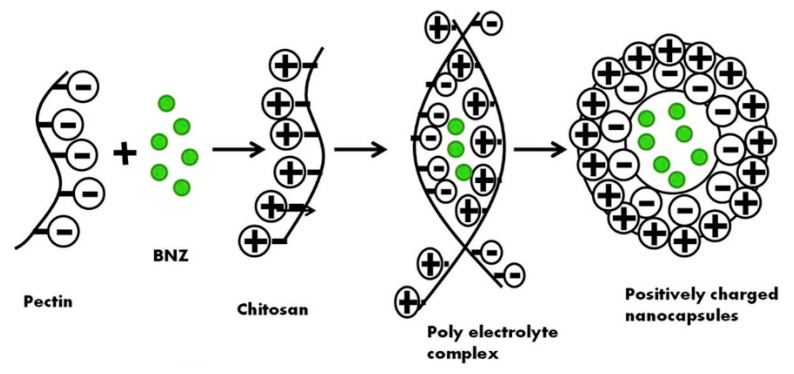
Formation of CS/pectin nanocapsules. Adapted from ref. [167]. Copyright 2019, with permission from Elsevier.

Pectin belongs to the family of anionic polysaccharides and is found in the cellular membranes of numerous plants. Due to its negative charge, pectin can be valuable for drug delivery polyelectrolyte complexes with cationic CS. In their work, Dubey et al. formulated CS/pectin nanocapsules to entrap brinzolamide, an anti-glaucoma agent, via a polyelectrolyte complex coacervation method, as illustrated in Figure 5 [167]. This study evidenced the impact of various factors on nanocapsule stability, such as polymer concentration, stirring factors, and pH, with the latter being the most important. In any case, the average size of the formulated particles was between 217 and 240 nm, while their shape was spherical and smoothed. The results revealed an enhanced brinzolamide corneal permeation from CS/pectin NPs, offering higher bioavailability and drug release, than the commercial eye drops. Likewise, Muhtadi et al. employed the electrostatic interactions between CS and pectin to prepare timolol maleate-loaded NPs, using calcium chloride, CaCl_2_, as a crosslinking agent [168]. A 2^3^ factorial pattern was designed for parameter optimization. More specifically, entrapment efficiency, particle size, and PDI factors were studied in correlation to CS, pectin, and CaCl_2_ concentrations. Based on these criteria, the highest desirability index (size 247–279 nm, PDI 0.67–0.69, entrapment efficiency 25–30%) was obtained by the systems prepared with 0.01% *w/v* CS, 0.4% *w/v* pectin and 0.2% *w/v* CaCl_2_. A prolonged drug release was achieved with this delivery system.

**Figure 6:**
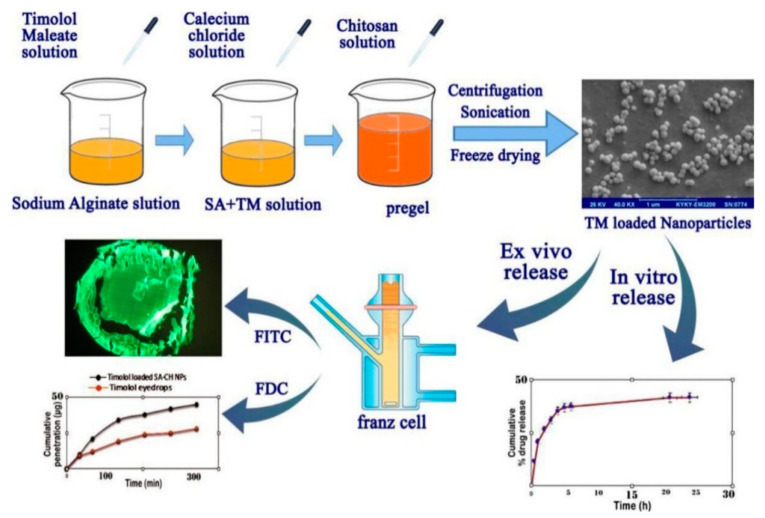
Preparation of timolol maleate-loaded chitosan/sodium alginate nanoparticles, in vitro and ex vivo release. Reprinted from ref. [169]. Copyright 2017, with permission from Elsevier.

The ionic interaction between CS and alginate is another interesting interaction. Alginate is a natural, hemocompatible anionic polysaccharide bearing carboxyl end groups, which is found in seaweed [12]. Sodium alginate (ALG) is a highly hydrophilic, mucoadhesive polymer that has a good biocompatibility and can act as a penetration enhancer and, as a result, it is largely employed for biomedical applications. In this context, Ilka and her coworkers capitalized on the pre-gelation method for the formulation of CS/ALG NPs encapsulating the anti-glaucoma drug timolol maleate (Figure 6) [169]. NPs were obtained from a low concentration ALG solution containing timolol maleate by inducing an ionotropic pre-gelation with calcium ions (CaCl_2_), followed by polyelectrolyte complexation with CS. CS/ALG NPs exhibited good stability, high entrapment efficiency, and sustained drug release. Similarly, Ahdyani et al. prepared CS/ALG NPs for timolol maleate ocular delivery [170]. The pre-gelation method with CaCl_2_ was applied as calcium ions contributed to the formation of more stable and compact NPs. NPs exhibited sizes in the range of 114 to 509 nm, while their size was proportionally influenced by the concentrations of CS and ALG. In vitro release studies evidenced a prolonged release over 24 h. Taghe et al. exploited the ionic synergy among CS, TPP, and ALG to formulate nanoparticulate systems for the incorporation of ofloxacin, an anti-infection agent, and compared its ocular dynamic against CS/TPP NPs [126]. In both cases, roughly spherical particles with positive surface were formed, while in the case of CS/ALG/TPP NPs, increasing the ALG content led to less stable dispersions. NPs showed an initial ofloxacin burst release followed by a sustained release; ocular penetration through sheep cornea compared to ofloxacin solution was improved. Abdelrahman et al. compared TPP and ALG in the formation of NPs for the delivery of lomefloxacin [140]. NPs prepared with ALG had a higher drug entrapment, but particles prepared with TPP exhibited higher permeation characteristics and a higher release. The optimized formulation, based on ALG, showed a better antimicrobial activity than lomefloxacin solution, which was attributed either to the presence of CS and/or to the overall higher efficiency of the formulation. Gong et al. investigated CS/calcium alginate NPs as nanocarrier systems for the delivery of low-density lipoprotein–rosiglitazone complexes, aiming at a prolonged released and a reduction of toxic adverse side effects [171]. Specifically, the lipoprotein–rosiglitazone complex was synthesized via an incubation exchange method and then added to CS/calcium alginate NPs. Comparable performances to rosiglitazone and slower release made this innovative system a promising rosiglitazone carrier to treat post-glaucoma scarring complications.

Naturally existing gums and their products are extensively investigated in pharmaceutical technology not only due to their biodegradability, biocompatibility, and non-immunogenicity, but also because of their non-toxic, cheap, and accessible nature [12]. Modifications of natural gums often ameliorate their water solubility, which is a desirable property for a variety of drug delivery applications. Minkal et al. employed a carboxymethylated derivative of gum katira, which exhibits a higher aqueous solubility [172]. Due to their opposite charges, carboxymethylated gum katira formed a polyelectrolyte nanoparticulate system with CS, which was used to entrap and deliver ofloxacin. The complexed NPs possessed an optimal size and good entrapment efficiency. The results indicated the enhanced in vitro corneal permeability of ofloxacin through isolated porcine cornea, while the nanosuspension’s corneal biocompatibility was approved by histological studies. In line with this work, Mittal et al. studied the interaction of CS with another gum, flax seed gum, to prepare CS/flax seed gum NPs loaded with timolol maleate for glaucoma treatment [173]. The ionic gelation technique was employed for this purpose. The bioadhesive polymeric NPs revealed sustained timolol maleate release, while showing advanced corneal penetration in comparison to marketed ophthalmic solutions. NPs were proven to be biocompatible with cornea and exhibited reduced IOP for a long duration after administration.

**Figure 7:**
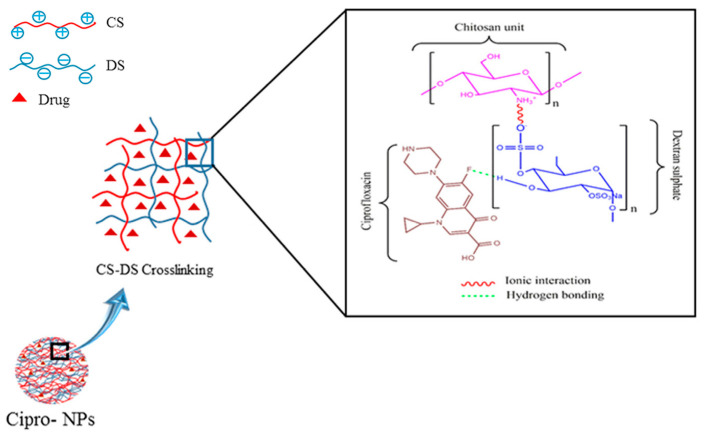
Chitosan (CS)/dextran sulfate (DS) and ciprofloxacin (Cipro) interactions and nanoparticle formation. Adapted from ref. [176]. Copyright 2017, with permission from Elsevier.

The combination of anionic dextran sulfate (DexS) and cationic CS, due to their contrasting charges, leads to the formation of mucoadhesive NPs showing sustained drug release, which is an interesting tool for overcoming the brief residence time of locally administrated APIs [12]. Chaiyasan et al. engineered CS and DexS to prepare NPs for the entrapment of lutein, which is a lipophilic antioxidant [174]. In order to avoid NPs aggregation during storage, poly(ethylene glycol) (PEG) 400 and 1-ethyl-3-(3-dimethylaminopropyl) carbodiimide (EDC), a commonly used stabilizing agent and a hydrophilic crosslinker respectively, were employed. The prepared cationic NPs showed a size of almost 400 nm, exhibited a high drug entrapment efficiency, and were mucoadhesive. It is notable that PEG and EDC improved the colloidal nanosuspension stability after storage, with the first providing a protecting role via steric hindrance. In a few words, lutein/CS/DexS NPs accomplished their purpose of ophthalmic external delivery, via electrostatic and hydrogen bonding between the synthesized NPs and the mucosal surface. Similarly, Manchanda et al. took advantage of the ionic interactions between the anionic DexS and cationic CS to prepare nanoparticulate systems containing acetazolamide, which is a model drug for the IOP decrease to glaucoma sufferers [175]. Six varied formulations were synthesized, changing factors such as the concentration of drug and the molecular weight of CS. NPs exhibited sizes in the range of 150 to 330 nm, while their smoothed surface was positively charged; the mucoadhesive nature of the prepared NPs could be a beneficial characteristic for the ophthalmic bioavailability of acetazolamide. Studies evidenced a proportional relationship between the CS/DexS ratio and the entrapment efficiency of acetazolamide. The irritation index of the prepared samples was estimated to be null, while in vivo ocular hypotensive tests revealed an interesting and sustained decrease of IOP for 5 h. A comparison between TPP and DexS as crosslinking agents for the preparation of CS NPs was further implemented [134]. The 12 formulations, prepared through ionotropic gelation, differed in the concentration of LMWCS and HMWCS, as well as the crosslinker type, and they were loaded with dorzolamide HCl. The optimal NPs were those prepared with the highest concentration of LMWCS, since they revealed a greater cationic surface (better affinity to the ocular mucosa) and improved dispersal stability. Despite differences in in vitro release and ex vivo permeation studies, the optimized NPs exhibited comparable in vivo results: a 5 h-lasting decrease in IOP. Similarly, Chavan et al. used CS/DexS NPs to design a ciprofloxacin delivery vehicle for the treatment of microbial infections [176]. FTIR and thermogravimetric analysis evidenced the conjugation between the drug and CS/DexS NPs via hydrogen bonds, as illustrated in Figure 7. Ciprofloxacin NPs showed enhanced antibacterial effects compared to the drug alone.

Silva et al. utilized hyaluronic acid (HA), a naturally existing anionic glycosaminoglycan with mucoadhesive and biocompatible properties that is able to interact with the ophthalmic epithelium [8]. CS was crosslinked with both TPP and HA to produce CS/TPP/HA NPs via ionotropic gelation. NPs were loaded with ceftazidime, which is an antibiotic for the treatment of ophthalmic infections, such as bacterial keratitis, and incorporated in eye-drop formulations containing HPMC. All the prepared NPs exhibited an average particle size of 350 nm, while their surface was positively charged, enhancing its mucoadhesive properties. The antimicrobial properties of ceftazidime were retained after its encapsulation into NPs, and the produced nanoparticulate system exhibited optimal characteristics for the local ocular antibiotic delivery. Silva et al. further encapsulated erythropoietin in CS/HA NPs [177]. Hyaluronic acids of different molecular weights and sources were investigated. The highest entrapment efficiency was observed for the highest molecular weight HA. Ex vivo permeation studies demonstrated a higher permeation through porcine conjunctiva, followed by sclera and cornea. Fabiano et al. prepared CS/HA-based NPs for the encapsulation of 5-fluorouracil [178]. Drug release was completed in less than three hours. These NPS were further incorporated in a CS gel to increase their therapeutic potential (*vide infra*).

The anionic chain of sulfobutylether-*β*-cyclodextrin (SBE-*β*-CD) has been investigated for the formation of nanoparticulate systems with CS. Indeed, SBE-*β*-CD, a water-soluble derivative of *β*-cyclodextrin, is non-toxic and thus extensively investigated by the pharmaceutical technology. In this context, Zhang et al. formulated SBE-*β*-CD/CS NPs, which were further loaded with naringenin [179]. Naringenin, a naturally existing flavanone, is employed for the treatment of age-related macular degeneration, as it provokes an effective boost of the ocular blood flow and shows an antioxidant character. The first step of the synthesis route was the complexation between SBE-*β*-CD and the drug, resulting in an increased water solubility of the latter. NPs were synthesized via the ionic gelation technique, while the physicochemical and thermal properties of the formulations were determined through size, ζ-potential, FTIR, and differential scanning calorimetry measurements. Naringenin/SBE-*β*-CD/CS NPs presented an average size of 446.4 ± 113 nm and a positively charged surface that is able to interact with cornea. In vivo studies indicated the improved performances and the harmless nature of the formulations, and thus their applicability for ophthalmic uses.

Sodium deoxycholate, the sodium salt of deoxycholic acid, is frequently used as a biological detergent to lyse cells and solubilize cellular components. In many cases, due to its surfactant properties, sodium deoxycholate is thought to enhance the APIs absorption in the interior of biological systems. A promising approach developed by Alqurshi et al. took advantage of the ionic interactions between CS and sodium deoxycholate as a counter-ion to formulate self-organizing NPs for the delivery of prednisolone acetate, which is a lipophilic drug applied in inflammatory ocular treatment [180]. Studies revealed the relation between the CS/sodium deoxycholate concentration and the size of the prepared NPs, while the addition of PVA during preparation contributed to obtaining particles with remarkable characteristics. Moreover, clinical trials on female guinea pigs exhibited a superior action of the prepared NPs regarding the anti-inflammatory activity, in contrast with the marketed prednisolone acetate gels.

Poloxamers or Pluronics^®^ are a class of water-soluble, non-ionic, triblock copolymers, composed of a central poly(propylene oxide) (PPO) block and two terminal poly(ethylene oxide) blocks [181]. A wide range of poloxamers is commercially available with varying block molecular weights. Poloxamers play an important role in drug delivery systems owing to their stabilizing and strongly mucoadhesive nature. The latter is based on the dual properties of poloxamers, possessing both lipophobic and lipophilic units resembling mucosa action [12]. Ahuja et al. employed a nanoprecipitation method to formulate CS/lysine/poloxamer-188 itraconazole-loaded NPs [182]. Precipitation was triggered by water addition due to pH alterations and the presence of a non-solvent. Ex vivo studies applied to isolated goat cornea revealed an increased itraconazole corneal uptake in the case of nanosuspensions compared to marketed suspensions, rendering them appropriate candidates for ocular delivery.

Natesan et al. prepared PEG-modified CS/TPP NPs, applying “traditional” ionic gelation but by adding PEG to the TPP solution [183]. PEG of different molecular weights and in different concentrations was used. According to the authors, PEG was bound to the CS NPs through hydrogen bonding and formed a semi-interpenetrating network. The NPs were loaded with resveratrol and quercetin, which are two natural antioxidants. An increase in PEG concentration resulted in bigger NPs but to the expense of drug loading. Ex vivo corneal permeation demonstrated an important enhancement in permeation compared to resveratrol, and in vivo studies showed a prolonged IOP lowering effect up to 8 h compared to timolol maleate—and, due to the synergistic effect of the two drugs, better performances than resveratrol alone.

Åhlén et al. embedded CS/PAA NPs in PVA contact lenses as a potential vehicle for lysozyme-triggered drug delivery [184]. CS/PAA NPs were prepared via a radical polymerization route in three different CS/PAA ratios. NPs obtained from 1:1 CS/PAA were the smallest with the lowest net charge. A 0.2 mΜ lysozyme concentration, similar to lysozyme concentration in tear fluid, was found to be adequate to degrade the polymeric NPs within 5 h, triggering drug release.

When combined with metal ions, CS forms non-toxic crosslinked structures that have demonstrated exceptional wound-healing properties. Indeed, this system can initiate the production of nitric oxide (NO), which is a biomolecule produced in many bioprocesses under wound-provoking conditions through different cellular types. In this context, Tellios et al. designed a CS/Cu/glucose composite to evaluate its wound-healing potential in human corneal and limbal epithelial injury [185]. Analysis methods revealed a fastened Cu–CS activity in human corneal epithelial wound closure after 3 days, in contrast to the human limbal epithelial injury effects. The latter was attributed to the overproduction of NO, leading to cell necrosis. The above-mentioned differences between the two cellular models are an aftereffect of different metabolic pathways and NO-indicating procedures.

#### 3.1.3. Nanoparticles from Modified Chitosan

Despite all its advantages, CS presents some limitations. One of them is its low solubility in neutral and alkaline pH. Chemical modification is a means to enhance its solubility as well as other properties such as mucoadhesion, bioavailability, biocompatibility, etc. and to broaden its spectrum of applications. Indeed, when CS NPs are prepared via ionic gelation, the positively charged amino groups react with negatively charged TPP groups. However, this reaction blocks most of the amino groups of CS and thus might reduce the mucoadhesive ability of CS. In an earlier study, the interactions between CS and mucin were evaluated by measuring the ζ-potential values: the higher the ζ-potential, the higher the mucoadhesive properties [186]. Mucin alone has a negative ζ-potential: −18.7 ± 0.6 mV [187], which is very close to the value of −19.4 mV (at pH 7) that was reported from Sogias et al. [54]. CS has a positive ζ-potential close to +46.7 ± 0.4 mV, which decreases progressively by increasing the amount of TPP used for NPs preparation (*vide supra*) [188]. As the number of available –NH_3_^+^ groups—which are able to ionically interact with the sialic acids of mucin—decrease, a reduced mucoadhesion is expected. Appropriate derivatives can attenuate this drawback. CS can be modified on either its hydroxyl functionality or its amine groups, the latter being more frequent, and modified CS derivatives have been used for NPs formulation with TPP (Table 3) or without.

**Scheme 1:**
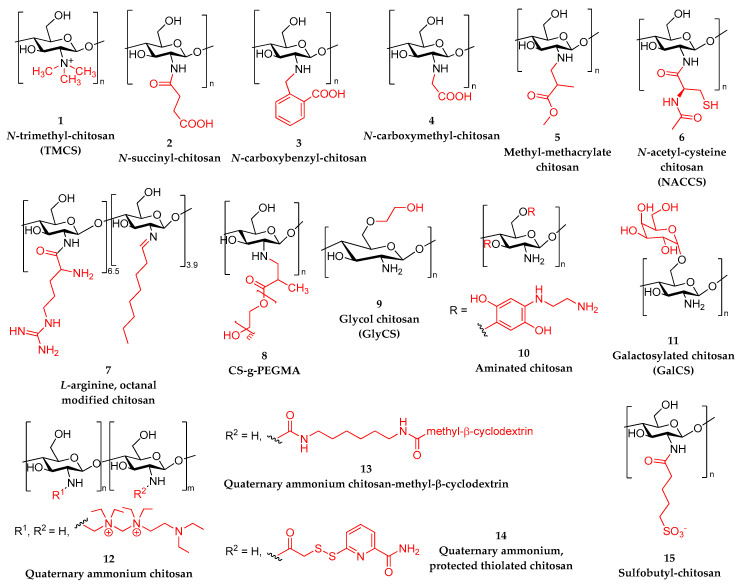
Modified repeating units of chitosan derivatives used for the preparation of nanoparticles.

*N*-trimethyl CS (TMCS), **1** in Scheme 1, where the amino groups have been quaternized by the introduction of methyl groups, is the simplest quaternized CS. The quaternazitaion of CS amino groups is an easy method to extend CS solubility over a wide range of pH. Asasutjarit et al. employed TMCS as a convenient carrier for diclofenac sodium, a non-steroidal anti-inflammatory drug, to enhance its ocular bioavailability [189]. TMCS was synthesized by reductive methylation and NPs were further prepared via ionic gelation with TPP as a crosslinking agent (TMCS/TPP weight ratio: 1:1, 2:1, 3:1, 3:2, 4:1). The optimized formulation (size 155 nm, PDI 0.2, ζ-potential 8.3 mV and entrapment efficiency 93.3%) was prepared with a TMCS/TPP/diclofenac sodium weight ratio of 3:1:1. According to the authors, FTIR evidenced weak H-bond interactions between diclofenac sodium and the polymeric matrix, apart from the expected electrostatic interactions. In vivo studies analyzed the presence of diclofenac sodium in rabbits’ aqueous humors after the instillation of the prepared formulation. The maximum concentration of diclofenac was comparable to the marketed eye drops, and after 12 h, the concentration was still within the range of the minimum effective concentration. Shinde et al. used TMCS to encapsulate flurbiprofen, which is an API that is regularly prescribed for the treatment of bacterial conjunctivitis, aiming at enhancing the residence time on the ocular surface, thus decreasing the frequency of the instillation and improving patient compliance [190]. The specificity of this work is that flurbiprofen was encapsulated as an inclusion complex with hydroxyl propyl-*β*-cyclodextrin to increase its water solubility. NPs were formulated with TPP and loaded with the flurbiprofen/cyclodextrin complexes. TMCS loaded NPs ranged from 200 to 800 nm and exhibited an increased mucoadhesion compared to CS NPs. Biphasic drug release was observed with a burst release followed by a sustained one, according to in vitro release studies.

Siafaka et al. reported the amino group modification of CS with succinic anhydride [191] and 2-carboxybenzaldehyde [192], **2** and **3** in Scheme 1, and they examined their performance as nanocarriers for the entrapment of timolol maleate [191]. NPs were prepared by ionic gelation with TPP. The size of the obtained NPs depended on the modified CS/TPP ratio, and to rationalize the differences between the two derivatives, the interactions with TPP were theoretically investigated applying calculations based on density functional theory. Timolol maleate was satisfactorily encapsulated in the NPs in an amorphous form, with a better encapsulation in the case of the carboxybenzaldehyde derivative, which was perhaps due to its higher hydrophobicity. The theoretical release data analysis indicated that the release of the active substance was a multistage process with drug diffusion being the leading release mechanism.

Ambhore et al. published a work on the combination of HPMC and *N*-carboxymethyl CS, **4** in Scheme 1, with two types of pluronics, namely Poloxamer 407 (P407) and Kolliphor-P188 [193]. Nanosuspensions were prepared via a solvent diffusion technique and loaded with sparfloxacin, which is an antibiotic that is used against bacterial conjunctivitis. Due to steric hindrance effects, the two surfactants prevented any aggregation of the prepared NPs and thus enhanced the formulations’ stability. The optimal formulation was the one based on *N*-carboxymethyl CS nanosuspension in combination with pluronics, revealing 94 ± 2% entrapment efficiency, a zero irritation index, and sustainable sparfloxacin release.

Jaiswal et al. reported a green synthesis of methylmethacrylate CS, **5** in Scheme 1, via a Michael addition and the subsequent preparation of curcumin-loaded NPs using ionic gelation with TPP [194]. Different CS/methyl methacrylate mol ratios were investigated: 1:2.5, 1:5, and 1:10. The optimized particles (small size (200 nm in average), low PDI (3.2), high drug entrapment (68%)) were obtained from the derivative synthesized with the lowest amount of methyl methacrylate. In vitro release of the drug was studied at two different pH values (5.0 and 7.4), and a higher release was observed in acidic medium.

Rajawat et al. reported a thiolated CS derivative, **6** in Scheme 1, as a microcarrier for the ocular delivery of acyclovir [195]. *N*-Acetylcysteine was covalently bonded to CS via a carbodiimide-mediated coupling reaction. The produced modified CS bore 492 ± 30 mmol of thiol groups/g of polymer and showed higher mucoadhesion and enhanced thermal stability comparatively to neat CS. Acyclovir-loaded microspheres were prepared via emulsification followed by crosslinking. In vitro release of acyclovir in simulated tear fluid (STF) took place in two phases: a burst release followed by a sustained release for 12 h. No symptoms of ocular toxicity were observed in vivo, confirming the potential of *N*-acetylcysteine CS (NACCS) microspheres for ocular administration.

Mauro et al. presented an innovative amphiphilic CS derivative with L-arginine and octyl groups, **7** in Scheme 1, as a polymer carrier for sorafenib tosylate [196]. More specifically, CS oligosaccharide amino groups were initially modified by L-arginine via a carbodiimide-mediated coupling reaction and then with octanal to confer an amphiphilic behavior. The copolymer was utilized for the formulation of microparticles applying a coacervation technique. The resulting microparticles consisted of an arginine-decorated hydrophilic shell and inner hydrophobic domains, which were expected to promote the entrapment of hydrophobic drugs (>10% *w/w*). In vitro release studies evidenced a sustained release over 12 h, but more remarkably with no burst effect. In addition, it is notable that in vitro transcorneal studies showed a significantly increased permeation, which was attributed to the presence of arginine residues with permeation-enhancing properties on their outer shell.

**Figure 8:**
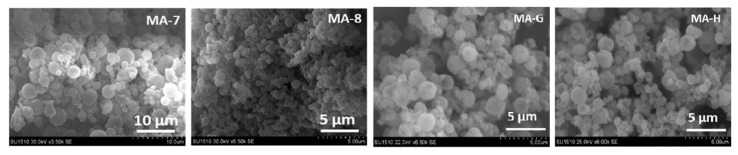
Scanning electron microscopy images of nanoparticles crosslinked with sodium tripolyphosphate (MA-7 and MA-8) or sodium sulfate (MA-G and MA-H). Reprinted from ref. [197]. Copyright 2019, with permission from Elsevier.

Savin et al. reported a novel type of polymer nanocarriers, based on CS modified with poly(ethylene glycol) methacrylate, **8** in Scheme 1 [197]. Although PEG-grafted CS derivatives have been reported, in this study, CS-grafted poly(ethylene glycol) methacrylate (CS-*g*-PEGMA) was synthesized using a Michael addition, instead of radical grafting. Bevacizumab-loaded micro- and nanoparticles were prepared via double crosslinking, ionic (TPP or Na_2_SO_4_) followed by covalent (glutaraldehyde) in reverse emulsion (water in oil). NPs sizes depended on the concentration of the polymer and were measured between 200–900 nm and 1000–1500 nm depending on the crosslinker, TPP or Na_2_SO_4_, respectively. As observed in Figure 8, the latter produced bigger particles due to a lower crosslinking density. A drug release study revealed a slow sustained release with a small burst effect. Data were fitted on a Korsmeyer–Peppas model, and drug transport was mainly controlled by Fickian diffusion.

The aforementioned modifications concern the amino groups of CS; however, CS bears two free hydroxyl groups, which have also been used to introduce modifications, especially the primary hydroxyl group, as it is more reactive. Glycol CS (GlyCS), **9** in Scheme 1, is an appealing water-soluble CS derivative. Yu et al. investigated cerium oxide/GlyCS NPs as a treatment for dry eye disease [198]. GlyCS/cerium oxide particles were formulated in the presence of ammonium hydroxide. Spectroscopic analysis revealed that cerium oxide was successfully encapsulated into modified CS NPs. The NPs did not show any cytotoxic effect and resulted in improvements in dry eye disease models, while preserving the integrity of epithelium. A series of dexamethasone–GlyCS conjugates (Dex-GlyCS) were synthesized by Yu et al. as dexamethasone carriers aiming at increasing precorneal retention and corneal permeability [199]. Succinated dexamethasone was initially synthesized, and this derivative was further coupled to the amino groups of GlyCS via the carboxylic end group of the succinic spacer. Dex-GlyCS self-assembled spontaneously into spherical NPs ranging from 270 to 290 nm, with a positive ζ-potential (roughly +15 mV). Dexamethasone release was evaluated in vitro in phosphate-buffered saline (PBS, pH = 7.4) and showed a progressive release for 8 h, followed by a 48-h plateau. Finally, an in vivo distribution test indicated a relatively long-term residence on the corneal surface.

**Figure 9:**
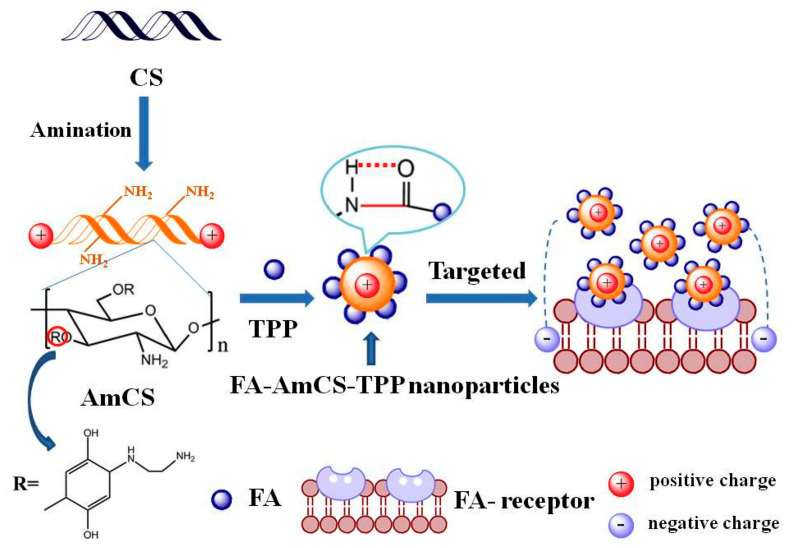
Preparation and action mechanism of folic acid (FA)-modified aminated chitosan (CS)/sodium tripolyphosphate (TPP) nanoparticles (FA-AmCS-TPP) [200].

In an attempt to increase CS mucoadhesive properties, Sun et al. reported an aminated derivative of CS (AmCS), **10** in Scheme 1, which was employed to prepare folic acid-modified AmCS/TPP NPs for the encapsulation of curcumin, in order to enhance its physicochemical and biological properties as an antitumor drug, as shown in Figure 9 [200]. The small size of the produced particles was associated to their positive ζ-potential, which was advantageous regarding their use as carriers for antitumor API. The factors affecting curcumin encapsulation were optimized, and the subsequent in vitro release study demonstrated a biphasic release, with a cumulative release of 56.2% at 48 h. In addition, results concerning cytotoxicity indicated that the formed NPs had a high targeting capacity for tumor cells, supporting the hypothesis of improved polymer nanocomplexes for the treatment of cancer.

Zhao et al. introduced galactosylated CS (GalCS), **11** in Scheme 1, NPs in ocular delivery, for the delivery of timolol maleate [201]. NPs were prepared via ionic crosslinking, and the experimental conditions were optimized using a four-factor and three-level Box–Behnken design. The independent variables were GalCS and TPP concentrations, and GalCS/TPP and timolol maleate/GalCS mass ratios, while the dependent variables were the particle size, the encapsulation efficiency, and the loading capacity. The optimal NPs demonstrated a particle size of 223.3 ± 6.8 nm with an encapsulation efficiency of 39% and an active substance loading of 18%. In addition, the in vitro release study revealed a sustained release over 8 h; in comparison, the commercial eye drops released 89% of timolol maleate within 2 h. More importantly, the in vivo study demonstrated a much-improved performance of the GalCS NPs compared to both commercial eye drop formulation and CS NPs.

Piras et al. developed a novel water-soluble CS derivative that is able to form complexes with dexamethasone, which is a lipophilic API [202]. CS was initially quaternized with 2-diethylaminoethyl chloride via amino group modification, **12** in Scheme 1, followed by the grafting of methyl-*β*-cyclodextrin on the resulting modified derivative, using hexamethylene diisocyanate as a linker, **13** in Scheme 1. The final product was highly soluble in water, with a 10-atom-long spacer between the CS backbone and the cyclodextrin, which was capable of encapsulating dexamethasone. Concerning the in vitro cytotoxicity study, it was found that the cell viability was maintained above 80%, implying that the resulting conjugate could be used as a solubilizing excipient for ophthalmic formulations. Thanks to the strong interactions between the active substance and the polymer matrix, the prepared derivative appeared promising for future processing into 3D solid forms, such as controlled drug delivery systems, films, or medical devices. In line with this work, Fabiano et al. explored three different CS derivatives for the formation of NPs and the ocular delivery of 5-fluorouracil: quaternized CS derivative (positively charged), **12** in Scheme 1, a derivative bearing a protected thiol group, **14** in Scheme 1, and one bearing a sulfobutyl group (negatively charged), **15** in Scheme 1 [203]. NPs were prepared by self-assembly upon the addition of HA. All three derivatives yielded NPs with similar sizes (290–390 nm) and entrapment efficiency (15–18%) but, as expected, different ζ-potentials: positive for derivatives **12** and **14** and negative for derivative **15**. Due to the intrinsic mucoadhesivity of CS, derivative **15** showed some mucoadhesivity; however, it was much weaker than derivatives **12** and **14**. These NPs were further incorporated into a thermosensitive hydrogel.

#### 3.1.4. Nanoparticles by Spray Drying

Spray drying is an automated one-step process for the production of microparticles. It consists of spraying a liquid under a hot air current to produce a powder. Its simplicity and low-cost are only two advantages, among others. However, in contrast to ionic gelation that generally yields nanoparticles, in most cases, the particles obtained by spray drying are in the micrometric range. Addo et al. reported the synthesis of CS/albumin microparticles to entrap an extensively used ophthalmic drug: atropine sulfate [204]. The microspheres’ formulations were shown to be optimal for ocular delivery physicochemical properties and enhanced effects on mydriasis (a pupil’s dilation disorder) in rabbit models compared to commercial solutions. Using the same technique, Zhou et al. prepared double crosslinked microspheres for the delivery of an antibacterial agent, levofloxacin. More specifically, CS/TPP gel was initially spray dried to obtain microparticles, and the latter were additionally crosslinked using glutaraldehyde in a following solidification stage [205]. This innovative extra crosslinking step resulted in an enhanced stability, since it offered controllable water-swelling capability. The formulated particles revealed a rounded shape with sizes of 1–2.5 μm, while their cationic surface presented folds. The microspheres exhibited satisfying entrapment efficiency, whereas in vitro tests displayed a crosslinking-dependent profile and a sustained levofloxacin release within 1 day. Ornidazole, an antimicrobial agent, was also entrapped in CS NPs by spray drying to exploit the cationic and mucous-attractive nature of CS [206]. Similarly, Costa et al. utilized the same technique to formulate pilocarpine-loaded CS particles in the microscale for intraocular delivery [207]. Strong interactions between CS and pilocarpine resulted in sustained release, preventing the initial burst release often observed. Additionally, high porcine sclera permeability was observed for the formulated microparticles.

### 3.2. Micelles

Micelles are spontaneously formulated nanostructures composed of amphiphilic polymers. The hydrophobic part of the polymer self-assembles into a stiff core, while the hydrophilic part is oriented to the outer surface of the micelle between the hydrophobic core and the aqueous media. Formulated micelles are of small size, ranging between 5 and 30 nm [208], which is advantageous when subcellular localization is sought. They are frequently utilized in various drug delivery systems for the inclusion of poorly aqueous soluble compounds in the core of the micelles, with high drug-loading efficacy [209,210]. Their final structure is stable with enhanced tissue permeability [211], while they can easily respond to environmental changes—namely pH, temperature, or ionic strength. However, this susceptibility to environmental variations is the main reason for the precipitation of the encapsulated drug during storage [209]. Neat CS is not capable of forming micelles by itself; nevertheless, modified CS has been used for the formulation of polymeric micelles aiming at the ocular release of various pharmaceutical compounds.

**Scheme 2:**
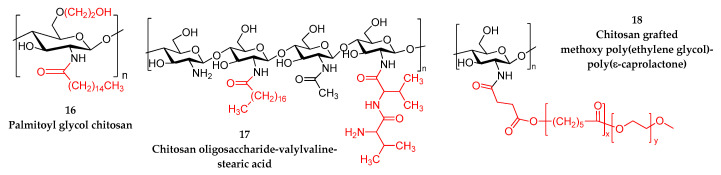
Modified repeating units of chitosan derivatives used for the preparation of micelles.

Bonferoni et al. prepared palmitoyl glycol CS, **16** in Scheme 2, with three different degrees of substitution and compared the prepared micelles with pluronic (F127) micelles, with the intention of encapsulating cyclosporine A [212]. The results revealed that enhancing the degree of substitution resulted in increasingly hydrophobic derivatives, while regarding the size of the prepared micelles, they were around 1 μm for all derivatives, owing to aggregation. Interestingly, the formation of micelles with the most hydrophobic derivative led to a stiff core, but a low loading percentage of cyclosporine. In contrast, the least substituted derivative exhibited the optimal drug-loading capacity due to a strong association with cyclosporine. Furthermore, the micelles of the most hydrophilic derivative were submitted to interaction with various biological substrates through in vivo and ex vivo studies, and enhanced drug penetration inside the cornea was observed.

CS oligosaccharide-valylvaline-stearic acid, **17** in Scheme 2, an alternative CS derivative, was synthesized by Xu et al. for the prepareration of dexamethasone-containing nanomicelles [213]. The purpose was the topical ocular delivery of dexamethasone for the treatment of macular edema. The average size of the prepared nanomicelles was 30 nm, whereas their ζ-potential was +30 mV. The positive ζ-potential favored interactions between micelles and the ocular anionic surface. In vitro drug release in STF revealed that the nanomicelles could ensure a sustained release of dexamethasone, avoiding the burst effect. The prepared nanostructures were found to be non-toxic in in vitro cellular studies, while the permeability of dexamethasone was enhanced. Moreover, nanomicelles, due to the presence of the polysaccharide, were able to reach the posterior segment of the eye via the conjuctiva route.

**Figure 10:**
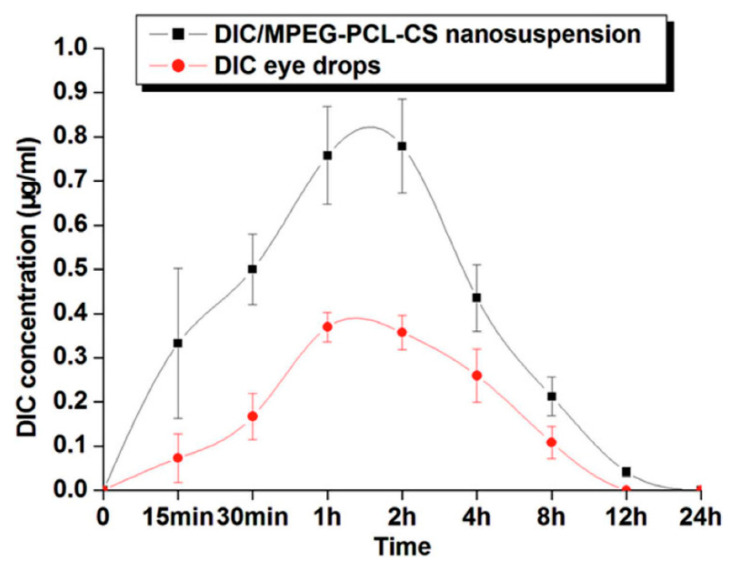
Diclofenac (DIC) concentration profiles in rabbit aqueous humor after instillation of 50 μL of either a 0.1% (mass fraction) DIC commercial eye drops or 0.1% (mass fraction) DIC/chitosan grafted methoxy poly(ethylene glycol)-poly(ε-caprolactone) (DIC/MPEG-PCL-CS) nanosuspension. Reprinted from ref. [214]. Copyright 2015, Springer Nature.

The presence of CS was responsible as well for the improved performances of micelles containing diclofenac [214]. Shi et al. prepared a block polymer composed of CS and methoxy poly(ethylene glycol)-poly(ε-caprolactone), **18** in Scheme 2, which spontaneously self-assembled in water, forming micelles. The resulting cationic suspension was stable, the mean size of the prepared micelles was 105 nm, while the ζ-potential was +8 mV, owing to the presence of CS. In vitro release studies showed a two-step release behavior: an initial burst release followed by a sustained release. In vivo tests in rabbits indicated enhanced penetration, precorneal retention, and improved bioavaliability of diclofenac (Figure 10), which were attributed to the positive charge of the micelles that interacted with the negatively charges of the ocular mucin.

As mentioned earlier, Hafner et al. compared two nanosystems for the ocular delivery of melatonin aiming to control IOP [166]. More specifically, they prepared CS/L NPs as well as CS/pluronic (F127) micelles. Concerning the size and ζ-potential of the prepared nanostrucrures, the NPs were larger in size and had a positive surface charge. On the contrary, micelles had a tremendously small size (20.7 nm) in comparison to the previous micelles, and a neutral surface charge. CS presence affected in vitro release from the two delivery systems differently. Concerning the NPs, the presence of the polysaccharide resulted in a decrease of the release of melatonin, whereas the presence of CS resulted in an enhanced and faster release from the micelles. Finally, regarding the permeation ability, micelles revealed an enhanced permeability due to the presence of F127, while the presence of CS led to a diminution of the melatonin permeability due to electrostatic interactions.

### 3.3. Lipid Nanoparticles and Liposomes

Lipid NPs aim to improve the bioavailability as well as the release behavior of poorly water-soluble active compounds. They consist of a lipophilic core that is stabilized by various surfactants in an aqueous environment. Lipid NPs are considered stable systems with constant physicochemical properties. Moreover, they can be administered by all routes of administration. However, they demonstrate inadequate drug-loading efficacy, and as micelles, they are susceptible to the expulsion of the encapsulated compound during storage [215]. In contrast to lipid NPs, liposomes are composed of an aqueous core that is stabilized by a surfactant bilayer and can be utilized for the inclusion of both lipophilic and hydrophilic compounds. The structure of liposomes resembles the structure of living cells and consequently, their biocompatibility is exceptional. Furthermore, they show good biodegradability and low inner toxicity. Compared to various lipid carriers, liposomes seem to have improved drug-loading capacity and an enhanced protection of the various encapsulated drugs against the extreme conditions of the gastrointestinal environment [216]. This feature renders liposomes promising candidates for the entrapment of various active compounds aiming not only at ocular treatment but also at the treatment of various cancer types [211]. Nevertheless, in contrast to lipid nanoparticles, liposomes’ physicochemical properties are questionable. As a polysaccharide, CS is utilized in the preparation of both lipid NPs and liposomes.

Zhao et al. prepared nanoliposomes with TMCS, **1** in Scheme 1, aiming at the topical delivery of pilocarpine hydrochloride for the treatment of glaucoma [217]. In vitro drug release studies in phosphate buffer indicated a biphasic pattern, an initial burst release, followed by a sustained release. The positive surface charge of the liposomes, which is attributed to the presence of CS, resulted in an increase in viscosity of the media where in vitro release took place, which resulted in the continuous release of the drug. In vivo test in rabbits showed a non-irritant behavior and consequently, the prepared liposomes ensured a sustained release of the compound.

Olaminosomes containing CS hydrochloride (CS HCl) were prepared by Abd-Elsalam et al. via thin film hydration [218]. The purpose was the enhancement of the bioavaliability and the control of the release of agomelatine, which is a water-insoluble drug for the treatment of ocular hypertension. CS HCl was utilized in order to provide mucoadhesion to the olaminosomes. In vitro drug release studies in simulated body fluid revealed that the presence of CS resulted in a sustained and controlled release of the drug, while increasing the concentration of CS delayed the release of the drug from the olaminosomes. In vivo tests with white rabbits evidenced that the presence of CS in the olaminosomes led to improved ocular retention time and consequently highest decrease of ocular hypertention. In vivo histopathological studies proved that they were safe for in vivo applications.

Apart from liposomes, many groups have worked on the preparation of various nanostructured lipid carriers (NLC) containing CS. Liu et al. prepared cationic NLC applying a film-ultrasonic method, aiming at enhancing the ophthalmic bioavaliability of curcumin [219]. It is a water-insoluble compound; consequently, an amphiphilic derivative of CS, octadecyl-quaternized carboxymethyl CS, was utilized for the preparation of the cationic particles. The erosion and degradation of the lipid carrier ensured a sustained release of curcumin. The positive surface of the prepared particles was due to CS and induced interactions with the negative ocular mucosa, leading to an improved retention capacity of the NLC on the eye. In vivo studies in white rabbits revealed no toxicity or irritation of the ocular tissue. Octadecyl-quaternized carboxymethyl CS was also employed for tetrandrine ocular delivery, in order to improve the low aqueous solubility, the poor pre-ocular residence time, as well as the poor bioavaliability [220]. For this purpose, liquid crystalline NPs were formed by amphiphilic lipids in an aqueous environment. Their advantage compared to other drug delivery systems is their high encapsulation efficacy as well as their biphasic in vitro release: an initial burst release that is followed by a sustained release of tetrandrine. In vitro corneal permeation as well as pre-ocular retention time were indeed ameliorated due to the positively charged modified CS, which is capable of interacting with the ocular mucosa, while in vivo tests in white rabbits revealed increased transcorneal permeation of the drug.

### 3.4. Combined Drug Delivery Systems

Nanocarriers have an unquestionable potential as drug carriers for prolonged drug administration. However, drug release or targeting can be difficult to control. Hence, NPs are regularly combined with other delivery vehicles: a coating or bigger particle, a gel, a lens, or an ocular insert. Combined delivery systems synergistically associate advantages of both carriers and contribute to an even higher therapeutic effect. For example, a coating can offer targeting properties, while a gel or a lens can offer a more prolonged ocular residence time. In the next paragraphs, we will report interesting combined systems where CS NPs have been incorporated to other delivery systems.

For example, Khan et al. reported the manufacture of a mucoadhesive drug delivery system based on an in situ gel-forming nanosuspension of tobramycin sulfate [162]. Specifically, tobramycin sulfate-loaded CS microparticles, obtained by the emulsion/ionic gelation method, were distributed in a thermoresponsive in situ gel from poloxamer 407 (P407). All the prepared samples exhibited a controlled tobramycin sulfate release, thus allowing reduced dosing frequency during ophthalmic bacterial infections. Moreover, the gel did not cause any visual disturbances. Fabiano et al. incorporated 5-fluorouracil-loaded CS-based NPs in a CS/quaternary ammonium CS hydrogel [203]. Three different derivatives were studied, **12**, **14,** and **15** in Scheme 1, producing NPs with different ζ-potential. In in vivo tests, the hydrogels containing NPs prepared with the first and third CS derivatives showed a more prolonged presence of 5-fluorouracil in aqueous humors. The first one was due to increased mucoadhesion owing to the positive charges of the gel, while the third one was due to the formation of ionic interactions between CS (positively charged) and sulfobutyl CS NPs (negatively charged) that delayed drug diffusion.

Ameeduzzafar et al. incorporated levofloxacin-loaded CS NPs in a HPMC/ALG gel [135]. The formulation retained the antimicrobial properties of levofloxacin, and a significantly prolonged activity was detected in vitro in comparison to levofloxacin solution. Similarly, Ahdyani et al. incorporated CS/ALG NPs containing timolol maleate into a HPMC gel and observed a 24 h drug release [170]. Cui et al. also employed a HPMC gel for the delivery of bovine serum albumin encapsulated in CS/ALG core/shell NPs [221]. NPs were prepared using a nanoemulsion method and the two polymers were assembled in a polyelectrolyte compound due to strong electrostatic interactions. A significantly delayed release was observed with a cumulative release of 78% over 9 days.

**Figure 11:**
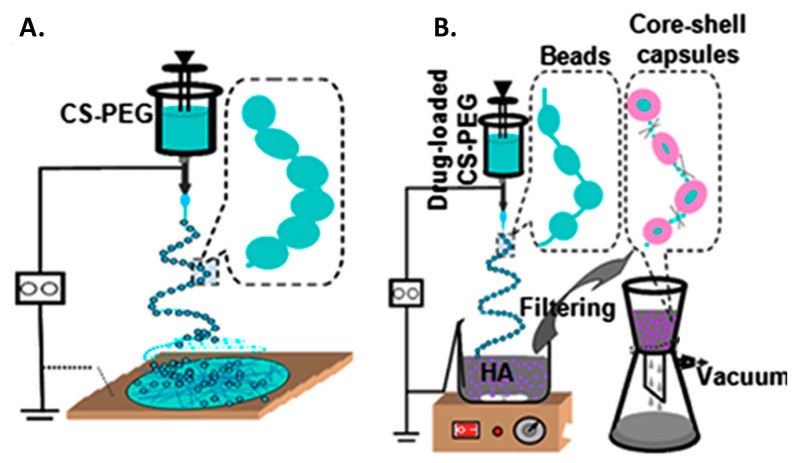
Schematic procedure of the preparation of core-shell microcapsules. (**A**) Chitosan-poly(ethylene glycol) (CS-PEG) composite bead-rich fibers collected by metal plate. (**B**) Core–shell structured microcapsules collected by 0.6% hyaluronic acid (HA) hydrosol via the in situ electrospinning technique. Reprinted from ref. [222]. Copyright 2017, Springer Nature.

Kalam coated dexamethasone-loaded CS NPs with HA to improve their cellular targeting and mucoadhesive properties [143,161]. HA was simply adsorbed on CS NPs due to their opposite charges. As a result of coating NPs, their size increased and ζ-potential decreased. The coated NPs exhibited a more sustained release and a better retention. Compared to a solution of free dexamethasone, a higher and more sustained permeation was observed. Xie et al. prepared CS/HA core–shell NPs by electrospinning a CS/PEG solution and collecting it directly in a HA sol, as shown in Figure 11 [222]. As the bead-rich fibers were collected in the stirred HA solution, the fibers broke, affording CS microcapsules coated with HA, due to ionic interactions. Microcapsules were loaded with ofloxacin, and the HA solution was crosslinked with adipic dihydrazide and EDC to form a gel with better mechanical properties. Ofloxacin release was controlled, with a low burst release and a sustained release over 21 days. The HA hydrogel was viscous enough to be injected and was intended to be used as resorbable punctal plugs for the treatment of dry eye disease or as a drug delivery vehicle.

**Figure 12:**
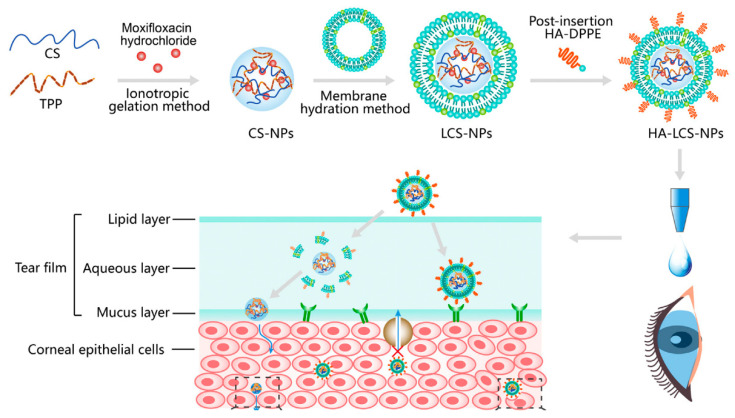
Schematic preparation of core–shell lipid–polymer hybrid NPs and illustration of the in vivo delivery and penetration behavior of drug-loaded hyaluronic acid/lipid-coated chitosan nanoparticles (HA-LCS-NPs). Reprinted from ref. [155]. Copyright 2018, with permission from Elsevier B.V.

Liu et al. designed core–shell lipid–polymer hybrid NPs: moxifloxacin hydrochloride-loaded CS NPs, surrounded by a phospholipid layer, bearing HA residues for a more efficient targeting (Figure 12) [155]. CS NPs were prepared by ionic gelation with TPP, and the lipidic bilayer was set by hydrating a dried lipid film by a NP suspension. The hybrid NPs exhibited a remarkable sustained release of moxifloxacin HCl. In an in vitro corneal permeation test, they performed three times better than a commercial moxifloxacin HCl preparation; finally, they improved the pre-corneal retention time. Overall, the bioavailability of moxifloxacin was significantly enhanced.

PLGA is a well-established candidate for the preparation of NPs due to its biodegradability and FDA approval. However, PLGA as a vehicle suffers from a variety of downsides, namely weak protein stability, loading efficiency, and abrupt release profile. An attractive method to bypass those drawbacks is described by Elsaid et al., who formulated a ‘system-within-system’ matrix for the treatment of age-related macular degeneration [158]. Specifically, the aforementioned system comprised of PLGA microparticles, which contained CS-based NPs, for ranibizumab delivery to vitreous. The incorporation of NPs in the PLGA microparticles structure took place via a modified water/oil/water double emulsion method, in which several types of CS NPs were utilized: CS/TPP or CS/TPP-HA, NACCS, **6** in Scheme 1, and NACCS/TPP. NPs as well as microparticles including NPs were analyzed thoroughly to investigate ranibizumab–NP interactions and evaluate the drug release profile. The results revealed that the PLGA microparticles including the NACCS NPs showed the highest ranibizumab-loading percentage and enhanced release, reinforcing the hypothesis of strong disulfide bonds between NACCS and cysteine residues appearing into the drug structure. In addition, CS/TPP-HA NPs revealed anti-angiogenic activity due to HA, which was a beneficial characteristic that was unfortunately countered by the rapid rate of degradation of the corresponding PLGA microparticles.

Finally, CS NPs have also been introduced into ocular lenses and inserts for a prolonged retention on the ocular surface. In their work, Han et al. formulated surperficially modified contact lenses with a hydrophilic exterior, intending to decrease the occurrence of posterior capsular opacification: a basic complication faced after cataract surgery [147,148]. For this purpose, CS NPs loaded with doxorubicin were prepared via ionic gelation. Afterwards, the cationic CS NPs were assembled with anionic heparin onto the intraocular lens, resulting in a multilayer polyelectrolyte complex. In vitro studies revealed a decreased cell adhesion after the surface covering with antiproliferative drug-loaded multifunctional system, whereas the same outcome occurred for the cell migration and proliferation properties. The in vivo biocompatibility of the multilayered superficially modified ionic lenses was enhanced compared to neat or hydrophilically surface-modified lenses.

Another study based on the merits of CS/TPP NPs was planned by Behl et al., who synthesized NPs laden with an anti-inflammatory agent, namely dexamethasone sodium phosphate, administrated in numerous ophthalmic diseases [151]. Drug-loaded particles were further incorporated in the structure of poly(2-hydroxyethylmethacrylate) (PHEMA) contact lenses. The designed lenses demonstrated a sufficiently transparent nature. An initial dexamethasone burst release was observed, followed by sustained release reaching 55.7% within 22 days. This work highlights the potential of drug-reservoir contact lenses for the replacement of marketed eye drops. Åhlén et al. immobilized CS/PAA NPs in PVA contact lenses [184]. Neat PVA lenses and PVA lenses reinforced with cellulose nanocrystals were studied and the lysozyme-triggered release of fluorescently-labeled NPs was examined. Mixing cellulose nanocrystals with CS/PAA, prior to their incorporation in PVA lenses, yielded a gel due to ionic interactions, which prevented the NPs from leaching out of the lenses.

Badiee et al. inserted bevacizumab-loaded CS NPs in an HA/zinc sulfate implant [132]. The NPs were uniformly dispersed in the implant. The system exhibited a negligible initial burst release followed by a slow release of bevacizumab extending over two months. Similarly, Morsi et al. introduced a ketorolac tromethamine CS nanodispersion in Eudragit/HPMC/PEG 400 films, which were intended as ocular inserts for the treatment of post-operative ocular inflammation [138]. Drug release was significantly slower when NPs were incorporated in the film. In vivo tests demonstrated that overall, the NPs/film delivery system exhibited a more effective suppression of inflammatory signs.

## 4. Hydrogels

Hydrogels are three-dimensional polymer networks, generated by physical (non-covalent) or chemical (covalent) crosslinking. They are hydrophilic, yet insoluble in water, as a result of their crosslinked structure. Hydrogels can swell to a high extent by absorbing large amounts of water, becoming soft viscoelastic gels that can be used for ocular controlled drug release [223,224]. Water absorption is a result of their hydrophilicity. Drug release from hydrogels occurs mainly by swelling, erosion, diffusion, or a combination of those [225,226]. Viscoelastic studies showed that the gel strength between polymers and a mucus surface can be explained by the formation of both physical molecular entanglements and secondary chemical bonds such as hydrogen bonds [227].

In ocular delivery, hydrogels present advantages compared to eye drops, as their higher viscosity allows for a longer residence time on the ocular surface and thus a higher therapeutic effect. However, gels have been linked to blurred vision and other discomforts. In situ gelling systems are systems that undergo a sol–gel phase transition in response to external stimuli (temperature, pH, ionic strength…). In situ gelling formulations have garnered important interest as, additionally to the advantages presented by hydrogels, they can be instilled in the eye as a solution, thus facilitating administration. Indeed, in situ gelling formulations undergo phase transition in the ocular cul de sac to form viscoelastic gels. Most of these gels exhibit a pseudoplastic or non-Newtonian behavior. In other words, their viscosity decreases when the shear stress increases. This is particularly important for ocular delivery, as it prevents creating discomfort to the patient during blinking. The ocular shear rate is low at rest and increases importantly during blinking; a shear-thinning behavior ensures a low viscosity during blinking, thus avoiding patient discomfort.

Numerous polymers are extensively used for hydrogel preparation in pharmaceutical technology, as well as for ocular drug release, including natural polymers such as polysaccharides, gellan, ALG, HPMC, carrageenans, CS, and their derivatives, as well as some synthetic polymers, such as poloxamers and polyacrylates [41,228,229]. Blends between them can be used also as hydrogels for drug delivery in ophthalmic applications [230].

### 4.1. Chitosan Hydrogels

Proniosomes are vesicular systems bearing a bilayer made from non-ionic surfactants, such as cholesterol or sorbitan esters. They are generally more stable and easier to handle than liposomes or other vesicular formulations. Fouda et al. investigated the ocular delivery of dorzolamide HCl for the treatment of elevated IOP in glaucoma patients [231]. The dorzolamide HCl proniosomal gel was prepared with L-α-lecithin, cholesterol, and Span 40 (sorbitan monopalmitate) and the optimized formulation, in terms of dorzolamide HCl entrapment and particle size, was incorporated in a CS gel (0.2%). In vivo studies were carried out in comparison to marketed dorzolamide HCl eye drops. The proniosomal CS gel showed a fast onset of action, followed by a prolonged lowering of IOP, reaching its highest value (45% decrease) 6 h after administration and still showing some therapeutic effect (20% decrease of IOP) after 8 h. This was attributed to the CS gel, which allowed for a longer contact time at the ocular surface and the ability of niosomes to act as penetration enhancers.

Pure CS is not often used in gels on its own. Despite its pH-sensitive behavior, CS solutions generally exhibit low viscosities. However, formulations where CS chains are crosslinked via ionic interactions can result, depending on conditions, in the formation of hydrogels. For example, Abdel-Rashid et al. developed a formulation combining nanovesicles with a CS hydrogel to improve the ocular delivery of acetazolamide [232]. Acetazolamide was loaded into sorbitan monostearate/sodium deoxycholate nanovesicles, which were further incorporated in a CS hydrogel prepared by ionotropic gelation with TPP. The nanogel obtained with the 1.5% *w/w* concentration exhibited the highest viscosity and mucoadhesion time. In vitro drug release study indicated a progressive release via a diffusion-controlled mechanism. According to in vivo studies, IOP was lowered to normal levels within 45 min after the administration of the formulation and showed a prolonged therapeutic effect for 24 h. The oral administration of a commercially available acetazolamide tablet led to a decrease in IOP 1.5 h after administration and only for 5 h. In their work, Mohammed et al. used another phosphate: β-glycerolphosphate (β-GP) [233]. They developed a CS-based in situ gelling antimicrobial formulation, containing moxifloxacin or gentamicin (antibiotics) [233], where thermogelling properties were induced by the addition of β-GP [234]. An aqueous solution of β-GP was added dropwise to a cooled solution of CS in aqueous HCl. The obtained system was a viscous liquid below 29 °C, while sol–gel transition took place from 29 to 37 C. The antimicrobial activity of the gels was studied. It was found that the bare CS hydrogel had some antimicrobial activity on its own against *S. aureus*; however, only the antibiotic-loaded formulations could achieve complete growth inhibition. Sustained release from the antibiotic-loaded gels was observed over 4 h.

### 4.2. Chitosan Blends

The occurrences of “pure” CS gel formulations are scarce. More often than not, CS is used in combination with other polymers in order to obtain formulations with improved properties. CS forms readily hydrogels with neutral or negatively charged macromolecules, such as poloxamers (Table 4), proteins (gelatin, collagen) (Table 5), other polysaccharides (HA, ALG, xanthan, DexS) (Table 6), or synthetic polyanions such as PAA.

#### 4.2.1. Non-Ionic Polymers

Poloxamers can form thermoreversible gels in aqueous solutions, generally around 37 °C, and they have attracted growing interest as carriers for bioactive compounds and other biomedical applications [181]. Nevertheless, the poor mechanical properties are a major drawback of pure poloxamer gels. Thus, blends with other polymers, CS amongst others, are often used to modulate their properties.

Irimia et al. have reported in situ gelling P407/CS formulations for the ocular delivery of bupivacaine hydrochloride, which is used as a topical anesthetic [235]. A series of P407/CS dispersions with various poloxamer concentrations were prepared by the “cold method”. The gelling temperature of these formulations ranged from 25 to 37 °C, decreasing with increasing poloxamer concentration. The gelling temperatures increased upon dilution with STF. After dilution, the system prepared with 15% poloxamer concentration exhibited a phase transition at 35 °C, which is appropriate for ocular in situ gelling systems. The viscosity of the formulations was proportional to poloxamer concentration. When in vitro release of bupivacaine hydrochloride was studied, it was observed that increasing the P407 concentration resulted in a lower diffusion coefficient and thus a slower release rate.

Krtalic et al. studied mixtures of two different poloxamers (P407 and P188) with CS prepared by a modified “cold method” [236]. The rheological properties versus the concentrations of the three polymers were studied, and mathematical equations were established to describe the dependence of the gelation temperature (*T_gel_*), complex viscosity, and storage modulus on the composition of the hydrogel. Regarding *T_gel_*, it was found that increasing the P407 concentration led to a decrease. In contrast with other works, an increase of *T_gel_* was observed upon increasing the P188 or CS concentrations, as well as upon dilution with STF. CS did not seem to affect the gelation ability of the system; instead, it merely delayed the gel formation. It was demonstrated that CS contributed to the mechanical strength of the gels and that P188 contributed to resistance to dilution (increased gel stability). The % *w*/*w* concentrations of the hydrogel with the most favorable properties were: 14.2 for P407, 1.7 for P188 and 0.25 for CS. Four ophthalmic active pharmaceutical ingredients were entrapped in this gel: timolol maleate, dexamethasone, dorzolamide hydrochloride, and tobramycin. Upon loading, it was found that *T_gel_* decreased (except for tobramycin gel) and gelation time increased; however, the gel was not much affected, indicating its suitability for ocular delivery. In vitro release with timolol maleate was finally assessed. Compared to timolol solution, the hydrogel successfully achieved a more prolonged drug release. Finally, it was evidenced that the viscosity rather than the *T_gel_* was the critical factor that affected drug release.

Deepthi and Jose also prepared P407/CS hydrogels by the cold method [237]. The concentrations of P407 were 20% or 25% *w/w* and CS concentration ranged from 0.1% to 0.3% *w/w*. The obtained hydrogels were loaded with 0.3% of neomycin sulfate and 0.1% betamethasone sodium phosphate, aiming at the treatment of conjunctivitis. All the formulations were found to be transparent and clear, and *T_gel_* were measured between 36 and 39 °C. In vitro drug release was studied over a 500-min period. All formulations showed a sustained release, the ones with the higher polymer concentration, and thus higher viscosity, exhibiting the slowest release. Ex vivo permeation studies were performed with goat cornea and evidenced a slower release compared to the in vitro release due to the difficulty of the two drugs to pass through cornea.

In the previous examples, the drugs were directly loaded in the hydrogels; however, as it was mentioned earlier, it is a common practice to load gels with drug-loaded nanocarriers, whether inorganic or polymeric particles or vesicular systems. For example, Almeida et al. developed formulations combining ibuprofen-loaded NLC and a dual stimuli-responsive hydrogel (pH and temperature) based on CS and pluronic F-127 (F127) [238]. The concentration of F127 was optimized based on *T_gel_* after dilution with STF, and a value of 20% *w/w* was selected. As observed for F127, increasing the concentration of CS led to a decrease of *T_gel_*. The presence of F127 induced a decrease in the NLC surface charge as it partially masked it. When mucin-containing STF was added to the CS/F127 formulation, an increase in viscosity was observed as a result of the CS–mucin interactions. The increase was more important for more concentrated formulations of CS. A similar trend was observed for the mucoadhesive strength. Time- and concentration-dependent cytotoxicity was detected for the different tested formulations, which was attributed to cetyltrimethylammonium bromide used for the preparation of the NLC. NLC-loaded gels presented a lower cytotoxicity than pure NLC, and there were no cytotoxic effects at all for concentrations up to 250 mg/mL. The in vitro release of ibuprofen from the NLC/CS/PF127 hydrogel was slower than from either NLC or NLC/CS.

Ahmed et al. prepared a series of in situ gelling formulations of CS (0.5%), with ALG (1%) or P407 (16%) loaded with ketoconazole-loaded PLGA NPs [239]. Pure CS exhibited the longest gelation time (90 min), while the gelation time of the two CS mixtures were comparable (approximately 45 min). Sustained release was achieved; however, less than 40% of the drug was released in total from the CS/P407 formulation. Paulsamy et al. loaded CS/poloxamer (F127) in situ gelling formulations with nepafenac-containing silica NPs [240]. *T_gel_* was measured around 31–32 °C, and the formulations were found to be biocompatible. Between 84% and 89% of nepafenac was released from the in situ gelling systems within 12 h (in vivo). The release from the F127/CS system was found to be higher than that from a binary poloxamer system or pure drug suspension. Ex vivo corneal permeation followed the same trend. The authors attributed this to the lower rigidity of the CS/poloxamer gel compared to a pure poloxamer gel, allowing a higher drug release and corneal permeation.

Khan and his coworkers incorporated tobramycin sulfate-loaded CS microparticles in a P407/CS thermosensitive in situ gel for the sustained release of tobramycin sulfate [162]. After optimization, a concentration of 17% *w/v* for P407 was selected. More concentrated solutions gelled below 27 °C, while less concentrated solutions gelled above 35 °C. Viscosity increased with increasing CS concentration (0.5–1.5% *w/v*), with a parallel decrease in *T_gel_* and gelation time. A higher viscosity favors the entanglement and packing of poloxamer chains. The gelation of poloxamer is a result of the dehydration and subsequent chain-entanglement that the PPO fraction of poloxamer undergoes above 30 °C. CS “binds” the unbound water produced by PPO dehydration, thus facilitating chain entanglement. As a result, *T_gel_* decreases when CS is added. Sustained release was observed from the formulation and a slower release than for net microparticles or pure poloxamer hydrogel. This was attributed to the higher viscosity and the limited gel erosion (better mechanical properties). CS also contributed to the increase of mucoadhesive strength and corneal permeability of tobramycin sulfate. Finally, the aqueous humors of treated animals were analyzed for the presence of tobramycin: a therapeutically effective concentration against several important microorganisms (*Pseudomonas aeruginosa*, *S. aureus,* and *E. coli*) was attained within 15 min, and an important tobramycin concentration was still observed at 24 h. These results were better than those obtained with commercially available eye drops, demonstrating the potential of the tobramycin sulfate-containing P407/CS hydrogel for the effective treatment of endophthalmitis and bacterial conjunctivitis.

**Figure 13:**
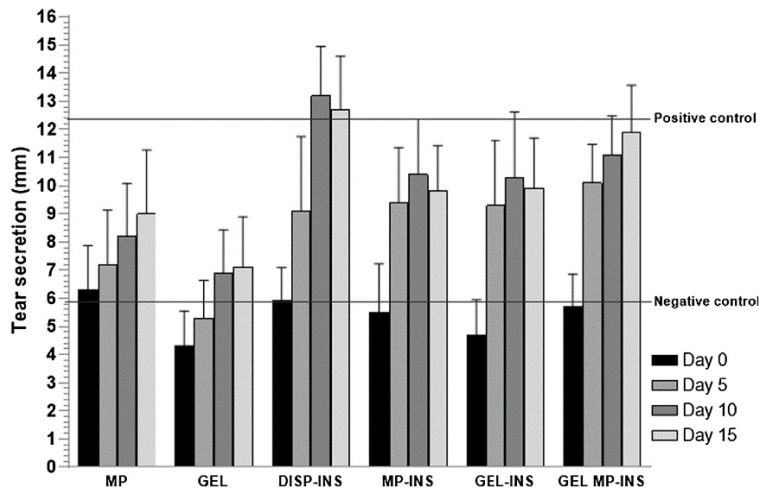
Tear secretion (mean ± SD, *n* = 5) from the eyes of treated animals as a function of time. Lower and upper horizontal black lines correspond to the mean tear secretion from untreated diabetic animals and healthy animals respectively in the 15 days of treatment. MP = microparticles, GEL = poloxamer 407/chitosan hydrogel, DISP-INS = insulin dispersion, MP-INS = insulin-loaded microparticles, GEL-INS = insulin-loaded hydrogel, GEL MP-INS = gel loaded with insulin-loaded microparticles. Reprinted from ref. [241]. Copyright 2019, with permission from Elsevier B.V.

Diabetes is well-known for being responsible for a series of eye-related conditions and diseases. Khan et al. and Cruz-Cazarim et al. reported a CS particle/CS-poloxamer hydrogel for the topical ocular administration of insulin for the treatment of dry eye syndrome and corneal injuries [241]. They prepared insulin-loaded CS microparticles that were incorporated in a CS/P407 thermoreversible hydrogel. CS was added to the poloxamer gel in order to improve its mechanical and rheological properties and increase the residence time of the gel on the ocular surface. *T_gel_* was around 32 °C. The ζ-potential of the hydrogel was positive due to the protonated amino groups of CS. The efficacy of the gels was evaluated in vivo by tear secretion. Animals treated with the microparticle-containing hydrogel showed a 77% increase in tear fluid volume, reaching levels comparable to the positive control (healthy animals) from the 5th day of treatment, which was earlier than all the other formulations, as illustrated in Figure 13. After 15 days of treatment, insulin was quantified in the lacrimal gland and eyeball of the treated animals and was found to be significantly higher in the group treated with the microparticle-containing hydrogel, indicating an increased local bioavailability. Finally, combined results from impression cytology and corneal epithelial thickness seem to indicate that hydrogel formulations allow a better restructuring of the corneal epithelium. In conclusion, the developed P407/CS hydrogel loaded with insulin-containing microparticles is an adequate platform for the topical delivery of insulin in the treatment of dry-eye symptom and corneal lesion.

PVA is another non-ionic polymer that is regularly used in ocular formulations and has been combined with CS. For example, Arvind and his coworkers have reported a clear PVA/CS formulation that underwent pH-triggered in situ gelation when pH was increased from 6 to 7.5 [242]. As PVA concentration increased, the viscosity increased. The highest swelling degree was exhibited by the formulation having intermediate concentrations of both polymers (3% *w/v* CS and 2% *w/v* PVA). This formulation also exhibited the highest cumulative release (94% in 6 h). This observation was in accordance with a swelling-controlled release. Gade et al. developed a three-component formulation based on CS, PVA, and PVP for the ocular delivery of fluoroquinolones, namely besifloxacin and levofloxacin [243]. The composition of the optimized hydrogels was 3% PVA/1% PVP/1% CS for besifloxacin and 1% PVA/0.5% PVP/0.5% CS for levofloxacin. Both drugs were released from their respective hydrogels within a 24 h time frame. The mucoadhesion of the hydrogels was significantly higher than corresponding marketed eye drop formulations (3.5 and 8 times higher for besifloxacin and levofloxacin, respectively). In vitro permeation studies showed improved performances for the gel formulations compared to marketed eye drops, with a shorter lag time and a greater flux. Finally, the drug-loaded hydrogels showed good antibacterial activity.

#### 4.2.2. Proteins

Gelatin is another polymer that is often combined with CS for the preparation of gels or in situ gelling formulations (Table 5). Gelatin is a protein derived from the hydrolysis of collagen and has been used for many years as an agent/excipient in food and pharmaceutical industries [244]. Gelatin is often crosslinked, chemically or physically, to increase its mechanical strength and decrease its solubility.

C. J. Liu, Y-C Cheng, S-H Chiou, and their coworkers prepared a series of thermosensitive CS/gelatin (type A)/β-GP hydrogels for the ocular delivery of several APIs [245,246,247,248]. Gelatin was used in order to improve the mechanical strength and gelation properties of CS [248]. The concentrations of CS and gelatin were 2–2.5% *w/v* and 0.2–1% *w/v*, respectively, *T_gel_* was around 34 °C, and the gelation time ranged from 40 to 80 s depending on the concentration of the polymers. Hydrophobic interactions were the driving force for gel formation above the gelling point [246,247]. The high molecular weight of the polymers as well as the hydrophilic moieties on CS and gelatin backbones (through hydrogen bonds) contributed to a high bioadhesive strength [245]. When the hydrogel was loaded with ferulic acid, sustained release was observed, with a cumulative release of 28% within 24 h [245]. In the rabbit corneal alkali burn model, accelerated corneal wound healing was detected after 3 and 9 h compared to the control group, indicating a good potential for the treatment of corneal alkali burn. Latanoprost for the treatment of elevated IOP in glaucoma patients was also entrapped in this formulation [246]. In vitro release was studied at 37 °C, and a progressive release from the hydrogel was observed, with a cumulative release of 52% within 7 days. The IOP-lowering effect was monitored in vivo, and the results demonstrated that a weekly application of the latanoprost-loaded formulation allowed for therapeutic levels of latanoprost in the ocular region, keeping IOP at normal levels. In contrast, Xalatan eye drops required a daily application to achieve a similar result. Later, a latanoprost/curcumin dual delivery formulation for glaucoma treatment was also developed, with curcumin-loaded PLGA NPs [247]. An extended release over 7 days was achieved for both curcumin and latanoprost (cumulative release on the 7th day: 7.1% and 23.6%, respectively). The therapeutic efficacy of the formulation was tested on human trabecular meshwork (TM) cells under H_2_O_2_-induced oxidative stress. It was shown that the curcumin-containing formulation could decrease the oxidative stress-mediated damage in TM cells. Finally, levofloxacin was also loaded in the CS/gelatin/β-GP hydrogel [248]. In vitro drug release studies evidenced a prolonged release over a 7-day period, while in vitro antibacterial studies against *S. aureus* and *Staphylococcus epidermidis*, which can cause endophthalmitis and other ocular infections, showed a clear zone of growth inhibition for at least 7 days. Finally, in vitro wound healing showed that the levofloxacin-loaded hydrogel promoted faster wound healing compared to levofloxacin alone.

**Figure 14:**
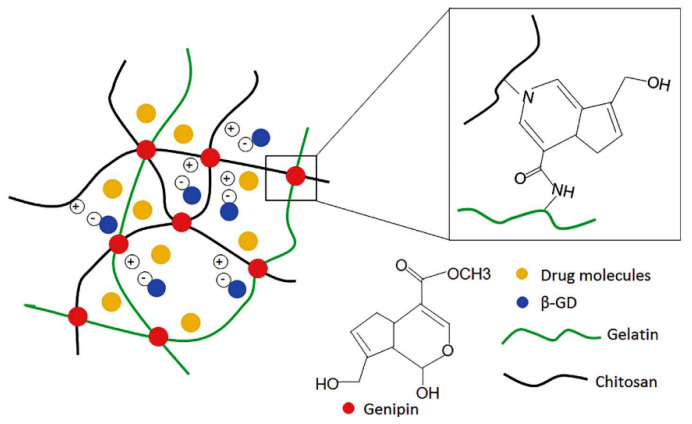
Schematic illustration of the β-glycerophosphate disodium salt (β-GP)/genipin co-crosslinked chitosan (CS)/gelatin hydrogel network. The mechanism of the sol–gel transition was attributed to the electrostatic attraction between positive-charged ammonium groups (⊕) of CS and negative-charged phosphate moieties of β-GP (㊀) and attractive hydrophobic and hydrogen bonding between chitosan chains. Reprinted from ref. [250]. Copyright 2018 with permission from Elsevier B.V.

Abe and his collaborators also used a gel composed of CS and gelatin as a drug matrix [249]. They developed a device for transscleral sustained drug delivery to the retina with a silicone refillable reservoir for the long-term treatment of posterior segment eye diseases. The reservoir was filled with an injectable gel bearing the drug. In this work, the CS/gelatin gel was crosslinked with EDC. EDC is a coupling agent that catalyzes the formation of amide bonds between amines (CS) and carboxylic acids (gelatin). Pure CS gels were brittle, and adding increasing amounts of gelatin resulted in an increase in the gel strength. Gelatin-only gels showed less strength than the stronger CS/gelatin gel. The best properties were exhibited by the gel composed of 3% gelatin and 1% CS. The gel revealed an initial burst release, and the overall release lasted less than 5 days. Predictably, when the gel was loaded in the device, the release was extended (2–5 times longer), and in vivo release was even longer (12 weeks). When the gel was injected in vivo in the device, the formulation crosslinked immediately, thus preventing any leakages. The CS/gelatin injectable gel could be loaded with different types of drugs, with varying size, lipophilicity, and physical state. The same group developed a double crosslinked CS/gelatin gel for timolol maleate ocular delivery [250]. The gel was co-crosslinked by β-GP and genipin. The CS/gelatin binary system could not gel; however, gelation occurred readily upon the addition of β-GP (0.8% *w/v*). Faster gelation was observed in the presence of an increasing concentration of gelatin. The addition of genipin, the second crosslinking agent, further decreased the gelation time. Gelation is a result of several interactions: electrostatic attractions between positively charged CS chains and negatively charged β-GP, hydrogen bonds between CS chains and between CS and gelatin, and the covalent bonds formed by the reaction of genipin with the primary amino groups of CS or gelatin (Figure 14). The CS/gelatin/β-GP/genipin system formed a porous, three-dimensional structure, as shown by SEM. Genipin increased the gel strength, but with increasing genipin content, the swelling ratio and the degradation rate decreased, and a denser hydrogel with smaller pores was formed. In accordance with these observations, increasing genipin content resulted in a slower release of sodium fluorescein (model compound). In vivo, the lowest IOP was measured 1 h after administration, and a lowering effect was detected up to 24 h after administration. In comparison, eye drops showed their maximum effect 0.5 h after administration and showed a therapeutic effect only for 12 h. Therefore, the developed formulation successfully enhanced the ocular bioavailability of timolol maleate by increasing its retention in the ocular region.

El-Feky et al. used oxidized sucrose as a crosslinking agent, which is expected to be less toxic than traditional crosslinking agents [251]. Sucrose is a disaccharide that is made of glucose and fructose. Upon mild oxidation, a tetraaldehyde bearing free hydroxyl groups is formed. Oxidized sucrose acts as a crosslinking agent through reaction with amines and alcohols, by forming imine bonds and acetals, respectively. A series of formulations with varying CS, gelatin, and oxidized sucrose concentrations were prepared. As evidenced by SEM images, the hydrogels were porous, with interconnected pores. Smaller pores and reduced swelling were observed when oxidized sucrose concentration increased as denser, more crosslinked hydrogels were formed. Mucoadhesion also decreased with increasing oxidized sucrose concentration. The in vitro release of timolol maleate showed a biphasic release, while in an in vivo pharmacodynamic study, a therapeutic effect was detected 30 min after administration, reaching its maximum after 2 h and lasting for 24 h. Commercial eye drops had a stronger effect for the first hour but only lasted 8 h.

Poly(gamma-glutamic acid) (PGGA) is an anionic homopolyamide composed of D and/or L-glutamic acid monomers. Chiesa et al. investigated the potential of PGGA in the formation of hydrogels for pharmaceutical applications [252]. The ternary combination of CS, PGGA, and β-GP showed thermogelling properties. Specifically, the gel prepared with a 1/2/5 ratio of CS/PGGA/β-GP showed a transition from a viscous solution to a gel when incubated for 20 min at 37 °C.

#### 4.2.3. Other Polysaccharides

CS has also been frequently combined with other polysaccharides, generally anionic, such as ALG or gellan gum (Table 6). Alginate forms readily stable hydrogels in the presence of divalent ions due to the interactions with carboxylic acid groups present on the polymeric chains and the formation of ionic bridges [253,254]. As tear secretion contains divalent ions, notably Ca^2+^ ions, ALG, as well as gellan gum and other similar anionic polysaccharides, are good candidates for the preparation of ionically triggered in situ hydrogels.

Gupta et al. reported a pH activated in situ gelling system consisting of ALG and CS for levofloxacin topical delivery [255]. A series of formulations were prepared, but based on viscosity, clarity, and flow properties, the formulation with 0.5% CS and 0.2% ALG was selected for further studies. The gelation pH for that formulation was around 7. The system gelled both upon the increase of pH, due to CS, and on interacting with divalent cations, due to ALG; thus, a good gel was expected to form in the ocular physiologic environment. The system demonstrated a prolonged release of levofloxacin, with an initial burst release and then a slower release (cumulative release at 8 h: 84%). Levofloxacin was radiolabeled with a technetium radionuclide, ^99m^Tc, and its distribution in the eye was imaged through the gamma emission of the radionuclide (gamma scintigraphy). The scintigraphic studies showed that commercial eye drops were rapidly cleared from the corneal region and were detected in kidney and bladder 2 h after administration. In contrast, the gel formulation was retained for a longer period in the corneal region, and no significant radioactivity was observed in systemic circulation. As mentioned earlier, Ahmed et al. prepared in situ gelling formulations of CS (0.5%) and with ALG (1%) or poloxamer 407 (16%), containing ketoconazole-loaded PLGA NPs [239]. Drug diffusion was higher from the CS/ALG formulation. That system also presented the highest percentage of drug release (around 80% in 8 h) and the highest antifungal activity. Finally, when transcorneal permeation was examined, CS/ALG formulation exhibited sustained drug permeation.

Gellan gum is a linear anionic polysaccharide produced by the bacterium *Sphingomonas paucimobilis* (ATCC31461) [256]. It is commercially available under the names Kelcogel and Gelrite and is used as solidifying agent, a thickener, and a gelling agent. Gupta et al. reported a CS/gellan gum formulation for the administration of sparfloxacin [257]. Different compositions were studied; the concentrations of the optimized formulations were 0.5% for CS and 0.2% for gellan gum. In vitro sparfloxacin release was examined in STF, and a 91% cumulative release was observed in 12 h (the corresponding eye drop formulation released sparfloxacin within 4 h). Gamma scintigraphy showed a good spreading over the entire precorneal area and satisfactory retention in the corneal surface. Ameeduzzafar et al. evaluated a series of CS/PVA/gellan gum for the preparation of a besifloxacin-containing in situ gelling system for the topical treatment of bacterial conjunctivitis [258]. Different concentrations of each polymer were used, and the resulting formulations were evaluated in regard to their gelling capacity. Gelation was induced by the addition of STF. Gelling strength increased with increasing concentrations of CS and gellan gum. Transcorneal permeation of the besifloxacin-loaded optimized formulation was nearly 95%, which represented a 2.5-fold increase compared to besifloxacin suspension. The optimized formulation showed a prolonged antimicrobial efficacy, and when tested in vivo in rabbit eyes, the maximum concentration of besifloxacin aqueous humors observed from the CS/PVA/gellan gum formulation was 1.6 times higher than the drug suspension. Gamma scintigraphy evidenced the absence of radioactivity in systemic circulation, suggesting a good retention of besifloxacin in the ocular area.

**Figure 15:**
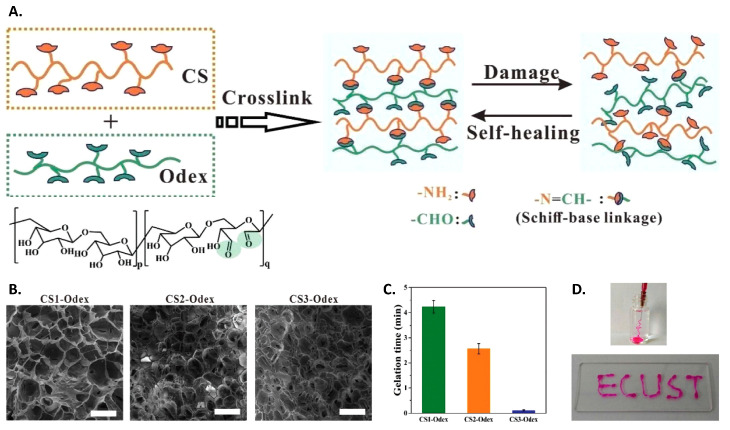
(**A**) Schematic of the formation of the self-healing injectable chitosan/oxidized dextran (CS/Odex) hydrogel via a dynamic Schiff base linkage. (**B**) Scanning electron microscopy images of the microstructures of the CS/Odex hydrogel (scale bar: 200 μm). (**C**) Gelation time of the CS/Odex hydrogel for different CS concentrations. Error bars represent mean ± SD; *n* = 3. (**D**) Injectability of rhodamine B-stained CS/Odex hydrogel through a 26 G needle and hydrogel stability after its injection into water. Reproduced from ref. [259] with permission from The Royal Society of Chemistry.

Dextran is a branched polysaccharide of glucose. Jiang et al. fabricated a CS-based, self-healing hydrogel for the delivery of retinal progenitor cells to the retina for the treatment of retinal degeneration via stem-cell therapy, with oxidized dextran (Odex) [259]. The hydrogel was prepared with CS and a polyaldehyde synthesized by dextran oxidation, via the formation of Schiff base bonds between the aldehyde functional group of Odex and the amine groups of CS (Figure 15). These bonds are dynamic reversible bonds that can be formed and cleaved and re-formed, and, as a result, the hydrogel presented self-healing properties. Gelation was easily manipulated by changing the CS concentration (1–3% *w/v*) of the formulation. The hydrogel with the lowest CS concentration had the slowest gelation time and the lowest crosslinking density, resulting in the highest swelling (highest water absorption) and the fastest degradation. It was further demonstrated that the proliferation of retinal progenitor cells was improved by the CS/Odex gel, and this was correlated to the stress relaxation properties of the gel. Finally, the differentiation of retinal progenitor cells toward neuron was favored.

Muţ et al. investigated a series of CS/HPMC based hydrogels for fluconazole (antifungal agent) ocular delivery [260]. Sucrose laurate or sucrose palmitate were added as penetration enhancers. Upon their addition, most formulations exhibited a lower viscosity than the CS/HPMC base hydrogel. The highest cumulative release was obtained with the formulation containing 0.5% sucrose laurate. Ex vivo permeation experiments showed that fluconazole permeated through pig ear skin faster and in higher quantities from the hydrogel with the highest amount of sucrose laurate (5%).

### 4.3. Hydrogels from Modified Chitosan

**Scheme 3:**
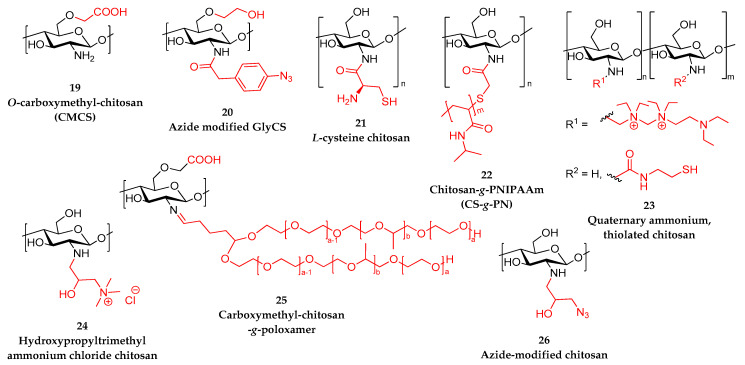
Modified repeating units of chitosan derivatives used for the preparation of hydrogels.

**Figure 16:**
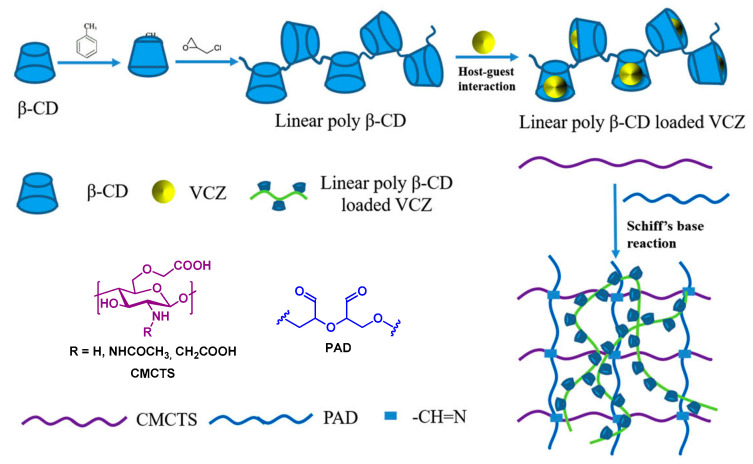
Preparation of the voriconazole (VCZ)-loaded injectable hydrogel (β-CD: β-cyclodextrin, CMCTS: carboxymethyl chitosan, PAD: polyaldehyde from oxidized dextran). Reprinted from ref. [261]. Copyright 2019, Wiley Periodicals, Inc.

Yang et al. prepared a CS-based hydrogel via a Schiff base reaction by reacting carboxylmethyl CS (CMCS), **19** in Scheme 3, with Odex (Table 7) [261]. The gel formed spontaneously upon mixing the solutions of the two components. Voriconazole (VCZ), an anti-fungal agent used in the treatment of fungal endophthalmitis, was incorporated as an inclusion complex with poly(β-cyclodextrin). The linear chain of poly(β-cyclodextrin) intertwined in the CMCS/Odex network, as shown in Figure 16. Drug release was a combination of swelling, diffusion, and chemical degradation. Likewise, Xu et al. also used CMCS for the preparation of an in situ self-crosslinking hydrogel with the polyaldehyde obtained by the oxidation of ALG this time [262]. Upon mixing aqueous solutions of the two components, the gel formed at room temperature within 60 s. Gelation was attributed to the Schiff base reaction between the CHO groups of oxidized ALG and the NH_2_ groups of CMCS. Limbal stem cells were transplanted in the hydrogel, and it was shown that when applied to corneal alkaline burn, cornea opacity was completely reduced within 28 days; in other words, remarkable corneal healing reconstruction was achieved.

Similarly to Yang et al., Lei et al. exploited the formation of a Schiff base between the amino groups of glycol CS (GlyCS), **9** in Scheme 1, and 4-arm PEG with aldehyde end-groups, to prepare porous hydrogels [263]. The gelation and mechanical properties of the gel could be tuned by changing the concentrations of the two components. Levofloxacin was entrapped in the gel, and sustained release over 24 h was observed. The gel had a good cytocompatibility (in vitro) and caused no irritation in in vivo tests. Qiao et al. further modified GlyCS on the free –NH_2_ moieties by conjugation with 4-azidophenylacetic acid, in order to introduce azide groups, –N_3_, **20** in Scheme 3 [264]. The resulting modified CS exhibited a photosensitive behavior and could gel within 30 s upon UV irradiation. The hydrogel demonstrated good in vivo biodegradability and biocompatibility. Once loaded with heparin, the hydrogel was implanted intraocularly and showed potential in limiting post-operative complications after glaucoma surgery.

Due to the easy crosslinking via the formation of disulfide bonds, thiol modifications of CS have attracted interest in the field of hydrogels. For example, Moreno and others prepared thiolated CS-based hydrogels for the administration of two anti-vascular endothelial growth factor (anti-VEGF) protein drugs, ranibizumab and aflibercept, to treat choroidal or corneal neovascularization [265]. In this study, CS was coupled to L-cysteine, **21** in Scheme 3, or 6-mercaptonicotinic acid, via its amino groups. Hydrogels were formed through oxidation in air, within several hours. Increasing the substitution degree afforded gels with higher crosslinking density and lower swelling, as well as a lower burst release in in vitro drug release studies, but also a higher retention of the two proteins within their network. Release was mainly controlled by the hydrogel swelling. Finally, it is important to note that despite the –SH groups of the modified CS, the structures of the protein drugs remained intact, and the proteins retained their activity after release.

Thermogelling polymers have received extensive interest because of their ability to gel at body temperature while being liquids at room temperature. Poly(*N*-isopropylacrylamide) (PNIPAAm) is a well-known temperature-responsive polymer that has a lower critical transition temperature (LCST) relatively close to physiological temperature and has been widely used for biomedical applications. The research groups of J.-Y. Lai and L.-J. Luo grafted carboxylic acid end-capped PNIPAAm chains onto CS using a carbodiimide coupling reaction [266,267,268]. The resulting modified CS (CS-*g*-PN), **22** in Scheme 3, was used for the preparation of novel in situ gelling systems for ocular delivery by intracameral administration. The LCST of CS-*g*-PN depended on the amount of PNIPAAm grafted onto CS and was around 30–32 °C, decreasing with increasing PNIPAAm concentration (Table 8) [266]. The gels were loaded with pilocarpine nitrate, which is a drug used in glaucoma treatment. In vitro drug release studies showed a continuous release over 6 weeks, and in vivo studies showed a similar IOP-lowering effect, evidencing that a therapeutic concentration of pilocarpine was maintained throughout this period. Drug release was attributed to the biodegradation of the hydrogel: biodegradation which was found to be dependent of the PNIPAAm content. The impact of the deacetylation degree of CS on the properties of CS-*g*-PN was further studied [267]. It was initially determined that the deacetylation degree did not affect PNIPAAm grafting. Significant differences were observed in LCST with values ranging from 26.5 °C for the lowest deacetylation degree to 31.8 °C for the highest. Increasing the deacetylation degree resulted in slower degradation, a lower drug entrapment, and a slower release. The intermediate deacetylation degree was found to be optimal due to a better biocompatibility, and that formulation achieved an IOP-lowering effect over 63 days. In line with that work, CS-*g*-PN was functionalized with a series of methoxy-substituted benzoic acids to confer antioxidant properties. In parallel, the simultaneous delivery of pilocarpine and RGFP966 was investigated [268]. The results demonstrated that the increase of the methoxylation extent was accompanied by a strengthening in the antioxidant properties. In other words, among the synthesized derivatives, the one prepared with 3,4,5-trihydrobenzoic acid exhibited a pro-oxidant behavior, while the ones prepared with 3,4-dihydroxy-5-methoxybenzoic acid and 4-hydroxy-3,5-dimethoxybenzoic showed the strongest antioxidant behavior. The latter showed a drug release extending over 70 days for both APIs and showed the highest efficiency in in vivo studies, demonstrating a good potential in the treatment of glaucomatous optic neuropathy.

As discussed earlier, quaternary ammonium chitosans are one of the most important hydrophilic CS derivatives. Fabiano et al. prepared CS hydrogels replacing small amounts of CS by different quaternized, **12** in Scheme 1, or quaternized and thiolated, **23** in Scheme 3, CS derivatives to improve mucoadhesiveness [178,203]. CS was crosslinked with β-GP and exhibited a sol–gel transition around 30–35 °C. The gels were further loaded with CS/HA NPs containing 5-fluorouracil. 5-fluorouracil release was diffusion controlled and prolonged compared to the release from the NPs alone. When comparing the gels obtained by derivatives **12** and **23**, the former exhibited better characteristics during drug release studies [178]. In in vivo tests, these gels demonstrated a constant 5-fluorouracil concentration in aqueous humors from 0.5 to 7 h after instillation and a significant improvement in bioavailability.

Tan et al. used another quaternized CS derivative for the preparation of a thermosensitive in situ gelling formulation [269]. Indeed, hydroxypropyltrimethyl ammonium chloride CS, **24** in Scheme 3, forms a thermosensitive gel when mixed with β-GP. By optimizing their concentrations, a formulation that gelled within 2 min at 35 °C was obtained. Dexamethasone-loaded NLCs were further incorporated to the formulation. A sustained release over 72 h with a reduced initial burst release was observed.

**Figure 17:**
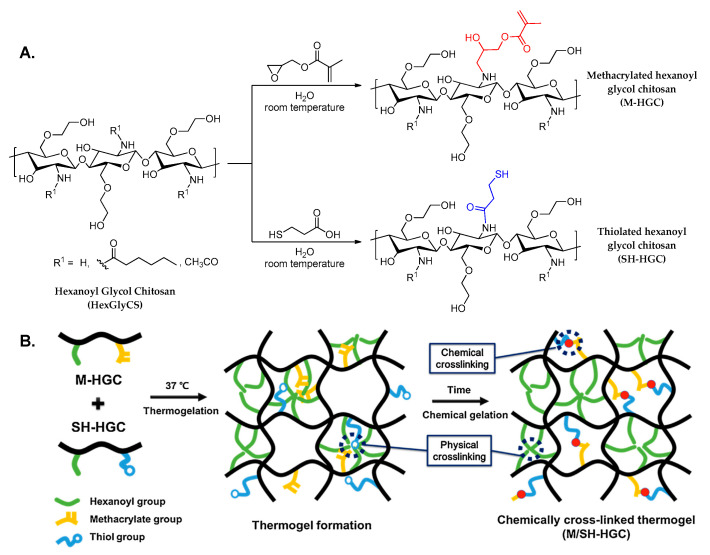
(**A**) Synthesis of methacrylated (M-HGC) and thiolated (S-HGC) hexanoyl glycol chitosan (HexGlyCS). (**B**) Formation of the chemically crosslinked hydrogel. Adapted from ref. [272]. Copyright 2019 with permission from Elsevier B.V.

Poloxamers and PNIPAAm, which are well-known thermoresponsive polymers, are also non-degradable and may contain traces of toxic monomers. As an alternative, Cho et al. [270] and Shi et al. [271] synthesized hexanoyl GlyCS (HexGlyCS), as shown in Figure 17, by the *N*-acylation of GlyCS and investigated its in situ forming gels. Gelling is the result of the formation of a thermally induced physical network due to hydrophobic interactions between hexanoyl groups. *T_gel_* ranged from 25 to 50 °C depending on the concentration of HexGlyCS and the degree of hexanoylation [270]. A decrease in *T_gel_* was observed when increasing HexGlyCS concentration or the hexanoylation degree. When loaded with brimonidine tartrate, the in vitro drug release from the gels was similar to Alphagan P, which is a marketed brimonidine formulation. However, in vivo, the IOP-lowering effect was significantly prolonged due to the improved pre-ocular retention of the gel. When levofloxacin was added to it, the hydrogel exhibited a sustained in vitro release over 12 h, while the in vivo pharmacokinetic study demonstrated an enhanced ocular bioavailability [271].

Starting from HexGlyCS, Oh et al. prepared two complementary CS derivatives, as described in Figure 17A [272]. The first one, M-HGC, was synthesized by *N*-methacrylation, in order to bear a double bond. The second one, S-HGC, was modified in order to introduce a thiol group [79]. Both polymers on their own, as well as in combination, exhibited thermogelling behaviors with sol–gel transition temperatures ranging from 25 to 35 °C. When solutions of the two derivatives were mixed and incubated at 37 °C, a gel was formed within *circa* 6 h due to the Michael addition of the thiol to the double bond. As illustrated in Figure 17B, physical crosslinking was thus reinforced by additional chemical crosslinking, but without using potentially toxic crosslinking agents, such as glutaraldehyde. The gels were non-cytotoxic; their degradation and swelling depended on the crosslinking density and could be tuned by the functionalization degree.

Kong et al. reported a CS/CMCS, **19** in Scheme 3, thermosensitive hydrogel, which was crosslinked in the presence of β-GP, for the delivery of levofloxacin encapsulated in CS microspheres [153]. A slow release of levofloxacin, with no burst release, and a prolonged ocular retention were observed. Yu et al. prepared a P407/CMCS thermosensitive hydrogel for the ocular delivery of nepafenac [273,274]. To increase the mechanical strength of that gel, chemical crosslinking was performed with glutaraldehyde, **25** in Scheme 3. The resulting gel exhibited temperature- and pH-dependent swelling and the *T_gel_* temperature was around 32–33 °C. Swelling could be tuned by modifying the concentration of P407, CMCS, and glutaraldehyde. The highest swelling (18,200%) was measured at pH 7.4 and 35 °C. Accordingly, when loaded with nepafenac, nepafenac release was the highest and fastest under those conditions: an almost complete release within 72 h (slower than nepafenac), while at lower temperature and/or pH, release was not complete [273]. To further delay release and improve the performance of this delivery system, nepafenac-loaded NLCs were incorporated to the hydrogel instead of neat nepafenac [274]. Indeed, a slower release was achieved, with a lower burst release compared to the nepafenac-loaded hydrogel. When in vivo behavior was studied, the concentration of nepafenac in aqueous humor was significantly higher (14.4 times) from the gel compared to nepafenac eye drops, and the maximum concentration was reached later. This was attributed to the improved corneal permeation due to the NLC and the longer retention on cornea due to the hydrogel formulation.

Yu et al. further improved this delivery system by replacing glutaraldehyde by genipin, which has a much lower toxicity and exhibits some anti-inflammatory properties [275,276]. In contrast to glutaraldehyde, genipin could only react with the amino groups of CMCS [276]. However, the semi-interpenetrating network that was formed retained a pH- and temperature-dependent behavior. The swelling ratio was higher at 35 °C and pH 6.5 [276] or 7.4 [275]. Increasing the genipin concentration limited swelling [275], while increasing the CMCS ratio resulted in a higher swelling ratio [276]. Quercetin-loaded NLCs were incorporated to the gel, and an improved drug release was observed [275]. Similarly, baicalin-loaded NLCs were integrated to the CMCS/P407/genipin hydrogel, affording enhanced in vitro release, corneal permeation, and pre-corneal retention [276].

Lin et al. exploited the thermosensitive properties and in situ gelling behavior of hydroxybutyl CS for the design of intracanalicular plugs for the treatment of dry eye disease [277]. Hydroxybutyl CS gelled within 50 s at 37 °C and thus intracanalicular injection could be applied to set the plug in place, providing ease of operation compared to other materials.

## 5. Ocular Lenses and Inserts

Ocular delivery for the treatment of various diseases traditionally occurs via aqueous solutions or suspensions, which are often characterized by low therapeutic efficacy due to the clearing mechanisms of the eye. Contact lenses and inserts (non-implantable drug delivery devices) have been investigated as an alternative way of topically administrating drugs to the ocular region, as they improve patient compliance and promote the targeted delivery of various drugs to the lens. CS as a natural polysaccharide has been studied from many research groups, as it provides the final lenses or implants with mucoadhesiveness, biocompatibility, and good mechanical properties.

Solvent casting is a convenient method to prepare films and membranes that can be used as ocular inserts. For example, Franca et al., while designing an HPLC validated method for bimatoprost, prepared CS ocular inserts for the controlled release of bimatoprost via solvent casting [278]. A homogenous distribution of the drug in the inserts was observed, while its release was controlled. Foureaux et al. prepared diminazene aceturate-loaded ocular inserts for glaucoma treatment [279]. The inserts, also prepared by solvent casting, were obtained as flexible membranes. After one week of treatment with these inserts, the IOP was reduced to normal levels, and these levels were further maintained for four weeks. Compared to eye drops, biodistribution studies with radiolabeled drugs revealed significantly more diminazene aceturate in the ocular region and significantly less in the gastrointestinal tract. The same team, again for glaucoma treatment, prepared membranes of CS and hydroxyethyl cellulose loaded with dorzolamide [280]. Dorzolamide was molecularly dispersed within the polymeric matrix, and the formation of hydrogen bonds was evidenced. It was further observed that the addition of dorzolamide reduced the swelling of the film, which was possibly due to the formation of the aforementioned hydrogen bonds. It is noteworthy that after 18 h, more than 50% of dorzolamide is retained in the eye, as detected by scintigraphy. In vivo, an IOP decrease for two weeks was measured for those ocular inserts. Similarly, Wang et al. reported the preparation of inserts for glaucoma treatment [281]. The inserts in this case were nanosheets that were prepared by CS and ALG for the dual delivery of timolol and latanoprost. The nanosheets were highly transparent, flexible, and adhered to the ocular surface. In vivo studies exhibited a IOP decrease for at least 9 days, suggesting an efficient drug delivery.

Abilova et al. prepared films from aqueous solutions of CS and poly(2-ethyl-2-oxazoline) and examined their potential in ophthalmic applications [282]. Transparent, homogenous films were obtained via solvent casting. Intermolecular hydrogen bonds formed spontaneously between the functional groups of the two polymers. Results concerning the mechanical properties of the blends revealed that increasing the percentage of poly(2-ethyl-2-oxazoline) resulted in diminishing the mechanical properties of the blends as it made the films more brittle. Ex vivo mucoadhesion studies on freshly excised bovine cornea revealed that upon increasing the poly(2-ethyl-2-oxazoline) percentage, the retention capability of the films in the eye was increased. In vivo studies on rabbit ocular corneas revealed excellent adhesion to the cornea and no irritancy.

Üstündag-Okur et al. reported novel ofloxacin-loaded NLC inserts for the treatment of bacterial keratitis [283]. The inserts were prepared from chitosan oligosaccharide lactate via solvent casting evaporation, while glycerin was added as a plasticizer. The presence of CS provided the inserts with high mucoadhesiveness. The in vitro release of ofloxacin in PBS (pH = 7.4) during the first 24 h was satisfactory. In vivo tests on white rabbits confirmed that the pre-ocular retention was extended to 24 h, making these NLC inserts suitable for occular delivery. Jeencham et al. utilized the film-casting technique to prepare blends between CS and regenerated silk fibroin in various ratios [284]. During the preparation of contact lenses, PEG 400 was employed as a plasticizer. The films prepared with a high percentage of CS revealed good mechanical properties, high thermal stability, and light transparency over 90%. FTIR spectra confirmed the presence of intermolecular hydrogen bonds, indicating the good compatibility of the polymers. The films presented excellent physicochemical properties (high water content, high ion and oxygen permeability) as well as non-cytotoxicity, indicating their promising potential as a biomaterial for a contact lenses-based ophthalmic drug delivery system.

**Figure 18:**
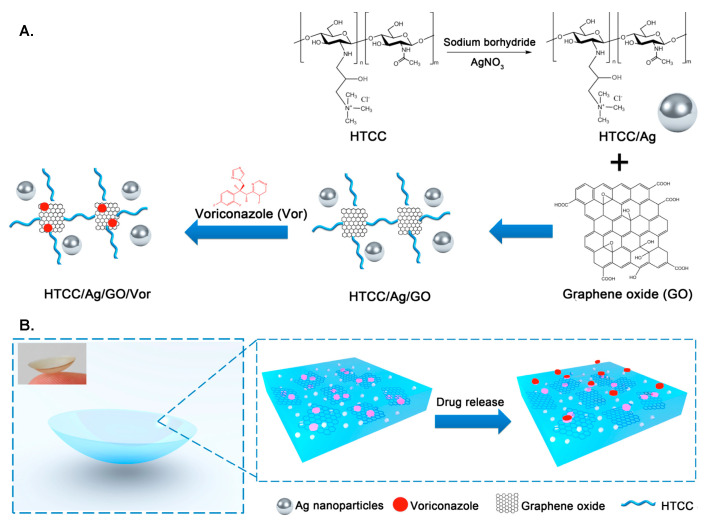
(**A**) Preparation of *N*-[(2-hydroxy-3-trimethylammonium)propyl]chitosan chloride (HTCC)/silver (Ag)/graphene oxide (GO)/voriconazole (Vor) delivery system. (**B**) Schematic illustration of voriconazole-loaded contact lens and controlled drug release. Reprinted with permission from ref. [285]. Copyright, 2016, American Chemical Society.

In Huang’s et al. research, hydrogel contact lenses with unified anti-fungal properties were prepared and assembled as a drug delivery system for the management of fungal keratitis [285]. More specifically, *N*-[(2-hydroxy-3-trimethylammonium)propyl]chitosan chloride, **24** in Scheme 3, was synthesized via a ring-opening reaction, followed by the addition of silver NPs (formed by in situ reduction of silver salts), low carboxylation graphene oxide, and voriconazole for the final production of the nanocomposites, as shown in Figure 18. The resulting formulations were examined for their thermal stability, their in vitro voriconazole release, their antimicrobial properties, and their cytotoxicity activity. The contact lenses enhanced the release of voriconazole in the cornea, and the formulated nanomatrices were non-toxic with improved biological properties. This innovative drug delivery system could be a promising solution for the treatment of fungal keratitis.

**Figure 19:**
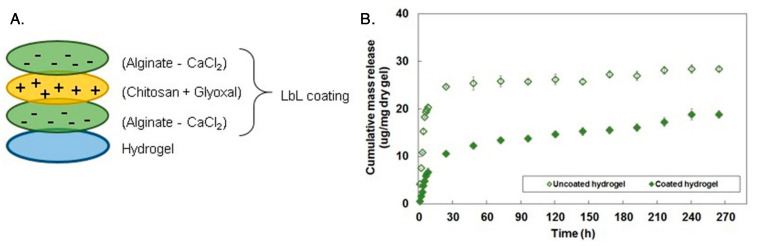
(**A**) Layer-by-layer (LbL) coating of contact lenses. (**B**) Cumulative drug release of diclofenac. Adapted from ref. [286]. Copyright 2016, with permission from Elsevier B.V.

Silva et al. capitalized upon the layer-by-layer deposition method to combine CS and alginate for the coating of three kinds of lenses, as shown in Figure 19 [286]. Coated lenses were investigated for the controlled release of the anti-inflammatory agent, diclofenac. The multiple coating was composed of (alginate–CaCl_2_)/(CS + glyoxal), which was covered with an ultimate alginate–CaCl_2_ layer, intending to prevent CS disintegration by tear liquid proteins, and it is quite noteworthy. The coating managed to reduce the initial burst release and afforded a 7-day sustained release. Additional investigation in CS functionalization for a better control over the drug release is required. Hu et al. also applied the layer-by-layer deposition to prepare HA/CS multilayers on PHEMA/*β*-cyclodextrin lenses aiming at the delivery of orfloxacin and puerarin [287]. The lenses were modified by azide groups, and the layers (up to 10) were further installed by click chemistry alternating between propargylic-modified HA and azide-modified CS, **26** in Scheme 3. The addition of HA/CS layers contributed to increasing the hydrophilicity of the lens. A coarser surface with a lamellar structure was also observed.

Kazemi Ashtiani et al. reported the surficial conjugation of PHEMA lenses with the cationic, and thus friendly to anionic pharmaceutical agents, CS for the delivery of ascorbic acid [288]. Protein adsorption, biocompatibility, and antibacterial characteristics of the fabricated hydrogels were surveyed after CS stabilization. As stated, CS-conjugated contact lenses were transparent and biocompatible, whereas this material was suggested as a viable substitute to commercial drug formulations.

The development of novel coatings using CS and borneol for ocular contact lenses was proposed by Mehta et al. [289]. In that study, the formulations loaded with timolol maleate were prepared via electrohydrodynamic atomization using PVP and PNIPAAm as stabilizers. Moreover, the group examined the particle size, the thermal stability, their in vitro release profile, and their ocular tolerability. The results indicated a wide range of sizes in the formulated samples, depending on the concentration of CS. Furthermore, differential scanning calorimetry revealed the amorphous state of the drug in the synthesized coatings. In vitro release study and kinetic modeling certified the importance of CS, as the swelling capacity of the polymer improved successfully the release of the active substance.

Hoyo et al. developed effective biocompatible and antibacterial coatings on contact lenses via a single-step sonochemical process, in order to enhance their convenience and improve their biological properties [290]. The formulations consisted of ZnO NPs, CS, and gallic acid (GA). The resulting hybrid CS/GA/ZnO coating increased the surface antioxidant activity by 95%. Moreover, the triple-loaded hybrid structures exhibited significant antibacterial efficacy and improved biocompatibility to human cells. These interesting characteristics were expected to improve the comfort of contact and intraocular lenses.

Saher et al. prepared porous levofloxacin-loaded semi-sponges for ocular bacterial infections [291]. The semi-sponges could be inserted in a lower cul-de-sac and could eliminate the frequent use of ophthalmic drops. Various polymeric matrixes (LMWCS, HMWCS, sodium carboxymethyl cellulose, and Gelrite) were investigated, and the sponges were prepared by freeze drying. In vitro release studies in STF (pH = 7.4, 35 °C) correlated the release behavior of the samples to their pore size. The LMWCS samples had a small pore size, which resulted in a prolonged release of levofloxacin up to 12 h, while HMWCS had a larger pore size, causing a faster release. Furthermore, the semi-sponges were tested in vivo in rabbits and were found to be non-irritant. Manna et al. prepared methotrexate-loaded CS micro-implants by freeze drying. It was observed that a lipophilic poly(lactide) (PLA) or PLGA coating contributed to a better control over burst release and could extend methotrexate release up to 5 months, depending on the coating [292].

Mirzaeei et al. examined CS-based electrospun nanofibers as ocular inserts for the delivery of triamcinolone acetonide [293]. Hydrophilic fibers were prepared using CS and PVA and/or poly(vinyl pyrrolidine) and compared to hydrophobic fibers constituted of Eudragit S100 and Zein. By SEM microphotographs, it was concluded that fibers from CS and poly(vinyl pyrrolidine) had smaller diameters (120 ± 30 nm) and were more uniform. Triamcinolone release from those fibers exhibited no burst release and a total release within 4 days, which was fitted to a zero-order kinetic profile. Although an even slower release was observed from the hydrophobic fibers, it was expected that the CS fibers would be more appropriate for ocular delivery.

## 6. Chitosan Coatings

CS is a biocompatible polymer that, among others, can be used to enhance the mucoadhesion of drug formulations to tissues. Furthermore, the thiolation of CS can improve the penetration of NPs or their content through the tight junctions of mucus layers. Numerous studies have been conducted on the use of CS or its derivatives as coatings in order to deliver drugs encapsulated in liposomes, solid lipid matrixes, or NPs to the eye.

**Figure 20:**
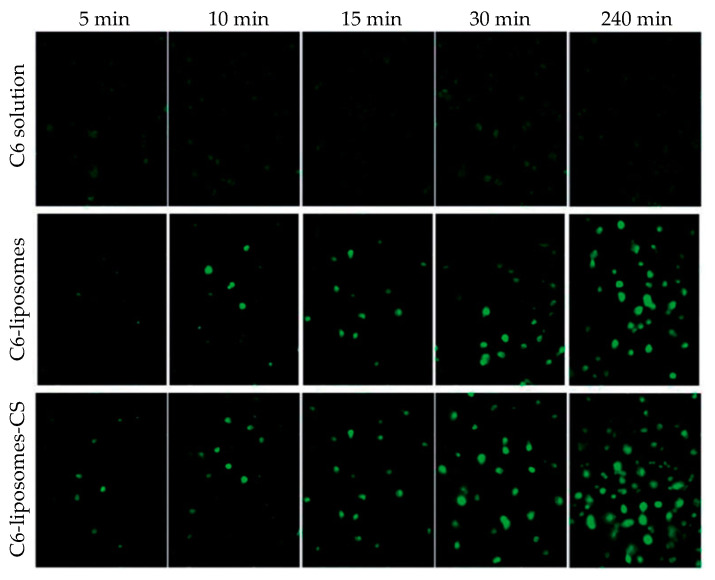
Cellular uptake of triamcinolone acetonide/coumarin 6 liposomes (C6-liposomes) and chitosan-coated triamcinolone acetonide/coumarin 6 liposomes (C6-liposomes-CS) by corneal epithelial cells at different times. Reprinted from ref. [294]. Rights managed by Taylor and Francis.

Li et al. encapsulated triamcinolone acetonide, which is an intermediate-acting glucocorticoid applied in inflammatory, edematous, and angiogenic ocular diseases, in liposomes, and the resulting NPs were further coated with CS [294]. The coating of NPs resulted in a slight increase of the size, i.e., from 108 to 135 nm, while the ζ-potential was inverted from −10 to +18 mV. Coated formulations were stable for a 60-day period at 4 °C, although a slight decrease (about 5%) in the drug entrapment efficiency was observed. Concerning the release profile of the drug, CS coating enhanced drug release compared to the uncoated NPs. Cellular uptake was enhanced by the CS coating, as evidenced by fluorescence microscopy (Figure 20). In vivo studies, performed on C57BL/6 mice, showed that the CS coating of liposomes led to a more efficient ocular delivery of triamcinolone acetonide to the posterior segment of the eye than eye drop formulation. Ocular toxicity tests showed that no toxicity was observed for either coated or uncoated liposomic NPs. The efficiency of these CS-coated liposomes in the treatment of retinal edema was further studied [295]. Experiments that were conducted in vivo using rat models concluded that CS-coated liposomes could deliver the drug into the retina of the eye. Furthermore, the formulation was found to effectively relieve retinal edema, caused by laser, without showing toxicity.

Tan et al. developed CS-coated liposomes for the ocular delivery of timolol maleate [296]. The drug-loaded liposomes were prepared using an ammonium sulfate gradient coupled with a pH gradient. The CS coating was applied simply by stirring the liposomes in a CS solution. Increasing the CS concentration afforded bigger particles with an initially increasing ζ-potential. Compared to timolol maleate eye drop formulation, in vitro release from the coated liposomes was extended over 12 h with a lower initial burst release. Transcorneal permeation was significantly enhanced, and ocular retention was improved as well. As a result, the IOP lowering efficiency was improved. Khalil et al. also prepared CS-coated liposomes for the nanoencapsulation of triamcinolone acetonide for posterior eye delivery [297]. The research team succeeded in preparing uncoated NPs of 18 nm in size while after coating with CS in concentrations of 0.1%, 0.2% and 0.3% *w/v*, their size increased to 100, 170, and 176 nm. In vivo studies were conducted in a rat model showing that after 15 days, the drug was present in the posterior chamber of the eye, which is a conclusion that is in accordance with the studies conducted by Li et al. [294] and Cheng et al. [295].

Chen et al. prepared CS-coated deformable liposomes for the ocular delivery of flurbiprofen [298]. The prepared NPs of deformable liposomes containing the drug were further coated by CS (M_w_ 50 kDa, degree of deacetylation: 95%) in three different concentrations (0.1%, 0.2%, and 0.4% *w/v*). It was found that the coated NPs were larger than the uncoated ones, and the ζ-potential changed from negative to positive. A corneal penetration study showed that deformable liposomes had a 1.5-fold increased penetration compared to non-deformable ones, while their penetration ability was further enhanced by the CS coating. Indeed, CS can improve the permeability of the cornea by opening the tight junctions among corneal epithelial cells or by intracellular routes (*vide supra*). A notable observation was that the penetration rate was different for the different formulations: all uncoated formulations showed a constant penetration rate, while the coated ones showed a reduced rate after 160 min. The cumulative penetration remained higher for the coated particles. In vivo pre-corneal retention showed that the CS coating significantly prolonged the residence time of deformable liposomes in the cornea and improved the drug bioavailability by increasing its transport time across the cornea.

**Figure 21:**
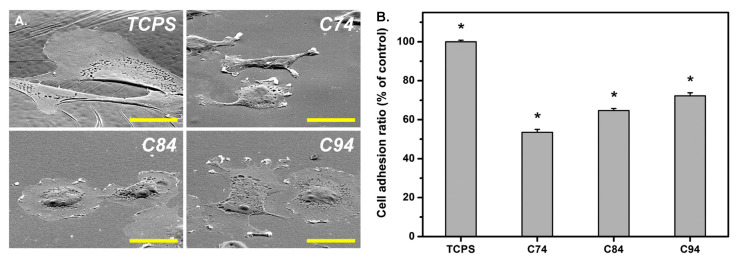
(**A**) Rabbit corneal keratocyte morphology on tissue culture polystyrene (TCPS) and chitosan substrates with various deacetylation degrees: 74% (C74), 84% (C84), and 94% (C94). Scale bar represents 30 μm. (**B**) Quantitative measurement of cell adhesion ratio for TCPS and various chitosan samples (* P b 0.05; *n* = 4). Data in the experimental groups are percentages relative to those of the TCPS groups. Reprinted from ref. [299]. Copyright 2016, with permission from Elsevier B.V.

Sun et al. studied for the first time the influence of the deacetylation degree of CS on corneal keratocyte adhesion, spreading, morphology, and integrin gene expression when used as a coating in pharmaceutical formulations [299]. The authors used CS with a molecular weight of 400 kDa, having three different degrees of deacetylation (74.1 ± 0.5%, 84.4 ± 0.7%, and 94.2 ± 0.5%). Initially, CS was 74% deacetylated. Further deacetylation was conducted via CS treatment with 60% (*w/v*) NaOH solution for 1 h once and for 1.5 h twice at 100 °C under a nitrogen atmosphere to obtain deacetylation degrees of 84% and 94%, respectively. FTIR and gel permeation chromatography showed that deacetylation did not affect the initial molecular weight. Crystallinity increased as the degree of deacetylation increased, which was due to the absence of acetyl groups. An important issue revealed by the study is that the degree of deacetylation affected cell adhesion, i.e., an increased deacetylation degree resulted in enhanced cell adhesion (Figure 21), which was probably due to the high stiffness and crystallinity of the biopolymers. It was suggested that the degree of deacetylation of CS coatings greatly affected cell adhesion-related phenomena and cell–substrate crosstalk during corneal keratocyte cultivation.

Eid et al. studied the influence of pegylation and CS coating of ofloxacin lipid NPs [300]. Both the pegylation of lipid NPs and CS coating resulted in bigger NPs. Their shape was nearly spherical, with a smooth surface. Both pegylation and the CS coating resulted in a two- to threefold increase in the amount of ofloxacin that could be delivered to ocular fluids and tissues compared to commercial Oflox^@^ drops. Ban et al. reported the CS coating of dexamethasone-containing lipid NPs [301]. As a result of CS coating, the ζ-potential shifted from negative to positive values, and a higher permeation was observed. The lipid NPs exhibited a higher bioavailability compared to dexamethasone aqueous solution. Gelfuso et al. studied the use of iontophoresis as a method to increase voriconazole release, leading to an enhanced initial burst effect [302]. In brief, three different formulations were used: a cyclodextrin inclusion complex, a liposomal NP, and a CS-coated liposomal NP. After applying iontophoresis for 10 min, voriconazole penetration into the cornea was enhanced in all three formulations, with uncoated liposomal NPs showing the lowest concentration of the drug.

Seyfoddin et al. used a blend of different esters of behenic acid with glycerol (commercially available as Compritol^®^ 888 ATO) as a lipid carrier for the nanoencapsulation of acyclovir, which is a drug that is used in the therapy of herpes keratitis: the most common infectious cause of blindness [303]. NPs were formed via the “hot microemulsion technique” and coated thereafter with CS, via their dispersion in solutions of different CS concentrations. The coating was achieved due to electrostatic interactions between the negatively charged lipid carrier and the positively charged CS. The authors studied the influence of the lyophilization process on the resulting NPs. It was observed that NPs tended to aggregate after freeze drying, resulting in higher sizes reaching the micro-scale range. Freshly prepared coated NPs showed small particle sizes between 323 and 468 nm; the size increased with increasing CS concentration. The ζ-potential was also proportional to the increase of CS concentration, starting from about −26 mV for uncoated NPs to about +28 mV when 1% *w/v* CS was used. No significant difference was observed between CS concentrations of 0.5% and 1% *w/v*. Drug release was studied in PBS pH 7.4. It was found that coating with CS led to a reduction of about 25% in acyclovir release rate compared to uncoated NPs, regardless of the CS concentration used for coating. The cellular uptake of NPs was enhanced by CS coating while the concentration of fluorescein increased with CS concentration and exposure time in the cytoplasm of the epithelial cells. In ex vivo penetration studies, conducted in bovine eyes, the coated NPs had enhanced properties, with the coating obtained from the 0.5% *w/v* CS concentration showing the best results. The quantification of acyclovir in cellular uptake was higher for the coated NPs.

Selvaraj et al. designed CS-coated NLCs for the ocular delivery of itraconazole [304]. The CS coating delayed itraconazole release due to the formation of a hydrophilic matrix around the lipid carriers, and ex vivo corneal permeability was significantly enhanced. In the antineovascularization study, CS-coated NLCs demonstrated a high reduction in neovascularization, which was attributed to low pre-corneal drainage, due to the higher mocoadhesivity and higher corneal permeation. Wang et al. used glyceryl monostearate as the solid lipid in the NP formulation of methazolamide used in glaucoma treatment [305]. Phospholipid was used as the surfactant in a modified oil-in-water emulsification technique, while CS was used for the coating of NPs. The results are in accordance with other studies; i.e., a smooth surface increased the particle size compared to uncoated NPs and prolonged drug release. In vivo results showed that coated NPs successfully delivered methazolamide in rabbit eyes, showing a marked decrease in IOP and a better sustainability than the uncoated ones.

Dukovski et al. investigated the performance of a cationic nanoemulsion containing ibuprofen for the treatment of dry eye disease, in order to resolve ibuprofen solubility complications and stabilize the tear film [306]. Nanoemulsions were prepared by microfluidization, using lecithin as an anionic surfactant, Miglyol 812 as an internal oil phase, Kolliphor EL as the second surfactant, and CS. CS was expected to depose on the surface of the nanoemulsion droplets, increasing the ζ-potential and thus mucoadhesion. The mucoadhesive properties of the obtained formulations were tested rheologically after mixing with mucin dispersion, and indeed, a significantly increased mucoadhesion was observed for the CS-coated nanoemulsion. The formulation further demonstrated good compatibility and stability. Nanoemulsions with 0.05% *w/w* CS exhibited the best characteristics and were found to be adequate for ophthalmic applications.

Another group that worked on liposomes coated with CS is Zhang et al. [307]. The group prepared liposomes via the reverse-phase evaporation method, which were subsequently coated with TMCS, **1** in Scheme 1. Cyanidin-3-glucoside was encapsulated in the liposomes for the treatment of cataract. The uncoated liposomes were negatively charged while a modified CS coating made their surface positively charged and simulteneously enlarged their size. Furthermore, the coating protected the drug from oxidative damage. The presence of TMCS increased the pre-corneal retention time, which was triplicated compared to uncoated liposomes. Moreover, it enhanced the permeation and the transepithelial transport of liposomes, as it opened the tight junctions between the epithelial cells. Regarding the release of the drug, a slower release and diffusion of the drug was observed owing to the presence of the coating. Huang et al. also used TMCS to coat lanosterol and hesperetin-loaded liposomes for cataract prevention and treatment [308]. The coated liposomes exhibited a slightly decreased encapsulation efficiency compared to the uncoated one. Most importantly, in in vivo studies, they demonstrated a smaller burst release and a slower overall release, extending over one week. Despite their lower drug load, the coated liposomes had a better therapeutic efficacy with no progression of cataract observed for the subjects treated with TMCS-coated liposomes.

Pai et al. used CS oligosaccharide in order to coat NLCs for the delivery of etoposide, which is an antineoplastic agent used for suppressing tumors that occur in the eye such as retinoblastoma [309]. NLCs were prepared via a hot homogenization–ultrasonication technique, and the resulting NPs were coated by CS oligosaccharides. Typical findings were also verified in the present study, i.e., an increase of NP size after CS coating (from 103 to 117 nm) and the conversion of negatively charged NPs to positively charged ones. However, compared to other analogous formulations, it was found that the release profile of the drug, conducted in STF, was not affected by CS oligosaccharide coating, which was probably due to the form of CS used, i.e., oligosaccharides. The mucoadhesion properties of coated NPs were enhanced compared to the negligible mucoadhesion of non-coated NPs. Mucoadhesion prolonged the presence of NPs on the ocular surface, as revealed by in vivo studies, leading to an enhanced ocular concentration of etoposide in all parts of the eye.

**Figure 22:**
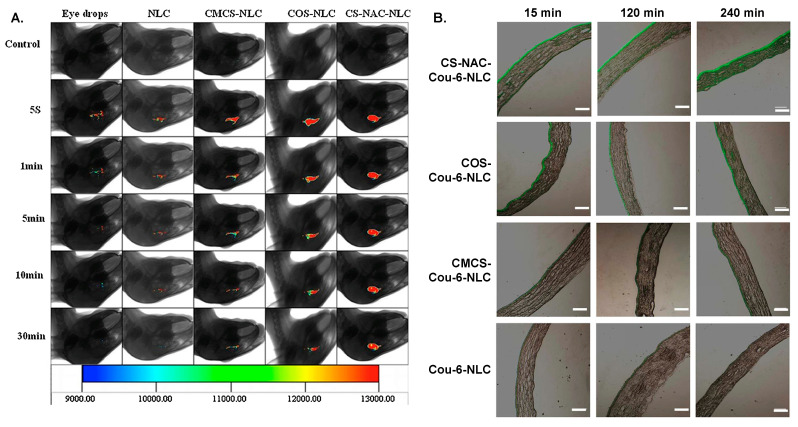
(**A**) In vivo fluorescence imaging of curcumin (CUR) in eye drops, nanostructured lipid carriers (NLC), carboxymethyl chitosan-coated NLC (CMCS-NLC), chitosan oligosaccharides-coated NLC (COS-NLC) and N-acetylcysteine chitosan-coated NLC (CS-NAC-NLC) at 5 s, 1 min, 5 min, 10 min, and 30 min after administration. Reprinted from ref. [310]. Copyright 2016 with permission from Elsevier Ltd. (**B**) Inverted fluorescence microscope micrographs after time-coursed in vivo corneal permeation of the aforementioned preparations, loaded with coumarin. Scale bar is 150 μm. Adapted from ref. [312]. Copyright 2017, with permission from Elsevier B.V.

Li et al. studied three different CS derivatives—*N*-acetylcysteine CS (NACCS), **6** in Scheme 1, CS oligosaccharides, and carboxymethyl CS—for the coating of curcumin-loaded NLC [310]. According to curcumin release studies, NACCS showed a remarkably slower drug release compared to the other two coatings, which was probably due to the formation of disulfide bonds within the coating, impeding the diffusion of the drug. CS oligosaccharides showed a faster release compared to the thiolated CS coating; however, in contrast to Pai et al. [309], it was still slower than uncoated NLC. Finally, carboxymethyl CS showed the fastest release of all the coated NPs. Concerning corneal permeation studies, it was found that in the first 60 min, no difference was observed among the three coated formulations, but thereafter, thiolated CS, i.e., NACCS, showed an enhanced corneal permeation due to the thiol groups that can penetrate the tight junctions of mucus layers. These observations were further confirmed by fluorescence imaging, which was used to evaluate in vivo pre-corneal retention (Figure 22A). The same team further investigated the influence of the thiol group content in NACCS, which was used as a coating for curcumin-loaded NLC [311]. The total content of thiol groups did not affect curcumin’s release despite differences in the size and ζ-potential. This was attributed to the similar entrapment efficiency observed for all three coatings. Differences appeared in ex vivo cornea penetration studies, which showed that after 60 min, the coating with the highest degree of thiolation resulted in higher cornea penetration. These outcomes were also confirmed by fluorescence imaging. A similar study was conducted for the ocular delivery of hydrophobic coumarin-6 instead of curcumin [312,313]. The results concerning both the coating with NACCS compared to CS oligosaccharides or carboxymethyl CS [312], as well as the content of thiol groups [313], were the same (Figure 22B).

Salama et al. used PLGA 50/50 or 75/25 in NP formulation of fluocinolone acetonide (FA), which is an anti-inflammatory corticosteroid drug used for the treatment of intermediate, posterior, and pan uveitis [314]. NPs were prepared by an innovative thin film hydration process using P407 in a mass ratio of PLGA/P407 1:5 and 1:10. The NPs prepared with PLGA 50/50 were smaller compared to the ones prepared with PLGA 75/25. This was attributed to the higher hydrophilicity of the former due to the higher glycolide content. This enabled the easier interfacial arrangement of PLGA molecules. Entrapment efficiency was higher for PLGA 75/25 NPs, which was due to the higher hydrophobic content of the polymer, which favored the encapsulation of the hydrophobic drug. The ζ-potential was negative for all prepared NPs due to the presence of terminal carboxylic acid groups in PLGA, and it increased with increasing poloxamer content. The authors selected the best formulation and proceeded in NPs coating using CS hydrochloride in different concentrations. It was observed, as it was by Seyfoddin et al. [303], that as the concentration of CS increased, the size of NPs increased from about 203 nm to about 2147 nm, while the ζ-potential also increased from negative to positive values. The important increase in size was attributed to agglomeration. Unfortunately, the isolation procedure of NPs was not described; it was probably by freeze drying, which could explain such sizes. The release profile revealed that the coated NPs showed improvements compared to the uncoated ones. In contrast to FA, which is known to cause irritation, an ocular irritation study conducted in rabbit eyes demonstrated that neither uncoated nor coated NPs induced any irritation. Furthermore, it was found that the concentration of FA in rabbits tears were higher when coated NPs were used. This was mainly attributed to the mucoadhesive properties of CS.

Pandit et al. studied CS-coated PLGA NPs for bevacizumab delivery [315]. Drug-loaded NPs were prepared by double emulsion solvent evaporation, experimental parameters were optimized by response surface methodology, and well-defined, spherical core–shell particles were obtained. CS coating contributed to a reduced initial burst release, a slower release compared to uncoated NPs, and a higher permeation. Khan et al. reported CS-coated PLGA NPs for the delivery of forskolin in glaucoma treatment [316]. CS-coated NPs were prepared in a single step by emulsion sonication. The experimental parameters were optimized using the Box–Behnken design. Increasing the CS concentration resulted in an increase in NPs’ size, a rise in PDI, and a decrease in drug loading, but an increase in entrapment efficiency. The optimized NPs were approximately 200 nm and had a positive ζ-potential. A slow release of forskolin was observed with no burst release. According to the authors, CS coating functioned as a physical barrier, restricting drug release. It is suggested that drug release was a result of CS swelling/erosion, followed by PLGA erosion and drug diffusion. Due to CS coating, the NPs were found to penetrate deeper in the cornea. Overall, CS-coated forskolin-loaded PLGA NPs showed a sustained IOP decrease for 24 h. Dyawanapelly et al. investigated the potential of CS oligosaccharide in the mucosal delivery of drug-loaded PLGA NPs [317]. In contrast to CS coating, CS oligosaccharide coating did not affect significantly drug release. Both coatings resulted in improved mucoadhesion and higher cellular uptake. Mahaling et al. studied the influence of different coatings on the bioavailability and distribution of NPs in the ocular region [318,319]. Initially, CS, gelatin, or pluronic F68 coatings of poly(ε-caprolactone) (PCL) NPs were studied, and the scope was then further extended to PLA and PLGA NPs. The NPs coated with F68 exhibited the highest hydrophilicity and mucoadhesivity. For PCL NPs, F68 was the best coating, but for PLGA NPs, CS-coated NPs demonstrated a higher bioavailability in conjunctiva, sclera, choroid, and retina. Nasr and Khoee reported the formation of a crosslinked CS shell, coating poly(butylene adipate) (PBA) micelles for the improved ocular delivery of loteprednol etabonate [320]. PBA was modified in order to prepare a dendrimerized structure with hydrophilic amine-functionalized chain ends that could form micelles. The amine-functionalized micelles were further reacted with *N*-succinyl modified CS, **2** in Scheme 1, in the presence of EDC and *N*-hydroxysuccinimide. CS was grafted to the micelles via the reaction of the amine groups of the micelles with the carboxylic acid groups of modified CS. Additionally, a crosslinking reaction among amine and carboxylic groups within CS chains took also place. Core–shell spherical NPs were obtained with sizes from 40 to 82 nm depending on the molecular weight of PBA. Commercial eye drops released their drug content within 10 h. In contrast, loteprednol etabonate release from the crosslinked core–shell NPs was extended over 50 h.

## 7. Concluding Remarks

CS is a versatile polymer due to its favorable biocompatibility, biodegradability, mucoadhesive, and penetration enhancement properties. Numerous applications and different drug delivery systems have been reported in the field of ocular drug delivery with promising results in terms of increased drug bioavailability and improved therapeutic effect. Not surprisingly, CS nanoparticles dominate amongst the different kind of delivery formulations. NPs have shown their superiority in drug delivery, generally speaking. In the challenging field of ocular delivery, the advantages of nanoparticles, such as their small size, high surface area, and high mucoadhesivity, are even more crucial. Ionotropic gelation, especially with TPP, is a convenient and easily accessible method to prepare CS nanoparticles and as such has attracted much attention. The importance of the experimental protocol, and more specifically the CS/TPP ratio and the CS and TPP concentrations, on the size, the dispersity, the ζ-potential (and thus stability), and the entrapment efficiency have been extensively discussed. Interesting performances have been obtained in terms of sustained release, and more are yet to come. In situ hydrogels are another type of formulation with encouraging results that have the advantage of being instilled as eye drops but that can form a hydrogel with a higher retention time once on the ocular surface. Innovative, more complex designs and architectures have started to emerge, combining complementary delivery vehicles; these need to be further investigated. These systems hold the promise of more efficient delivery as they combine multiple advantages. For example, NPs loaded in hydrogels or contact lenses can combine the controlled delivery of the NPs with a higher corneal retention time offered by the ocular lens or hydrogel, decreasing the necessity of frequent treatment. CS coating is another method to confer CS advantages to other matrices, without compromising their own advantages. The increase of therapeutic efficiency is the primary objective of any drug delivery system; nonetheless, patient’s comfort is almost as critical, since it heavily affects compliance to a treatment. This is especially true when it comes to ocular delivery. Thus, it should always be kept in sight when designing a drug delivery system.

## Figures and Tables

**Table 1:** Indicative experimental parameters used for the preparation of chitosan (CS) nanoparticles with sodium tripolyphosphate (TPP) and some of the characteristics of the obtained nanoparticles. PDI: polydispersity index.

CS/TPP Mass Ratio	Stirring Rate and Time	Size (nm)	ζ-Potential (mV)	PDI	Ref.
5/1	1000 rpm 90 s	214.7 ± 20.1	+21.79 ± 8.17	0.172 ± 0.022	[129]
4/1	100 rpm	342 ± 8	-	narrow, unimodal	[130]
4/1 ^a^	sonication 15 min	295.3 ± 3.0	+29.3 ± 1.8	0.185 ± 0.016	[131]
4/1 ^b^	sonication 15 min	336 ± 22.1	+22.5 ± 1.8	0.246 ± 0.07	[131]
11/3	300 rpm 10 min	81.9 ±10.1	-	0.244 ± 0.07	[132]
3/1	500 rpm 120 min	401.5 ± 14.3	+31.3 ± 3.5	0.198 ± 0.054	[133]
3/1	1000 rpm 2 h	172.3 ± 9.0	+36.46 ± 0.59	0.251 ± 0.001	[134]
2.5/1	1200 rpm 6 h	131.9 ±3.5 ^c^	-	0.406 ± 0.03 ^d^	[135]
		161.9 ±3.3 ^d^	+30.43 ± 1.0 ^d^	0.321 ± 0.054 ^d^	
2/1	1000 rpm 2 h	188.5 ± 8.5	+36.86 ± 0.70	0.22	[136]
2/1	800 rpm	287 ± 17	+37.59	0.78	[137]
1.875/1	-	147.2 ± 15.3	+19.4 ± 0.8	0.22 ± 0.04	[138]
1/1	- 18 h	350.6	+33.7	-	[139]
1/2	8000 rpm 20 min	57.0	+27.90	0.02	[140]

^a^: low molecular weight chitosan, ^b^: high molecular weight chitosan, ^c^: empty nanoparticles, ^d^: drug-loaded nanoparticles.

**Table 2:** Optimized chitosan (CS) nanoparticles obtained with other polyanions apart from sodium tripolyphosphate.

Combined Polyanion	CS/Polyanion Mass Ratio	Size (nm)	ζ-Potential (mV)	PDI	Drug	Ref.
Lecithin	1/10	161.9 ± 1.3	+26.6 ± 1.13	0.189 ± 0.004	Amphotericin B	[149]
Lecithin	1/20	241.8 ± 0.8	+22.7 ± 0.7	0.207 ± 0.003	Melatonin	[166]
Pectin	1/2	229.02 ± 0.45	+26.6 ± 0.04	0.45 ± 0.04	Brinzolamide	[167]
Pectin, calcium chloride	1/80	274.9 ± 14.45	-	0.634 ± 0.066	Timolol maleate	[168]
Sodium alginate	1/10	80–100	-	0.020 ± 0.008	Timolol maleate	[169]
TPP and sodium alginate	1/0.086/0.029 ^a^	439.8 ± 1.9	+37.4 ± 1.7	0.39 ± 0.14	Ofloxacin	[126]
Calcium alginate	1/1	310.2	+28.9	0.353	Rosiglitazone	[171]
Carboxymethyl gum katira	-	269	-	0.236	Ofloxacin	[172]
Flax seed gum	-	267.06 ± 8.65	−20.3 ± 2.88	0.345 ± 0.02	Timolol maleate	[173]
Dextran sulfate EDC	1/1.6	454 ± 7	+34.4 ± 3.1	0.28 ± 0.01	Lutein	[174]
Dextran sulfate	1/0.13	172.3 ± 9.03	+36.46 ± 0.59	0.257 ± 0.015	Acetazolamide	[175]
Dextran sulfate	1/0.13	182.63 ± 4.64	+43.03 ± 0.51	0.259 ± 0.014	Dorzolamide	[134]
Dextran sulfate	4/3	~350	+3.55	-	Ciprofloxacin	[176]
Hyaluronic acid TPP	1/4.6 ^b^	362 ± 35	+44 ± 1	0.14 ± 0.00	Ceftazidime	[8]
Sulfobutylether-β-cyclodextrin	1/0.3	446.4 ± 113	+22.5 ± 4.91	-	Naringenin	[179]
Sodium deoxycholate	1/5	321 ± 22	+28.8	0.454	Prednisolone acetate	[180]
Lysine	1/2	289.6 ± 2.48	+20.19 ± 1.11	0.32 ± 0.04	Itraconazole	[182]

EDC: 1-ethyl-3-(3-dimethylaminopropyl) carbodiimide hydrochloride, ^a^: CS/TPP/sodium alginate ratioc, ^b^: CS/total anion ratio.

**Table 3:** Nanoparticles with modified chitosan (CS) and sodium tripolyphosphate (TPP). API: active pharmaceutical ingredient.

Modified CS Derivative	Modified CS/TPP Mass Ratio	Size (nm)	ζ-Potential (mV)	PDI	API	Ref.
N-trimethyl CS	3/1	155	+8.3	0.2	Diclofenac sodium	[189]
N-trimethyl CS	6/1	165.8 ± 9.89	+18 ± 0.57	0.149 ± 0.01	Flurbiprofen	[190]
N-carboxybenzyl CS	7/1	190	-	-	Timolol maleate	[191]
methylmethacrylate CS (CS/MMA mol ratio 1:2.5)	1/1	200	-	3.2	Curcumin	[194]
CS-g-PEGMA	1/2 ^a^	500	0.583	-	Bevacizumab	[197]
Aminated CS	1/1	175.2 ± 0.99	+42.4	0.217	Curcumin	[200]
Galactosylated CS	5.25	223.3 ± 6.8	30.2 ± 0.46	0.3	Timolol maleate	[201]

PEGMA: poly(ethylene glycol) methacrylate, ^a^: secondary crosslinking with glutaraldehyde.

**Table 4:** Thermoresponsive chitosan (CS)/poloxamer (P407) hydrogels.

CS	P407	Other Polymers	T_gel_ (°C)	T_gel_ after Dilution with STF (°C)	t_gel_	Drug	Ref.
0.3% *w/w*	15% *w/w*	-	33.3	35.4	-	Bupivacaine hydrochloride	[235]
0.25% *w/w*	14.2% *w/w*	P188 1.7% *w/w*	31.1	33.5	41.1–87.4 s	4 APIs	[236]
0.2% *w/w*	25% *w/w*	-	37	-	immediate	Neomycin sulfate and Betamethasone sodium phosphate	[237]
1% *w/w*	20% *w/w*		19.7	28.7	-	-	[238]
0.5%	16%	HPMC 0.5% *w/v*	-	-	46 min	Ketoconazole-loaded PLGA NPs	[239]
1% *w/w*	16% *w/w*	-	31–32	-	-	Nepafenac in silica NPs	[240]
1% *w/v*	16% *w/v*	-	32	-	-	Insulin in CS microparticles	[241]

HPMC: hydroxypropyl methycellulose, PLGA: poly(lactic-*co*-glycolic acid).

**Table 5:** Chitosan (CS)-gelatin hydrogels.

CS	Gelatin	Crosslinking Agent	T_gel_ (°C)	t_gel_ (s)	Drug	Ref.
2% *w/v*	0.2% *w/v*	β-GP	34.2 ^a^	70.7 ^a^	Latanoprost	[246]
1% *w/w*	3% *w/w*	EDC 1% *w/v*	-	-	Fluorescein	[249]
2.5% *w/v*	1% *w/v*	β-GP 0.8% *w/v* & genipin (100 μg/mL)	37	30–35 (from 25 °C)	Timolol maleate	[250]
1% *w/v*	0.5% *w/v*	Oxidized sucrose 0.15% *w/v*	-	-	Timolol maleate	[251]

β-GP: β-glycerolphosphate, EDC: 1-ethyl-3-(3-dimethylaminopropyl) carbodiimide hydrochloride. ^a^: latanoprost-loaded.

**Table 6:** Chitosan (CS) hydrogels with other polysaccharides.

CS	Other Polymers	Crosslinking Agent	Trigger	t_gel_	Drug	Ref.
0.5% *w/w*	Sodium alginate 0.2% *w/w*		pH, Ca^2+^ (STF)		Levofloxacin	[255]
0.5%	Sodium alginate 1%	HPMC 0.5% *w/v*	Ca^2+^ (STF)	47 min	Ketoconazole-loaded PLGA NPs	[239]
0.5%	Gellan gum 0.2%		pH, Ca^2+^ (STF)		Sparfloxacin	[257]
0.5% *w/v*	Gellan gum0.25 *w/v*	PV A2% *w/v*	pH 7.4 (STF)	immediate	Besifloxacin	[258]
1% *w/v*	Odex 2% *w/v*		-^a^	2.5 min	Retinal progenitor cells	[259]
3%	HPMC 4%		- ^a^		Fluconazole	[260]

STF: simulated tear fluid, HPMC: hydroxypropyl methylcellulose, PLGA: poly(lactic-co-glycolic acid), NPs: nanoparticles, PVA: poly(vinyl alcohol). ^a^: not in situ gelling systems.

**Table 7:** Covalently cross-linked modified chitosan (CS) hydrogels.

Modified CS	Crosslinking Agent	Volume Ratio	Gel Formation	t_gel_	Drug	Ref.
CMCS 4% *w/w*	Oxidized dextran 4% *w/w*	6:7	Mixing rt	18 s	Voriconazole	[261]
CMCS 20 mg/mL ^a^	Oxidized sodium alginate 20 mg/mL ^a^	1:1	Mixing rt	40–60 s	Limbal stem cells	[262]
GlyCS	4-arm PEG CHO end-chains	20:100 ^b^	Mixing rt	30 s	Levofloxacin	[263]
Azide-modified GlyCS 3% *w/v* ^a^	-	-	UV	30 s	Heparin	[264]
L-cysteine modified CS 150 mg/mL	-	-	Air oxidation	Several hours	Ranibizumab Aflibercept	[265]

CMCS: carboxymethyl CS, rt: room temperature, PEG: poly(ethylene glycol), GlyCS: glycol-modified CS. ^a^: in 0.9% NaCl aqueous solution. ^b^: mass ratio.

**Table 8:** In situ gelling formulations from modified chitosans (CS).

Modified CS	Crosslinking Agent	2nd Polymer	Trigger	T_gel_ (°C)	Drug	Ref.
CS-g-PNIPAAm ^a^	-	-	T	31.9	Pilocarpine	[266]
CS-g-PNIPAAm ^b^	-	-	T	29.7	Pilocarpine	[267]
Quaternary ammonium CS 20%	β-GP	CS	T	35	5-fluorouracil CS NPss	[178]
CS **24** ^c^ 3.8% *w/w*	β-GP 20% *w/w*	-	T	35	Dexamethasone- loaded NLC	[269]
HexGlyCS ^d^ 2% *w/w*	-	-	T	30	Brimonidine tartrate	[270]
CMCS 2% *w/v*	β-GP 7.8% *w/v*	CS 2% *w/v*	T		-	[153]
CMCS 2.5% *w/v*	GA	P407 5.0% *w/v*	pH, T	32–33	Nepafenac	[273]
CMCS 3% *w/v*	genipin	P407 1% *w/v*	pH, T	37	Baicalin-loaded NLC	[276]
Hydroxybutyl CS 8 mg/mL	-	-	T	37	-	[277]

PNIPAAm: poly(*N*-isopropylacrylamide), β-GP: β-glycerolphosphate, NPs: nanoparticles, NLC: nanostructured lipid carriers, HexGlyCS: hexanoyl glycol chitosan, CMCS: carboxymethyl chitosan, GA: glutaraldehyde, P407: poloxamer 407. ^a^: degree 74%, grafting ratio 413, ^b^: deacetylation degree 80%, grafting ratio 161, ^c^: hydroxypropyltrimethyl ammonium chloride CS, ^d^: degree of hexanoylation 39.5%.

## Abbreviations

AA	acrylic acid
ALG	sodium alginate
API	active pharmaceutical ingredient
CMC	carboxymethyl cellulose
CMCS	*O*-carboxymethyl chitosan
CS	chitosan
CS-*g*-PN	Chitosan-grafted poly(*N*-isopropylacrylamide)
DEMC	*N*-diethylmethyl chitosan
Dex-GlyCS	dexamethasone glycol-chitosan conjugates
DexS	dextran sulfate
DMEC	dimethylethyl chitosan
EDC	1-Ethyl-3-(3-dimethylaminopropyl) carbodiimide
FA	fluocinolone acetonide
FTIR	Fourier transformation infra-red spectroscopy
GalCS	galactosylated chitosan
GlyCS	glycol chitosan
HA	hyaluronic acid
HexGlyCS	hexanoyl glycol chitosan
HMWCS	high molecular weight chitosan
HPMC	hydroxypropyl methylcellulose
IOP	intraocular pressure
L	lecithin
LCST	lower critical transition temperature
LMWCS	low molecular weight chitosan
NACCS	*N*-acetylcysteine chitosan
NLCs	nanostructured lipid carriers
NPs	nanoparticles
OCT	optical coherence tomography
Odex	oxidized dextran
P407	poloxamer 407
PAA	poly(acrylic acid)
PBA	poly(butylene adipate)
PBS	phosphate-buffered saline
PCL	poly(ε-caprolactone)
PDI	polydispersity index
PEG	poly(ethylene glycol)
PGGA	poly(gamma-glutamic acid)
PHEMA	poly(2-hydroxyethylmethacrylate)
PLA	poly(lactide)
PLGA	poly(lactic-*co*-glycolic acid)
PNIPAAm	poly(*N*-isopropylacrylamide)
PPO	poly(propylene oxide)
PVA	poly(vinyl alcohol)
PVP	poly(vinyl pyrrolidone)
SBE-*β*-CD	sulfobutylether-*β*-cyclodextrin
SEM	scanning electron microscopy
STF	simulated tear fluid
TECS	triethyl chitosan
TEM	transmission electron microscopy
*T_gel_*	temperature of gelation
TMCS	trimethyl chitosan
TPP	sodium tripolyphosphate
β-GP	β-glycerolphosphate, glycerol phosphate disodium salt

## References

[1] Sánchez-López E., Espina M., Doktorovova S., Souto E.B., García M.L. (2017). Lipid nanoparticles (SLN, NLC): Overcoming the anatomical and physiological barriers of the eye—Part I—Barriers and determining factors in ocular delivery. Eur. J. Pharm. Biopharm..

[2] Jumelle C., Gholizadeh S., Annabi N., Dana R. (2020). Advances and limitations of drug delivery systems formulated as eye drops. J. Control. Release.

[3] Souto E.B., Dias-Ferreira J., López-Machado A., Ettcheto M., Cano A., Espuny A.C., Espina M., Garcia M.L., Sánchez-López E. (2019). Advanced formulation approaches for ocular drug delivery: State-of-the-art and recent patents. Pharmaceutics.

[4] Urtti A. (2006). Challenges and obstacles of ocular pharmacokinetics and drug delivery. Adv. Drug Deliv. Rev..

[5] Gaudana R., Ananthula H.K., Parenky A., Mitra A.K. (2010). Ocular drug delivery. AAPS J..

[6] Alvarez-Lorenzo C., Yañez F., Concheiro A. (2010). Ocular drug delivery from molecularly-imprinted contact lenses. J. Drug Deliv. Sci. Technol..

[7] Patel A., Cholkar K., Agrahari V., Mitra A.K. (2013). Ocular drug delivery systems: An overview. World J. Pharmacol..

[8] Silva M.M., Calado R., Marto J., Bettencourt A., Almeida J., Gonçalves L.M.D. (2017). Chitosan nanoparticles as a mucoadhesive drug delivery system for ocular administration. Mar. Drugs.

[9] Gaudana R., Jwala J., Boddu S.H.S., Mitra A.K. (2009). Recent perspectives in ocular drug delivery. Pharm. Res..

[10] Achouri D., Alhanout K., Piccerelle P., Andrieu V. (2013). Recent advances in ocular drug delivery. Drug Dev. Ind. Pharm..

[11] Duxfield L., Sultana R., Wang R., Englebretsen V., Deo S., Rupenthal I.D., Al-Kassas R. (2016). Ocular delivery systems for topical application of anti-infective agents. Drug Dev. Ind. Pharm..

[12] Khare A., Grover K., Pawar P., Singh I. (2014). Mucoadhesive polymers for enhancing retention in ocular drug delivery: A critical review. Rev. Adhes. Adhes..

[13] White C.J., Tieppo A., Byrne M.E. (2011). Controlled drug release from contact lenses: A comprehensive review from 1965-present. J. Drug Deliv. Sci. Technol..

[14] Fulgêncio G., De O., Viana F.A.B., Ribeiro R.R., Yoshida M.I., Faraco A.G., Da Silva Cunha-Júnior A. (2012). New mucoadhesive chitosan film for ophthalmic drug delivery of timolol maleate: In vivo evaluation. J. Ocul. Pharmacol. Ther..

[15] Bachu R.D., Chowdhury P., Al-Saedi Z.H.F., Karla P.K., Boddu S.H.S. (2018). Ocular drug delivery barriers—Role of nanocarriers in the treatment of anterior segment ocular diseases. Pharmaceutics.

[16] Huang D., Chen Y.S., Rupenthal I.D. (2018). Overcoming ocular drug delivery barriers through the use of physical forces. Adv. Drug Deliv. Rev..

[17] Gote V., Sikder S., Sicotte J., Pal D. (2019). Ocular drug delivery: Present innovations and future challenges. J. Pharmacol. Exp. Ther..

[18] Cao Y., Samy K.E., Bernards D.A., Desai T.A. (2019). Recent advances in intraocular sustained—Release drug delivery devices. Drug Discov. Today.

[19] Agban Y., Thakur S.S., Mugisho O.O., Rupenthal I.D. (2019). Depot formulations to sustain periocular drug delivery to the posterior eye segment. Drug Discov. Today.

[20] Maharjan P., Cho K.H., Maharjan A., Shin M.C., Moon C., Min K.A. (2019). Pharmaceutical challenges and perspectives in developing ophthalmic drug formulations. J. Pharm. Investig..

[21] Bhattacharjee A., Das P.J., Adhikari P., Marbaniang D., Pal P., Ray S., Mazumder B. (2019). Novel drug delivery systems for ocular therapy: With special reference to liposomal ocular delivery. Eur. J. Ophthalmol..

[22] Suri R., Beg S., Kohli K. (2020). Target strategies for drug delivery bypassing ocular barriers. J. Drug Deliv. Sci. Technol..

[23] Ntohogian S., Gavriliadou V., Christodoulou E., Nanaki S., Lykidou S., Naidis P., Mischopoulou L., Barmpalexis P., Nikolaidis N., Bikiaris D.N. (2018). Chitosan nanoparticles with encapsulated natural and Uf-purified annatto and saffron for the preparation of UV protective cosmetic emulsions. Molecules.

[24] Tashakori-Sabzevar F., Mohajeri S.A. (2015). Development of ocular drug delivery systems using molecularly imprinted soft contact lenses. Drug Dev. Ind. Pharm..

[25] Kumar A., Vimal A., Kumar A. (2016). Why chitosan? From properties to perspective of mucosal drug delivery. Int. J. Biol. Macromol..

[26] Li J., Cai C., Li J., Li J., Li J., Sun T., Wang L., Wu H., Yu G. (2018). Chitosan-based nanomaterials for drug delivery. Molecules.

[27] Mohammed M.A., Syeda J.T.M., Wasan K.M., Wasan E.K. (2017). An overview of chitosan nanoparticles and its application in non-parenteral drug delivery. Pharmaceutics.

[28] Green S., Roldo M., Douroumis D., Bouropoulos N., Lamprou D., Fatouros D.G. (2009). Chitosan derivatives alter release profiles of model compounds from calcium phosphate implants. Carbohydr. Res..

[29] Kean T., Thanou M. (2010). Biodegradation, biodistribution and toxicity of chitosan. Adv. Drug Deliv. Rev..

[30] Mendes A.C., Moreno J.S., Hanif M., Douglas T.E.L., Chen M., Chronakis I.S. (2018). Morphological, mechanical and mucoadhesive properties of electrospun chitosan/phospholipid hybrid nanofibers. Int. J. Mol. Sci..

[31] Hafezi F., Scoutaris N., Douroumis D., Boateng J. (2019). 3D printed chitosan dressing crosslinked with genipin for potential healing of chronic wounds. Int. J. Pharm..

[32] Michailidou G., Ainali N.M., Xanthopoulou E., Nanaki S., Kostoglou M., Koukaras E.N., Bikiaris D.N. (2020). Effect of poly(vinyl alcohol) on nanoencapsulation of budesonide in chitosan nanoparticles via ionic gelation and its improved bioavailability. Polymers.

[33] Roy S., Pal K., Anis A., Pramanik K., Prabhakar B. (2009). Polymers in mucoadhesive drug-delivery systems: A brief note. Des. Monomers Polym..

[34] Shaikh R., Raj Singh T., Garland M., Woolfson A., Donnelly R. (2011). Mucoadhesive drug delivery systems. J. Pharm. Bioallied Sci..

[35] Ensign L.M., Cone R., Hanes J. (2012). Oral drug delivery with polymeric nanoparticles: The gastrointestinal mucus barriers. Adv. Drug Deliv. Rev..

[36] Boegh M., Nielsen H.M. (2015). Mucus as a barrier to drug delivery—Understanding and mimicking the barrier properties. Basic Clin. Pharmacol. Toxicol..

[37] Peppas N.A., Buri P.A. (1985). Surface, interfacial and molecular aspects of polymer bioadhesion on soft tissues. J. Control. Release.

[38] Mikos A.G., Peppas N.A., Lenaerts V.M., Gurny R. (1990). Scaling concepts and molecular theories of adhesion of synthetic polymers to glycoproteinic networks. Bioadhesive Drug Delivery Systems.

[39] Khutoryanskiy V.V. (2011). Advances in mucoadhesion and mucoadhesive polymers. Macromol. Biosci..

[40] Bassi Da Silva J., Ferreira S.B. (2017). de S.; de Freitas, O.; Bruschi, M.L. A critical review about methodologies for the analysis of mucoadhesive properties of drug delivery systems. Drug Dev. Ind. Pharm..

[41] Grabovac V., Guggi D., Bernkop-Schnürch A. (2005). Comparison of the mucoadhesive properties of various polymers. Adv. Drug Deliv. Rev..

[42] Andrews G.P., Laverty T.P., Jones D.S. (2009). Mucoadhesive polymeric platforms for controlled drug delivery. Eur. J. Pharm. Biopharm..

[43] Bagan J., Paderni C., Termine N., Campisi G., Russo L.L., Compilato D., Di Fede O. (2012). Mucoadhesive polymers for oral transmucosal drug delivery: A review. Curr. Pharm. Des..

[44] Kellaway I.W., Gurny R., Junginger H.E. (1990). In vitro test methods for the measurement of mucoadhesion. Bioadhesion Possibilities and Future Trends (APV, Band 25).

[45] Peppas N.A., Sahlin J.J. (1996). Hydrogels as mucoadhesive and bioadhesive materials: A review. Biomaterials.

[46] Eliyahu S., Aharon A., Bianco-Peled H. (2018). Acrylated chitosan nanoparticles with enhanced mucoadhesion. Polymers.

[47] Van Der Lubben I.M., Verhoef J.C., Van Aelst A.C., Borchard G., Junginger H.E. (2001). Chitosan microparticles for oral vaccination: Preparation, characterization and preliminary in vivo uptake studies in murine Peyer’s patches. Biomaterials.

[48] Mahmood A., Lanthaler M., Laffleur F., Huck C.W., Bernkop-Schnürch A. (2017). Thiolated chitosan micelles: Highly mucoadhesive drug carriers. Carbohydr. Polym..

[49] Pontillo A.R.N., Detsi A. (2019). Nanoparticles for ocular drug delivery: Modified and non-modified chitosan as a promising biocompatible carrier. Nanomedicine.

[50] Collado-González M., González Espinosa Y., Goycoolea F.M. (2019). Interaction between chitosan and mucin: Fundamentals and applications. Biomimetics.

[51] Lohani A., Chaudhary G. (2012). Mucoadhesive microspheres: A novel approach to increase gastroretention. Chronicles Young Sci..

[52] Barbu E., Verestiuc L., Nevell T.G., Tsibouklis J. (2006). Polymeric materials for ophthalmic drug delivery: Trends and perspectives. J. Mater. Chem..

[53] Meng-Lund E., Muff-Westergaard C., Sander C., Madelung P., Jacobsen J. (2014). A mechanistic based approach for enhancing buccal mucoadhesion of chitosan. Int. J. Pharm..

[54] Sogias I.A., Williams A.C., Khutoryanskiy V.V. (2008). Why is chitosan mucoadhesive?. Biomacromolecules.

[55] Sogias I.A., Williams A.C., Khutoryanskiy V.V. (2012). Chitosan-based mucoadhesive tablets for oral delivery of ibuprofen. Int. J. Pharm..

[56] Nafee N.A., Boraie N.A., Ismail F.A., Mortada L.M. (2003). Design and characterization of mucoadhesive buccal patches containing cetylpyridinium chloride. Acta Pharm..

[57] Karavas E., Georgarakis E., Bikiaris D. (2006). Application of PVP/HPMC miscible blends with enhanced mucoadhesive properties for adjusting drug release in predictable pulsatile chronotherapeutics. Eur. J. Pharm. Biopharm..

[58] Lehr C.M., Bouwstra J.A., Schacht E.H., Junginger H.E. (1992). In vitro evaluation of mucoadhesive properties of chitosan and some other natural polymers. Int. J. Pharm..

[59] Shojaei A.H., Paulson J., Honary S. (2000). Evaluation of poly(acrylic acid-co-ethylhexyl acrylate) films for mucoadhesive transbuccal drug delivery: Factors affecting the force of mucoadhesion. J. Control. Release.

[60] De Sá L.L.F., Nogueira N.C., Filho E.C.D.S., Figueiras A., Veiga F., Nunes L.C.C., Soares-Sobrinho J.L. (2018). Design of buccal mucoadhesive tablets: Understanding and development. J. Appl. Pharm. Sci..

[61] Bartkowiak A., Rojewska M., Hyla K., Zembrzuska J., Prochaska K. (2018). Surface and swelling properties of mucoadhesive blends and their ability to release fluconazole in a mucin environment. Colloids Surf. B.

[62] Nafee N.A., Ismail F.A., Boraie N.A., Mortada L.M. (2004). Mucoadhesive delivery systems. I. Evaluation of mucoadhesive polymers for buccal tablet formulation. Drug Dev. Ind. Pharm..

[63] Abu-Huwaij R., Obaidat R.M., Sweidan K., Al-Hiari Y. (2011). Formulation and in vitro evaluation of xanthan gum or carbopol 934-based mucoadhesive patches, loaded with nicotine. AAPS PharmSciTech.

[64] Chopra S., Mahdi S., Kaur J., Iqbal Z., Talegaonkar S., Ahmad F.J. (2006). Advances and potential applications of chitosan derivatives as mucoadhesive biomaterials in modern drug delivery. J. Pharm. Pharmacol..

[65] Bhavsar C., Momin M., Gharat S., Omri A. (2017). Functionalized and graft copolymers of chitosan and its pharmaceutical applications. Expert Opin. Drug Deliv..

[66] Ways T.M.M., Lau W.M., Khutoryanskiy V.V. (2018). Chitosan and its derivatives for application in mucoadhesive drug delivery systems. Polymers.

[67] Greaves J.L., Wilson C.G. (1993). Treatment of diseases of the eye with mucoadhesive delivery systems. Adv. Drug Deliv. Rev..

[68] Muzzarelli R.A.A., Tanfani F. (1985). The N-permethylation of chitosan and the preparation of N-trimethyl chitosan iodide. Carbohydr. Polym..

[69] Karavasili C., Katsamenis O.L., Bouropoulos N., Nazar H., Thurner P.J., van der Merwe S.M., Fatouros D.G. (2014). Preparation and characterization of bioadhesive microparticles comprised of low degree of quaternization trimethylated chitosan for nasal administration: Effect of concentration and molecular weight. Langmuir.

[70] Jayakumar R., Prabaharan M., Nair S.V., Tokura S., Tamura H., Selvamurugan N. (2010). Novel carboxymethyl derivatives of chitin and chitosan materials and their biomedical applications. Prog. Mater. Sci..

[71] Upadhyaya L., Singh J., Agarwal V., Tewari R.P. (2014). The implications of recent advances in carboxymethyl chitosan based targeted drug delivery and tissue engineering applications. J. Control. Release.

[72] Aggarwal S., Agarwal S. (2015). Mucoadhesive polymeric platform for drug delivery: A comprehensive review. Curr. Drug Deliv..

[73] Hunt G., Kearney P., Kellaway I.W., Johnson P., Lloyd Jones J., Ellis H. (1987). Mucoadhesive polymers in drug delivery systems. Drug Delivery Systems: Fundamentals and Techniques.

[74] Bernkop-Schnürch A., Steininger S. (2000). Synthesis and characterisation of mucoadhesive thiolated polymers. Int. J. Pharm..

[75] Bernkop-Schnürch A., Scholler S., Biebel R.G. (2000). Development of controlled drug release systems based on thiolated polymers. J. Control. Release.

[76] Bernkop-Schnürch A., Hornof M., Guggi D. (2004). Thiolated chitosans. Eur. J. Pharm. Biopharm..

[77] Langoth N., Kahlbacher H., Schöffmann G., Schmerold I., Schuh M., Franz S., Kurka P., Bernkop-Schnürch A. (2006). Thiolated chitosans: Design and in vivo evaluation of a mucoadhesive buccal peptide drug delivery system. Pharm. Res..

[78] Kongsong M., Songsurang K., Sangvanich P., Siralertmukul K., Muangsin N. (2014). Design, synthesis, fabrication and in vitro evalution of mucoadhesive 5-amino-2-mercaptobenzimidazole chitosan as low water soluble drug carriers. Eur. J. Pharm. Biopharm..

[79] Cho I.S., Oh H.M., Cho M.O., Jang B.S., Cho J.-K., Park K.H., Kang S.-W., Huh K.M. (2018). Synthesis and characterization of thiolated hexanoyl glycol chitosan as a mucoadhesive thermogelling polymer. Biomater. Res..

[80] Nanaki S., Tseklima M., Christodoulou E., Triantafyllidis K., Kostoglou M., Bikiaris D.N. (2017). Thiolated chitosan masked polymeric microspheres with incorporated mesocellular silica foam (MCF) for intranasal delivery of paliperidone. Polymers.

[81] Bernkop-Schnürch A. (2005). Thiomers: A new generation of mucoadhesive polymers. Adv. Drug Deliv. Rev..

[82] Menzel C., Hauser M., Frey A., Jelkmann M., Laffleur F., Götzfried S.K., Gust R., Bernkop-Schnürch A. (2019). Covalently binding mucoadhesive polymers: N-hydroxysuccinimide grafted polyacrylates. Eur. J. Pharm. Biopharm..

[83] Eshel-Green T., Bianco-Peled H. (2016). Mucoadhesive acrylated block copolymers micelles for the delivery of hydrophobic drugs. Colloids Surf. B.

[84] Shitrit Y., Bianco-Peled H. (2017). Acrylated chitosan for mucoadhesive drug delivery systems. Int. J. Pharm..

[85] Ryu J.H., Choi J.S., Park E., Eom M.R., Jo S., Lee M.S., Kwon S.K., Lee H. (2020). Chitosan oral patches inspired by mussel adhesion. J. Control. Release.

[86] Kolawole O.M., Lau W.M., Khutoryanskiy V.V. (2018). Methacrylated chitosan as a polymer with enhanced mucoadhesive properties for transmucosal drug delivery. Int. J. Pharm..

[87] Bernkop-Schnürch A. (2005). Mucoadhesive systems in oral drug delivery. Drug Discov. Today Technol..

[88] Sigurdsson H.H., Kirch J., Lehr C.M. (2013). Mucus as a barrier to lipophilic drugs. Int. J. Pharm..

[89] Lehr C.M., Poelma F.G.J., Junginger H.E., Tukker J.J. (1991). An estimate of turnover time of intestinal mucus gel layer in the rat in situ loop. Int. J. Pharm..

[90] Lai S.K., Wang Y.Y., Hanes J. (2009). Mucus-penetrating nanoparticles for drug and gene delivery to mucosal tissues. Adv. Drug Deliv. Rev..

[91] Ponchel G., Montisci M.-J., Dembri A., Durrer C., Duchêne D. (1997). Mucoadhesion of colloidal particulate systems in the gastro-intestinal tract. Eur. J. Pharm. Biopharm..

[92] Zhang X., Cheng H., Dong W., Zhang M., Liu Q., Wang X., Guan J., Wu H., Mao S. (2018). Design and intestinal mucus penetration mechanism of core-shell nanocomplex. J. Control. Release.

[93] Rabea E.I., Badawy M.E.T., Stevens C.V., Smagghe G., Steurbaut W. (2003). Chitosan as antimicrobial agent: Applications and mode of action. Biomacromolecules.

[94] Tang H., Zhang P., Kieft T.L., Ryan S.J., Baker S.M., Wiesmann W.P., Rogelj S. (2010). Antibacterial action of a novel functionalized chitosan-arginine against Gram-negative bacteria. Acta Biomater..

[95] Matica M.A., Aachmann F.L., Tøndervik A., Sletta H., Ostafe V. (2019). Chitosan as a wound dressing starting material: Antimicrobial properties and mode of action. Int. J. Mol. Sci..

[96] Michailidou G., Christodoulou E., Nanaki S., Barmpalexis P., Karavas E., Vergkizi-Nikolakaki S., Bikiaris D.N. (2019). Super-hydrophilic and high strength polymeric foam dressings of modified chitosan blends for topical wound delivery of chloramphenicol. Carbohydr. Polym..

[97] Kong M., Chen X.G., Xing K., Park H.J. (2010). Antimicrobial properties of chitosan and mode of action: A state of the art review. Int. J. Food Microbiol..

[98] Kandimalla K.K., Borden E., Omtri R.S., Boyapati S.P., Smith M., Lebby K., Mulpuru M., Gadde M. (2013). Ability of chitosan gels to disrupt bacterial biofilms and their applications in the treatment of bacterial vaginosis. J. Pharm. Sci..

[99] Shariatinia Z. (2018). Carboxymethyl chitosan: Properties and biomedical applications. Int. J. Biol. Macromol..

[100] Rúnarsson Ö.V., Holappa J., Nevalainen T., Hjálmarsdóttir M., Järvinen T., Loftsson T., Einarsson J.M., Jónsdóttir S., Valdimarsdóttir M., Másson M. (2007). Antibacterial activity of methylated chitosan and chitooligomer derivatives: Synthesis and structure activity relationships. Eur. Polym. J..

[101] Sadeghi A.M.M., Amini M., Avadi M.R., Siedi F., Rafiee-Tehrani M., Junginger H.E. (2008). Synthesis, characterization, and antibacterial effects of trimethylated and triethylated 6-NH2-6-deoxy chitosan. J. Bioact. Compat. Polym..

[102] De Britto D., Celi Goy R., Campana Filho S.P., Assis O.B.G. (2011). Quaternary salts of chitosan: History, antimicrobial features, and prospects. Int. J. Carbohydr. Chem..

[103] Zhang J., Tan W., Luan F., Yin X., Dong F., Li Q., Guo Z. (2018). Synthesis of quaternary ammonium salts of chitosan bearing halogenated acetate for antifungal and antibacterial activities. Polymers.

[104] Sadeghi A.M.M., Dorkoosh F.A., Avadi M.R., Saadat P., Rafiee-Tehrani M., Junginger H.E. (2008). Preparation, characterization and antibacterial activities of chitosan, N-trimethyl chitosan (TMC) and N-diethylmethyl chitosan (DEMC) nanoparticles loaded with insulin using both the ionotropic gelation and polyelectrolyte complexation methods. Int. J. Pharm..

[105] Liu P., Meng W., Wang S., Sun Y., Aqeel Ashraf M. (2015). Quaternary ammonium salt of chitosan: Preparation and antimicrobial property for paper. Open Med..

[106] Xu T., Xin M., Li M., Huang H., Zhou S., Liu J. (2011). Synthesis, characterization, and antibacterial activity of N,O-quaternary ammonium chitosan. Carbohydr. Res..

[107] Jadhav R.L., Yadav A.V., Patil M.V. (2020). Poly Sulfoxyamine grafted chitosan as bactericidal dressing for wound healing. Asian J. Chem..

[108] De la Fuente M., Raviña M., Paolicelli P., Sanchez A., Seijo B., Alonso M.J. (2010). Chitosan-based nanostructures: A delivery platform for ocular therapeutics. Adv. Drug Deliv. Rev..

[109] Eljarrat-Binstock E., Orucov F., Aldouby Y., Frucht-Pery J., Domb A.J. (2008). Charged nanoparticles delivery to the eye using hydrogel iontophoresis. J. Control. Release.

[110] Nagarwal R.C., Kant S., Singh P.N., Maiti P., Pandit J.K. (2009). Polymeric nanoparticulate system: A potential approach for ocular drug delivery. J. Control. Release.

[111] De Campos A.M., Sánchez A., Alonso M.J. (2001). Chitosan nanoparticles: A new vehicle for the improvement of the delivery of drugs to the ocular surface. Application to cyclosporin A. Int. J. Pharm..

[112] Motwani S.K., Chopra S., Talegaonkar S., Kohli K., Ahmad F.J., Khar R.K. (2008). Chitosan-sodium alginate nanoparticles as submicroscopic reservoirs for ocular delivery: Formulation, optimisation and in vitro characterisation. Eur. J. Pharm. Biopharm..

[113] Mahmoud A.A., El-Feky G.S., Kamel R., Awad G.E.A. (2011). Chitosan/sulfobutylether-β-cyclodextrin nanoparticles as a potential approach for ocular drug delivery. Int. J. Pharm..

[114] Sadeghi A.M.M., Dorkoosh F.A., Avadi M.R., Weinhold M., Bayat A., Delie F., Gurny R., Larijani B., Rafiee-Tehrani M., Junginger H.E. (2008). Permeation enhancer effect of chitosan and chitosan derivatives: Comparison of formulations as soluble polymers and nanoparticulate systems on insulin absorption in Caco-2 cells. Eur. J. Pharm. Biopharm..

[115] Mei D., Mao S., Sun W., Wang Y., Kissel T. (2008). Effect of chitosan structure properties and molecular weight on the intranasal absorption of tetramethylpyrazine phosphate in rats. Eur. J. Pharm. Biopharm..

[116] De Campos A.M., Diebold Y., Carvalho E.L.S., Sánchez A., Alonso M.J. (2004). Chitosan nanoparticles as new ocular drug delivery systems: In vitro stability, in vivo fate, and cellular toxicity. Pharm. Res..

[117] Boddu S.H.S. (2012). Polymeric nanoparticles for ophthalmic drug delivery: An update on research and patenting activity. Recent Pat. Nanomed..

[118] Das S., Suresh P.K. (2010). Drug delivery to eye: Special reference to nanoparticle. Int. J. Drug Deliv..

[119] Giarmoukakis A., Labiris G., Sideroudi H., Tsimali Z., Koutsospyrou N., Avgoustakis K., Kozobolis V. (2013). Biodegradable nanoparticles for controlled subconjunctival delivery of latanoprost acid: In vitro and in vivo evaluation. Preliminary results. Exp. Eye Res..

[120] Sosnik A., Das Neves J., Sarmento B. (2014). Mucoadhesive polymers in the design of nano-drug delivery systems for administration by non-parenteral routes: A review. Prog. Polym. Sci..

[121] Almeida H., Amaral M.H., Lobão P., Silva A.C., Lobo J.M.S. (2014). Applications of polymeric and lipid nanoparticles in ophthalmic pharmaceutical formulations: Present and future considerations. J. Pharm. Pharm. Sci..

[122] Lai S.K., O’Hanlon D.E., Harrold S., Man S.T., Wang Y.Y., Cone R., Hanes J. (2007). Rapid transport of large polymeric nanoparticles in fresh undiluted human mucus. Proc. Natl. Acad. Sci. USA.

[123] Lai S.K., Wang Y.Y., Hida K., Cone R., Hanes J. (2010). Nanoparticles reveal that human cervicovaginal mucus is riddled with pores larger than viruses. Proc. Natl. Acad. Sci. USA.

[124] Ding D., Kundukad B., Somasundar A., Vijayan S., Khan S.A., Doyle P.S. (2018). Design of mucoadhesive PLGA microparticles for ocular drug delivery. ACS Appl. Bio. Mater..

[125] Felt O., Furrer P., Mayer J.M., Plazonnet B., Buri P., Gurny R. (1999). Topical use of chitosan in ophthalmology: Tolerance assessment and evaluation of precorneal retention. Int. J. Pharm..

[126] Taghe S., Mirzaeei S. (2019). Preparation and characterization of novel, mucoadhesive of ofloxacin nanoparticles for ocular drug delivery. Braz. J. Pharm. Sci..

[127] Quiñones J.P., Peniche H., Peniche C. (2018). Chitosan based self-assembled nanoparticles in drug delivery. Polymers.

[128] Bodmeier R., Chen H., Paeratakul O. (1989). A novel approach to the oral delivery of micro- or nanoparticles. Pharm. Res..

[129] Silva N.C., Silva S., Sarmento B., Pintado M. (2015). Chitosan nanoparticles for daptomycin delivery in ocular treatment of bacterial endophthalmitis. Drug Deliv..

[130] Koukaras E.N., Papadimitriou S.A., Bikiaris D.N., Froudakis G.E. (2012). Insight on the formation of chitosan nanoparticles through ionotropic gelation with tripolyphosphate. Mol. Pharm..

[131] Alqahtani F.Y., Aleanizy F.S., Tahir E.E., Alquadeib B.T., Alsarra I.A., Alanazi J.S., Abdelhady H.G. (2019). Preparation, characterization, and antibacterial activity of diclofenac-loaded chitosan nanoparticles. Saudi Pharm. J..

[132] Badiee P., Varshochian R., Rafiee-Tehrani M., Abedin Dorkoosh F., Khoshayand M.R., Dinarvand R. (2018). Ocular implant containing bevacizumab-loaded chitosan nanoparticles intended for choroidal neovascularization treatment. J. Biomed. Mater. Res. Part A.

[133] Abul Kalam M., Khan A.A., Khan S., Almalik A., Alshamsan A. (2016). Optimizing indomethacin-loaded chitosan nanoparticle size, encapsulation, and release using Box-Behnken experimental design. Int. J. Biol. Macromol..

[134] Manchanda S., Sahoo P.K. (2018). Fabrication and characterization of mucoadhesive topical nanoformulations of dorzolamide HCl for ocular hypertension. J. Pharm. Investig..

[135] Imam S.S., Bukhari S.N.A., Ahmad J., Ali A. (2018). Formulation and optimization of levofloxacin loaded chitosan nanoparticle for ocular delivery: In-vitro characterization, ocular tolerance and antibacterial activity. Int. J. Biol. Macromol..

[136] Manchanda S., Sahoo P.K. (2017). Topical delivery of acetazolamide by encapsulating in mucoadhesive nanoparticles. Asian J. Pharm. Sci..

[137] Lazaridou M., Christodoulou E., Nerantzaki M., Kostoglou M., Lambropoulou D.A., Katsarou A., Pantopoulos K., Bikiaris D.N. (2020). Formulation and in-vitro characterization of chitosan-nanoparticles loaded with the iron chelator deferoxamine mesylate (DFO). Pharmaceutics.

[138] Morsi N., Ghorab D., Refai H., Teba H. (2017). Nanodispersion-loaded mucoadhesive polymeric inserts for prolonged treatment of post-operative ocular inflammation. J. Microencapsul..

[139] Sabbagh H.A.K., Abudayeh Z., Abudoleh S.M., Alkrad J.A., Hussein M.Z., Hussein-Al-Ali S.H. (2019). Application of multiple regression analysis in optimization of metronidazole-chitosan nanoparticles. J. Polym. Res..

[140] Abdelrahman A.A., Salem H.F., Khallaf R.A., Ali A.M.A. (2015). Modeling, optimization, and in vitro corneal permeation of chitosan-lomefloxacin hcl nanosuspension intended for ophthalmic delivery. J. Pharm. Innov..

[141] Koukaras E.N., Papadimitriou S.A., Bikiaris D.N., Froudakis G.E. (2014). Properties and energetics for design and characterization of chitosan nanoparticles used for drug encapsulation. RSC Adv..

[142] Fathalla Z.M.A., Khaled K.A., Hussein A.K., Alany R.G., Vangala A. (2016). Formulation and corneal permeation of ketorolac tromethamine-loaded chitosan nanoparticles. Drug Dev. Ind. Pharm..

[143] Kalam M.A. (2016). Development of chitosan nanoparticles coated with hyaluronic acid for topical ocular delivery of dexamethasone. Int. J. Biol. Macromol..

[144] Barwal I., Kumar R., Dada T., Yadav S.C. (2019). Effect of ultra-small chitosan nanoparticles doped with brimonidine on the ultra-structure of the trabecular meshwork of glaucoma patients. Microsc. Microanal..

[145] Sharma S., Sharma A., Singh Sara U., Singh S. (2018). Chitosan loaded ketorolac tromethamine nanoparticles for improved ocular delivery in eye inflammation. Indian J. Pharm. Educ. Res..

[146] Chiesa E., Greco A., Riva F., Tosca E.M., Dorati R., Pisani S., Modena T., Conti B., Genta I. (2019). Staggered herringbone microfluid device for the manufacturing of chitosan/TPP nanoparticles: Systematic optimization and preliminary biological evaluation. Int. J. Mol. Sci..

[147] Han Y., Xu X., Wang Y., Liu S., Zhao X., Chen H., Lin Q. (2018). Drug eluting intraocular lens surface modification for PCO prevention. Colloids Interface Sci. Commun..

[148] Han Y., Tang J., Xia J., Wang R., Qin C., Liu S., Zhao X., Chen H., Lin Q. (2019). Anti-adhesive and antiproliferative synergistic surface modification of intraocular lens for reduced posterior capsular opacification. Int. J. Nanomed..

[149] Chhonker Y.S., Prasad Y.D., Chandasana H., Vishvkarma A., Mitra K., Shukla P.K., Bhatta R.S. (2015). Amphotericin-B entrapped lecithin/chitosan nanoparticles for prolonged ocular application. Int. J. Biol. Macromol..

[150] Sunkireddy P., Kanwar R.K., Ram J., Kanwar J.R. (2016). Ultra-small algal chitosan ocular nanoparticles with iron-binding milk protein prevents the toxic effects of carbendazim pesticide. Nanomedicine.

[151] Behl G., Iqbal J., O’Reilly N.J., McLoughlin P., Fitzhenry L. (2016). Synthesis and characterization of poly(2-hydroxyethylmethacrylate) contact lenses containing chitosan nanoparticles as an ocular delivery system for dexamethasone sodium phosphate. Pharm. Res..

[152] López-López M., Fernández-Delgado A., Moyá M.L., Blanco-Arévalo D., Carrera C., De la Haba R.R., Ventosa A., Bernal E., López-Cornejo P. (2019). Optimized preparation of levofloxacin loaded polymeric nanoparticles. Pharmaceutics.

[153] Kong X., Xu W., Zhang C., Kong W. (2018). Chitosan temperature-sensitive gel loaded with drug microspheres has excellent effectiveness, biocompatibility and safety as an ophthalmic drug delivery system. Exp. Ther. Med..

[154] Marei N., Elwahy A.H.M., Salah T.A., El Sherif Y., El-Samie E.A. (2019). Enhanced antibacterial activity of Egyptian local insects’ chitosan-based nanoparticles loaded with ciprofloxacin-HCl. Int. J. Biol. Macromol..

[155] Liu D., Lian Y., Fang Q., Liu L., Zhang J., Li J. (2018). Hyaluronic-acid-modified lipid-polymer hybrid nanoparticles as an efficient ocular delivery platform for moxifloxacin hydrochloride. Int. J. Biol. Macromol..

[156] Kapanigowda U.G., Nagaraja S.H., Ramaiah B., Boggarapu P.R. (2015). Improved intraocular bioavailability of ganciclovir by mucoadhesive polymer based ocular microspheres: Development and simulation process in Wistar rats. DARU J. Pharm. Sci..

[157] Al-Kinani A.A., Naughton D.P., Calabrese G., Vangala A., Smith J.R., Pierscionek B.K., Alany R.G. (2015). Analysis of 2-oxothiazolidine-4-carboxylic acid by hydrophilic interaction liquid chromatography: Application for ocular delivery using chitosan nanoparticles. Anal. Bioanal. Chem..

[158] Elsaid N., Jackson T.L., Elsaid Z., Alqathama A., Somavarapu S. (2016). PLGA microparticles entrapping chitosan-based nanoparticles for the ocular delivery of ranibizumab. Mol. Pharm..

[159] Ibrahim M.M., Abd-Elgawad A.E.H., Soliman O.A.E., Jablonski M.M. (2016). Stability and Ocular pharmacokinetics of celecoxib-loaded nanoparticles topical ophthalmic formulations. J. Pharm. Sci..

[160] Kelly S.J., Halasz K., Smalling R., Sutariya V. (2019). Nanodelivery of doxorubicin for age-related macular degeneration. Drug Dev. Ind. Pharm..

[161] Kalam M.A. (2016). The potential application of hyaluronic acid coated chitosan nanoparticles in ocular delivery of dexamethasone. Int. J. Biol. Macromol..

[162] Khan S., Warade S., Singhavi D.J. (2018). Improvement in ocular bioavailability and prolonged delivery of tobramycin sulfate following topical ophthalmic administration of drug-loaded mucoadhesive microparticles incorporated in thermosensitive in situ gel. J. Ocul. Pharmacol. Ther..

[163] Da Silva S.B., Ferreira D., Pintado M., Sarmento B. (2016). Chitosan-based nanoparticles for rosmarinic acid ocular delivery-In vitro tests. Int. J. Biol. Macromol..

[164] Jamil B., Abbasi R., Abbasi S., Imran M., Khan S.U., Ihsan A., Javed S., Bokhari H. (2016). Encapsulation of cardamom essential oil in chitosan nano-composites: In-vitro efficacy on antibiotic-resistant bacterial pathogens and cytotoxicity studies. Front. Microbiol..

[165] Elkadery A.A.S., Elsherif E.A., Ezz Eldin H.M., Fahmy I.A.F., Mohammad O.S. (2019). Efficient therapeutic effect of Nigella sativa aqueous extract and chitosan nanoparticles against experimentally induced Acanthamoeba keratitis. Parasitol. Res..

[166] Hafner A., Lovrić J., Romić M.D., Juretić M., Pepić I., Cetina-Čižmek B., Filipović-Grčić J. (2015). Evaluation of cationic nanosystems with melatonin using an eye-related bioavailability prediction model. Eur. J. Pharm. Sci..

[167] Dubey V., Mohan P., Dangi J.S., Kesavan K. (2019). Brinzolamide loaded chitosan-pectin mucoadhesive nanocapsules for management of glaucoma: Formulation, characterization and pharmacodynamic study. Int. J. Biol. Macromol..

[168] Muhtadi W.K., Novitasari L., Martien R., Danarti R. (2019). Factorial design as the method in the optimization of timolol maleate-loaded nanoparticle prepared by ionic gelation technique. Int. J. Appl. Pharm..

[169] Ilka R., Mohseni M., Kianirad M., Naseripour M., Ashtari K., Mehravi B. (2018). Nanogel-based natural polymers as smart carriers for the controlled delivery of Timolol Maleate through the cornea for glaucoma. Int. J. Biol. Macromol..

[170] Ahdyani R., Novitasari L., Martien R., Danarti R. (2019). Formulation and characterization of timolol maleate-loaded nanoparticles gel by ionic gelation method using chitosan and sodium alginate. Int. J. Appl. Pharm..

[171] Gong Y., Yin J.Y., Tong B.D., Zeng J.X., Xiong W. (2017). Low density lipoprotein—Rosiglitazone—chitosan-calcium alginate/nanoparticles inhibition of human tenon’s fibroblasts activation and proliferation. Oncotarget.

[172] Ahuja M., Bhatt D.C. (2015). Carboxymethyl gum katira: Synthesis, characterization and evaluation for nanoparticulate drug delivery. RSC Adv..

[173] Mittal N., Kaur G. (2019). Investigations on polymeric nanoparticles for ocular delivery. Adv. Polym. Technol..

[174] Chaiyasan W., Srinivas S.P., Tiyaboonchai W. (2015). Crosslinked chitosan-dextran sulfate nanoparticle for improved topical ocular drug delivery. Mol. Vis..

[175] Manchanda S., Sahoo P.K., Majumdar D.K. (2016). Mucoadhesive chitosan-dextran sulfate nanoparticles of acetazolamide for ocular hypertension. Nanotechnol. Rev..

[176] Chavan C., Bala P., Pal K., Kale S.N. (2017). Cross-linked chitosan-dextran sulphate vehicle system for controlled release of ciprofloxaxin drug: An ophthalmic application. OpenNano.

[177] Silva B., Marto J., Braz B.S., Delgado E., Almeida A.J., Gonçalves L. (2020). New nanoparticles for topical ocular delivery of erythropoietin. Int. J. Pharm..

[178] Fabiano A., Bizzarri R., Zambito Y. (2017). Thermosensitive hydrogel based on chitosan and its derivatives containing medicated nanoparticles for transcorneal administration of 5-fluorouracil. Int. J. Nanomed..

[179] Zhang P., Liu X., Hu W., Bai Y., Zhang L. (2016). Preparation and evaluation of naringenin-loaded sulfobutylether-β-cyclodextrin/chitosan nanoparticles for ocular drug delivery. Carbohydr. Polym..

[180] Alqurshi A., Hanafy A.F., Abdalla A.M., Guda T.K., Gabr K.E., Royall P.G. (2019). Ocular anti-inflammatory activity of prednisolone acetate loaded chitosan-deoxycholate self-assembled nanoparticles. Int. J. Nanomed..

[181] Russo E., Villa C. (2019). Poloxamer hydrogels for biomedical applications. Pharmaceutics.

[182] Ahuja M., Verma P., Bhatia M. (2015). Preparation and evaluation of chitosan–itraconazole co-precipitated nanosuspension for ocular delivery. J. Exp. Nanosci..

[183] Natesan S., Pandian S., Ponnusamy C., Palanichamy R., Muthusamy S., Kandasamy R. (2017). Co-encapsulated resveratrol and quercetin in chitosan and peg modified chitosan nanoparticles: For efficient intra ocular pressure reduction. Int. J. Biol. Macromol..

[184] Åhlén M., Tummala G.K., Mihranyan A. (2018). Nanoparticle-loaded hydrogels as a pathway for enzyme-triggered drug release in ophthalmic applications. Int. J. Pharm..

[185] Tellios V., Liu H., Tellios N., Li X., Hutnik C.M.L. (2018). Administration of nitric oxide through a novel copper- chitosan delivery system in human corneal and limbal epithelial cell injury. Investig. Ophthalmol. Vis. Sci..

[186] Dhawan S., Singla A.K., Sinha V.R. (2004). Evaluation of mucoadhesive properties of chitosan microspheres prepared by different methods. AAPS PharmSciTech.

[187] Fefelova N.A., Nurkeeva Z.S., Mun G.A., Khutoryanskiy V.V. (2007). Mucoadhesive interactions of amphiphilic cationic copolymers based on [2-(methacryloyloxy)ethyl]trimethylammonium chloride. Int. J. Pharm..

[188] Hejjaji E.M.A., Smith A.M., Morris G.A. (2018). Evaluation of the mucoadhesive properties of chitosan nanoparticles prepared using different chitosan to tripolyphosphate (CS:TPP) ratios. Int. J. Biol. Macromol..

[189] Asasutjarit R., Theerachayanan T., Kewsuwan P., Veeranodha S., Fuongfuchat A., Ritthidej G.C. (2015). Development and evaluation of diclofenac sodium loaded-N-trimethyl chitosan nanoparticles for ophthalmic use. AAPS PharmSciTech.

[190] Shinde U.A., Joshi P.N., Jain D.D., Singh K. (2019). Preparation and evaluation of N-trimethyl chitosan nanoparticles of flurbiprofen for ocular delivery. Curr. Eye Res..

[191] Siafaka P.I., Titopoulou A., Koukaras E.N., Kostoglou M., Koutris E., Karavas E., Bikiaris D.N. (2015). Chitosan derivatives as effective nanocarriers for ocular release of timolol drug. Int. J. Pharm..

[192] Koutroumanis K.P., Avgoustakis K., Bikiaris D. (2010). Synthesis of cross-linked N-(2-carboxybenzyl)chitosan pH sensitive polyelectrolyte and its use for drug controlled delivery. Carbohydr. Polym..

[193] Ambhore N.P., Dandagi P.M., Gadad A.P. (2016). Formulation and comparative evaluation of HPMC and water soluble chitosan-based sparfloxacin nanosuspension for ophthalmic delivery. Drug Deliv. Transl. Res..

[194] Jaiswal S., Dutta P.K., Kumar S., Koh J., Pandey S. (2019). Methyl methacrylate modified chitosan: Synthesis, characterization and application in drug and gene delivery. Carbohydr. Polym..

[195] Rajawat G.S., Shinde U.A., Nair H.A. (2016). Chitosan-N-acetyl cysteine microspheres for ocular delivery of acyclovir: Synthesis and in vitro/in vivo evaluation. J. Drug Deliv. Sci. Technol..

[196] Mauro N., Di Prima G., Varvarà P., Licciardi M., Cavallaro G., Giammona G. (2019). Core-Shell Arginine-Containing Chitosan Microparticles for Enhanced Transcorneal Permeation of Drugs. J. Pharm. Sci..

[197] Savin C.L., Popa M., Delaite C., Costuleanu M., Costin D., Peptu C.A. (2019). Chitosan grafted-poly(ethylene glycol) methacrylate nanoparticles as carrier for controlled release of bevacizumab. Mater. Sci. Eng. C.

[198] Yu F., Zheng M., Zhang A.Y., Han Z. (2019). A cerium oxide loaded glycol chitosan nano-system for the treatment of dry eye disease. J. Control. Release.

[199] Yu A., Shi H., Liu H., Bao Z., Dai M., Lin D.D., Lin D.D., Xu X., Li X., Wang Y. (2020). Mucoadhesive dexamethasone-glycol chitosan nanoparticles for ophthalmic drug delivery. Int. J. Pharm..

[200] Sun X., Yu D., Ying Z., Pan C., Wang N., Huang F., Ling J., Ouyang X.K. (2019). Fabrication of ion-crosslinking aminochitosan nanoparticles for encapsulation and slow release of curcumin. Pharmaceutics.

[201] Zhao R., Li J., Wang J., Yin Z., Zhu Y., Liu W. (2017). Development of Timolol-Loaded Galactosylated Chitosan Nanoparticles and Evaluation of Their Potential for Ocular Drug Delivery. AAPS PharmSciTech.

[202] Piras A.M., Zambito Y., Burgalassi S., Monti D., Tampucci S., Terreni E., Fabiano A., Balzano F., Uccello-Barretta G., Chetoni P. (2018). A water-soluble, mucoadhesive quaternary ammonium chitosan-methyl-β-cyclodextrin conjugate forming inclusion complexes with dexamethasone. J. Mater. Sci. Mater. Med..

[203] Fabiano A., Piras A.M., Guazzelli L., Storti B., Bizzarri R., Zambito Y. (2019). Impact of different mucoadhesive polymeric nanoparticles loaded in thermosensitive hydrogels on transcorneal administration of 5-fluorouracil. Pharmaceutics.

[204] Addo R.T., Yeboah K.G., Siwale R.C., Siddig A., Jones A., Ubale R.V., Akande J., Nettey H., Patel N.J., Addo E. (2015). Formulation and characterization of atropine sulfate in albumin-chitosan microparticles for in vivo ocular drug delivery. J. Pharm. Sci..

[205] Zhou J., Chen Y., Luo M., Deng F., Lin S., Wu W., Li G., Nan K. (2019). Dual cross-linked chitosan microspheres formulated with spray-drying technique for the sustained release of levofloxacin. Drug Dev. Ind. Pharm..

[206] Başaran E., Şenel B., Kirimlioğlu G.Y., Güven U.M., Yazan Y. (2015). Ornidazole incorporated chitosan nanoparticles for ocular application. Lat. Am. J. Pharm..

[207] Costa C., Louvisse de Abreu L., Dos Santos E., Franca Presgrave O., Rocha Pierucci A.P., Rodrigues C., De Sousa V., Nicoli S., Junior E., Cabral L. (2015). Preparation and evaluation of chitosan submicroparticles containing pilocarpine for glaucoma therapy. Curr. Drug Deliv..

[208] Husseini G.A., Pitt W.G. (2008). Micelles and nanoparticles for ultrasonic drug and gene delivery. Adv. Drug Deliv. Rev..

[209] Gaucher G., Marchessault R.H., Leroux J.C. (2010). Polyester-based micelles and nanoparticles for the parenteral delivery of taxanes. J. Control. Release.

[210] Majidinia M., Mirza-Aghazadeh-Attari M., Rahimi M., Mihanfar A., Karimian A., Safa A., Yousefi B. (2020). Overcoming multidrug resistance in cancer: Recent progress in nanotechnology and new horizons. IUBMB Life.

[211] Fang X., Cao J., Shen A. (2020). Advances in anti-breast cancer drugs and the application of nano-drug delivery systems in breast cancer therapy. J. Drug Deliv. Sci. Technol..

[212] Bonferoni M.C., Sandri G., Dellera E., Rossi S., Ferrari F., Zambito Y., Caramella C. (2016). Palmitoyl glycol chitosan micelles for corneal delivery of cyclosporine. J. Biomed. Nanotechnol..

[213] Xu X., Sun L., Zhou L., Cheng Y., Cao F. (2020). Functional chitosan oligosaccharide nanomicelles for topical ocular drug delivery of dexamethasone. Carbohydr. Polym..

[214] Shi S., Zhang Z., Luo Z., Yu J., Liang R., Li X., Chen H. (2015). Chitosan grafted methoxy poly(ethylene glycol)-poly(ε-caprolactone) nanosuspension for ocular delivery of hydrophobic diclofenac. Sci. Rep..

[215] Carvalho S.G., Araujo V.H.S., Dos Santos A.M., Duarte J.L., Silvestre A.L.P., Fonseca-Santos B., Villanova J.C.O., Gremião M.P.D., Chorilli M. (2020). Advances and challenges in nanocarriers and nanomedicines for veterinary application. Int. J. Pharm..

[216] Ibrahim Y.H.E.Y., Regdon G., Hamedelniel E.I., Sovány T. (2019). Review of recently used techniques and materials to improve the efficiency of orally administered proteins/peptides. DARU J. Pharm. Sci..

[217] Zhao F., Lu J., Jin X., Wang Z., Sun Y., Gao D., Li X., Liu R. (2018). Comparison of response surface methodology and artificial neural network to optimize novel ophthalmic flexible nano-liposomes: Characterization, evaluation, in vivo pharmacokinetics and molecular dynamics simulation. Colloids Surf. B.

[218] Abd-Elsalam W.H., ElKasabgy N.A. (2019). Mucoadhesive olaminosomes: A novel prolonged release nanocarrier of agomelatine for the treatment of ocular hypertension. Int. J. Pharm..

[219] Liu R., Wang S., Sun L., Fang S., Wang J., Huang X., You Z., He X., Liu C. (2016). A novel cationic nanostructured lipid carrier for improvement of ocular bioavailability: Design, optimization, in vitro and in vivo evaluation. J. Drug Deliv. Sci. Technol..

[220] Liu R., Wang S., Fang S., Wang J., Chen J., Huang X., He X., Liu C. (2016). Liquid Crystalline Nanoparticles as an Ophthalmic Delivery System for Tetrandrine: Development, Characterization, and In Vitro and In Vivo Evaluation. Nanoscale Res. Lett..

[221] Cui C.L., Gan L., Lan X.Y., Li J., Zhang C.F., Li F., Wang C.Z., Yuan C.S. (2019). Development of sustainable carrier in thermosensitive hydrogel based on chitosan/alginate nanoparticles for in situ delivery system. Polym. Compos..

[222] Xie J., Wang C., Ning Q., Gao Q., Gao C., Gou Z., Ye J. (2017). A new strategy to sustained release of ocular drugs by one-step drug-loaded microcapsule manufacturing in hydrogel punctal plugs. Graefes Arch. Clin. Exp. Ophthalmol..

[223] Kirchhof S., Goepferich A.M., Brandl F.P. (2015). Hydrogels in ophthalmic applications. Eur. J. Pharm. Biopharm..

[224] Shatz W., Aaronson J., Yohe S., Kelley R.F., Kalia Y.N. (2019). Strategies for modifying drug residence time and ocular bioavailability to decrease treatment frequency for back of the eye diseases. Expert Opin. Drug Deliv..

[225] Cook M.T., Schmidt S.A., Lee E., Samprasit W., Opanasopit P., Khutoryanskiy V.V. (2015). Synthesis of mucoadhesive thiol-bearing microgels from 2-(acetylthio)ethylacrylate and 2-hydroxyethylmethacrylate: Novel drug delivery systems for chemotherapeutic agents to the bladder. J. Mater. Chem. B.

[226] Hanafy N.A.N., Leporatti S., El-Kemary M.A. (2019). Mucoadhesive hydrogel nanoparticles as smart biomedical drug delivery system. Appl. Sci..

[227] Madsen F., Eberth K., Smart J.D. (1998). A rheological assessment of the nature of interactions between mucoadhesive polymers and a homogenised mucus gel. Biomaterials.

[228] Irimia T., Dinu-Pîrvu C.E., Ghica M.V., Lupuleasa D., Muntean D.L., Udeanu D.I., Popa L. (2018). Chitosan-based in situ gels for ocular delivery of therapeutics: A state-of-the-art review. Mar. Drugs.

[229] Chowhan A., Giri T.K. (2020). Polysaccharide as renewable responsive biopolymer for in situ gel in the delivery of drug through ocular route. Int. J. Biol. Macromol..

[230] Dubashynskaya N., Poshina D., Raik S., Urtti A., Skorik Y.A. (2020). Polysaccharides in ocular drug delivery. Pharmaceutics.

[231] Fouda N.H., Abdelrehim R.T., Hegazy D.A., Habib B.A. (2018). Sustained ocular delivery of Dorzolamide-HCl via proniosomal gel formulation: In-vitro characterization, statistical optimization, and in-vivo pharmacodynamic evaluation in rabbits. Drug Deliv..

[232] Abdel-Rashid R.S., Helal D.A., Omar M.M., El Sisi A.M. (2019). Nanogel loaded with surfactant based nanovesicles for enhanced ocular delivery of acetazolamide. Int. J. Nanomed..

[233] Mohammed S., Chouhan G., Anuforom O., Cooke M., Walsh A., Morgan-Warren P., Jenkins M., De Cogan F. (2017). Thermosensitive hydrogel as an in situ gelling antimicrobial ocular dressing. Mater. Sci. Eng. C.

[234] Chenite A., Buschmann M., Wang D., Chaput C., Kandani N. (2001). Rheological characterisation of thermogelling chitosan/glycerol-phosphate solutions. Carbohydr. Polym..

[235] Irimia T., Muşat G.C., Prisada R.M., Ghica M.V., Dinu-Pîrvu C.E., Anuţa V., Velescu B.Ş., Popa L. (2019). Contributions on formulation and preliminary evaluation of ocular colloidal systems of chitosan and poloxamer 407 with bupivacaine hydrochloride. Farmacia.

[236] Krtalić I., Radošević S., Hafner A., Grassi M., Nenadić M., Cetina-Čižmek B., Filipović-Grčić J., Pepić I., Lovrić J. (2018). D-Optimal Design in the Development of Rheologically Improved In Situ Forming Ophthalmic Gel. J. Pharm. Sci..

[237] Deepthi S., Jose J. (2019). Novel hydrogel-based ocular drug delivery system for the treatment of conjunctivitis. Int. Ophthalmol..

[238] Almeida H., Lobão P., Frigerio C., Fonseca J., Silva R., Quaresma P., Lobo J.M.S., Amaral M.H. (2016). Development of mucoadhesive and thermosensitive eyedrops to improve the ophthalmic bioavailability of ibuprofen. J. Drug Deliv. Sci. Technol..

[239] Ahmed T.A., Aljaeid B.M. (2017). A potential in situ gel formulation loaded with novel fabricated poly(Lactide-co-glycolide) nanoparticles for enhancing and sustaining the ophthalmic delivery of ketoconazole. Int. J. Nanomed..

[240] Paulsamy M., Ponnusamy C., Palanisami M., Nackeeran G., Paramasivam S.S., Sugumaran A., Kandasamy R., Natesan S., Palanichamy R. (2018). Nepafenac loaded silica nanoparticles dispersed in-situ gel systems: Development and characterization. Int. J. Biol. Macromol..

[241] Cruz-Cazarim E.L.C., Cazarim M.S., Ogunjimi A.T., Petrilli R., Rocha E.M., Lopez R.F. (2019). V Prospective insulin-based ophthalmic delivery systems for the treatment of dry eye syndrome and corneal injuries. Eur. J. Pharm. Biopharm..

[242] Arvind, Lamba H.S., Ali A. (2017). In-Situ Gel System Based on Temperature and pH Activation for Sustained Ocular Delivery. Indo Am. J. P Sci..

[243] Gade S.K., Shivshetty N., Sharma N., Bhatnagar S., Garg P., Venuganti V.V.K. (2018). Effect of Mucoadhesive Polymeric Formulation on Corneal Permeation of Fluoroquinolones. J. Ocul. Pharmacol. Ther..

[244] Rose J.B., Pacelli S., El Haj A.J., Dua H.S., Hopkinson A., White L.J., Rose F.R.A.J. (2014). Gelatin-based materials in ocular tissue engineering. Materials.

[245] Tsai C.-Y., Woung L.-C., Yen J.-C., Tseng P.-C., Chiou S.-H., Sung Y.-J., Liu K.-T., Cheng Y.-H. (2016). Thermosensitive chitosan-based hydrogels for sustained release of ferulic acid on corneal wound healing. Carbohydr. Polym..

[246] Cheng Y.H., Tsai T.H., Jhan Y.Y., Chiu A.W.H., Tsai K.L., Chien C.S., Chiou S.H., Liu C.J.L. (2016). Thermosensitive chitosan-based hydrogel as a topical ocular drug delivery system of latanoprost for glaucoma treatment. Carbohydr. Polym..

[247] Cheng Y.H., Ko Y.C., Chang Y.F., Huang S.H., Liu C.J. (2019). Thermosensitive chitosan-gelatin-based hydrogel containing curcumin-loaded nanoparticles and latanoprost as a dual-drug delivery system for glaucoma treatment. Exp. Eye Res..

[248] Cheng Y.H., Chang Y.F., Ko Y.C., Liu C.J. (2020). Sustained release of levofloxacin from thermosensitive chitosan-based hydrogel for the treatment of postoperative endophthalmitis. J. Biomed. Mater. Res. Part B Appl. Biomater..

[249] Nagai N., Saijo S., Song Y., Kaji H., Abe T. (2019). A drug refillable device for transscleral sustained drug delivery to the retina. Eur. J. Pharm. Biopharm..

[250] Song Y., Nagai N., Saijo S., Kaji H., Nishizawa M., Abe T. (2018). In situ formation of injectable chitosan-gelatin hydrogels through double crosslinking for sustained intraocular drug delivery. Mater. Sci. Eng. C.

[251] El-Feky G.S., Zayed G.M., Elshaier Y.A.M.M., Alsharif F.M. (2018). Chitosan-gelatin hydrogel crosslinked with oxidized sucrose for the ocular delivery of timolol maleate. J. Pharm. Sci..

[252] Chiesa E., Genta I., Dorati R., Modena T., Conti B. (2019). Poly(gamma-glutamic acid) based thermosetting hydrogels for injection: Rheology and functional parameters evaluation. React. Funct. Polym..

[253] Liu J., Yang S., Li X., Yan Q., Reaney M.J.T., Jiang Z. (2019). Alginate oligosaccharides: Production, biological activities, and potential applications. Compr. Rev. Food Sci. Food Saf..

[254] Hernández-González A.C., Téllez-Jurado L., Rodríguez-Lorenzo L.M. (2020). Alginate hydrogels for bone tissue engineering, from injectables to bioprinting: A review. Carbohydr. Polym..

[255] Gupta H., Aqil M., Khar R., Ali A., Bhatnagar A., Mittal G. (2015). An alternative in situ gel-formulation of levofloxacin eye drops for prolong ocular retention. J. Pharm. Bioallied Sci..

[256] Giavasis I., Harvey L.M., McNeil B. (2000). Gellan gum. Crit. Rev. Biotechnol..

[257] Gupta H., Malik A., Khar R.K., Ali A., Bhatnagar A., Mittal G. (2015). Physiologically active hydrogel (in situ gel) of sparfloxacin and its evaluation for ocular retention using gamma scintigraphy. J. Pharm. Bioallied Sci..

[258] Ameeduzzafar, Imam S.S., Bukhari S.N.A., Ali A. (2018). Preparation and evaluation of novel chitosan: Gelrite ocular system containing besifloxacin for topical treatment of bacterial conjunctivitis: Scintigraphy, ocular irritation and retention assessment. Artif. Cells Nanomed. Biotechnol..

[259] Jiang F., Tang Z., Zhang Y., Ju Y., Gao H., Sun N., Liu F., Gu P., Zhang W. (2019). Enhanced proliferation and differentiation of retinal progenitor cells through a self-healing injectable hydrogel. Biomater. Sci..

[260] Muţ A.M., Vlaia L., Coneac G., Olariu I., Vlaia V., Popoiu C., Hîrjău M., Lupuliasa D. (2018). Novel topical chitosan/hydroxypropylmethylcellulose—Based hydrogels containing fluconazole and sucrose esters. Formulation, physicochemical characterization, in vitro drug release and permeation. Farmacia.

[261] Yang C., Gao L., Liu X., Yang T., Yin G., Chen J., Guo H., Yu B., Cong H. (2019). Injectable Schiff base polysaccharide hydrogels for intraocular drug loading and release. J. Biomed. Mater. Res. Part A.

[262] Xu W., Liu K., Li T., Zhang W., Dong Y., Lv J., Wang W., Sun J., Li M., Wang M. (2019). An in situ hydrogel based on carboxymethyl chitosan and sodium alginate dialdehyde for corneal wound healing after alkali burn. J. Biomed. Mater. Res. Part A.

[263] Lei L., Li X., Xiong T., Yu J., Yu X., Song Z., Li X. (2018). Covalently cross-linked chitosan hydrogel sheet for topical ophthalmic delivery of levofloxacin. J. Biomed. Nanotechnol..

[264] Qiao X., Peng X., Qiao J., Jiang Z., Han B. (2017). Evaluation of a photocrosslinkable hydroxyethyl chitosan hydrogel as a potential drug release system for glaucoma surgery. J. Mater. Sci. Mater. Med..

[265] Moreno M., Pow P.Y., Tabitha T.S.T., Nirmal S., Larsson A., Radhakrishnan K., Nirmal J., Quah S.T., Geifman Shochat S., Agrawal R. (2017). Modulating release of ranibizumab and aflibercept from thiolated chitosan-based hydrogels for potential treatment of ocular neovascularization. Expert Opin. Drug Deliv..

[266] Lai J.Y., Luo L.J. (2017). Chitosan-g-poly(N-isopropylacrylamide) copolymers as delivery carriers for intracameral pilocarpine administration. Eur. J. Pharm. Biopharm..

[267] Luo L.J., Huang C.C., Chen H.C., Lai J.Y., Matsusaki M. (2018). Effect of deacetylation degree on controlled pilocarpine release from injectable chitosan-g-poly(N-isopropylacrylamide) carriers. Carbohydr. Polym..

[268] Luo L.J., Nguyen D.D., Lai J.Y. (2020). Benzoic acid derivative-modified chitosan-g-poly(N-isopropylacrylamide): Methoxylation effects and pharmacological treatments of Glaucoma-related neurodegeneration. J. Control. Release.

[269] Tan G., Yu S., Li J., Pan W. (2017). Development and characterization of nanostructured lipid carriers based chitosan thermosensitive hydrogel for delivery of dexamethasone. Int. J. Biol. Macromol..

[270] Cho I.S., Park C.G., Huh B.K., Cho M.O., Khatun Z., Li Z., Kang S.W., Choy Y.B., Huh K.M. (2016). Thermosensitive hexanoyl glycol chitosan-based ocular delivery system for glaucoma therapy. Acta Biomater..

[271] Shi H., Wang Y., Bao Z., Lin D., Liu H., Yu A., Lei L., Li X., Xu X. (2019). Thermosensitive glycol chitosan-based hydrogel as a topical ocular drug delivery system for enhanced ocular bioavailability. Int. J. Pharm..

[272] Oh H.M., Kang E., Li Z., Cho I.S., Kim D.E., Mallick S., Kang S.W., Roh K.H., Huh K.M. (2019). Preparation and characterization of an in situ crosslinkable glycol chitosan thermogel for biomedical applications. J. Ind. Eng. Chem..

[273] Yu S., Zhang X., Tan G., Tian L., Liu D., Liu Y., Yang X., Pan W. (2017). A novel pH-induced thermosensitive hydrogel composed of carboxymethyl chitosan and poloxamer cross-linked by glutaraldehyde for ophthalmic drug delivery. Carbohydr. Polym..

[274] Yu S., Li Q., Li Y., Wang H., Liu D., Yang X., Pan W. (2017). A novel hydrogel with dual temperature and pH responsiveness based on a nanostructured lipid carrier as an ophthalmic delivery system: Enhanced trans-corneal permeability and bioavailability of nepafenac. New J. Chem..

[275] Yu Y., Feng R., Yu S., Li J., Wang Y., Song Y., Yang X., Pan W., Li S. (2018). Nanostructured lipid carrier-based pH and temperature dual-responsive hydrogel composed of carboxymethyl chitosan and poloxamer for drug delivery. Int. J. Biol. Macromol..

[276] Yu Y., Feng R., Li J., Wang Y., Song Y., Tan G., Liu D., Liu W., Yang X., Pan H. (2019). A hybrid genipin-crosslinked dual-sensitive hydrogel/nanostructured lipid carrier ocular drug delivery platform. Asian J. Pharm. Sci..

[277] Lin T., Lu Y., Zhang X., Gong L., Wei C. (2018). Treatment of dry eye by intracanalicular injection of a thermosensitive chitosan-based hydrogel: Evaluation of biosafety and availability. Biomater. Sci..

[278] Franca J.R., Batista L.D., Ribeiro T.G., Fernandes C., Castilho R.O., Faraco A.A.G. (2015). Development and Validation of a High Performance Liquid Chromatographic Method for Determination of Bimatoprost in Chitosan-Based Ocular Inserts. Anal. Lett..

[279] Foureaux G., Franca J.R., Nogueira J.C., Fulgêncio G., De O., Ribeiro T.G., Castilho R.O., Yoshida M.I., Fuscaldi L.L., Fernandes S.O.A. (2015). Ocular inserts for sustained release of the angiotensin-converting enzyme 2 activator, diminazene aceturate, to treat glaucoma in rats. PLoS ONE.

[280] Franca J.R., Foureaux G., Fuscaldi L.L., Ribeiro T.G., Castilho R.O., Yoshida M.I., Cardoso V.N., Fernandes S.O.A., Cronemberger S., Nogueira J.C. (2019). Chitosan/hydroxyethyl cellulose inserts for sustained-release of dorzolamide for glaucoma treatment: In vitro and in vivo evaluation. Int. J. Pharm..

[281] Wang L., Jiang Y.Y., Lin N. (2020). Promise of latanoprost and timolol loaded combinatorial nanosheet for therapeutic applications in glaucoma. J. King Saud Univ. Sci..

[282] Abilova G.K., Kaldybekov D.B., Ozhmukhametova E.K., Saimova A.Z., Kazybayeva D.S., Irmukhametova G.S., Khutoryanskiy V.V. (2019). Chitosan/poly(2-ethyl-2-oxazoline) films for ocular drug delivery: Formulation, miscibility, in vitro and in vivo studies. Eur. Polym. J..

[283] Üstündaʇ-Okur N., Gökçe E.H., Bozbiyik D.I., Eʇrilmez S., Ertan G., Ozer O. (2015). Novel nanostructured lipid carrier-based inserts for controlled ocular drug delivery: Evaluation of corneal bioavailability and treatment efficacy in bacterial keratitis. Expert Opin. Drug Deliv..

[284] Jeencham R., Sutheerawattananonda M., Tiyaboonchai W. (2019). Preparation and characterization of chitosan/regenerated silk fibroin (CS/RSF) films as a biomaterial for contact lenses-based ophthalmic drug delivery system. Int. J. Appl. Pharm..

[285] Huang J.F., Zhong J., Chen G.P., Lin Z.T., Deng Y., Liu Y.L., Cao P.Y., Wang B., Wei Y., Wu T. (2016). A Hydrogel-Based Hybrid Theranostic Contact Lens for Fungal Keratitis. ACS Nano.

[286] Silva D., Pinto L.F.V., Bozukova D., Santos L.F., Serro A.P., Saramago B. (2016). Chitosan/alginate based multilayers to control drug release from ophthalmic lens. Colloids Surf. B.

[287] Hu X., Gong X. (2016). A new route to fabricate biocompatible hydrogels with controlled drug delivery behavior. J. Colloid Interface Sci..

[288] Kazemi Ashtiani M., Zandi M., Shokrollahi P., Ehsani M., Baharvand H. (2020). Chitosan surface modified hydrogel as a therapeutic contact lens. Polym. Adv. Technol..

[289] Mehta P., Al-Kinani A.A., Arshad M.S., Singh N., Van Der Merwe S.M., Chang M.W., Alany R.G., Ahmad Z. (2019). Engineering and Development of Chitosan-Based Nanocoatings for Ocular Contact Lenses. J. Pharm. Sci..

[290] Hoyo J., Ivanova K., Guaus E., Tzanov T. (2019). Multifunctional ZnO NPs-chitosan-gallic acid hybrid nanocoating to overcome contact lenses associated conditions and discomfort. J. Colloid Interface Sci..

[291] Saher O., Ghorab D.M., Mursi N.M. (2016). Levofloxacin hemihydrate ocular semi-sponges for topical treatment of bacterial conjunctivitis: Formulation and in-vitro/in-vivo characterization. J. Drug Deliv. Sci. Technol..

[292] Manna S., Donnell A.M., Kaval N., Al-Rjoub M.F., Augsburger J.J., Banerjee R.K. (2018). Improved design and characterization of PLGA/PLA-coated Chitosan based micro-implants for controlled release of hydrophilic drugs. Int. J. Pharm..

[293] Mirzaeei S., Berenjian K., Khazaei R. (2018). Preparation of the Potential Ocular Inserts by Electrospinning Method to Achieve the Prolong Release Profile of Triamcinolone Acetonide. Adv. Pharm. Bull..

[294] Li J., Cheng T., Tian Q., Cheng Y., Zhao L., Zhang X., Qu Y. (2019). A more efficient ocular delivery system of triamcinolone acetonide as eye drop to the posterior segment of the eye. Drug Deliv..

[295] Cheng T., Li J., Cheng Y., Zhang X., Qu Y. (2019). Triamcinolone acetonide-chitosan coated liposomes efficiently treated retinal edema as eye drops. Exp. Eye Res..

[296] Tan G., Yu S., Pan H., Li J., Liu D., Yuan K., Yang X., Pan W. (2017). Bioadhesive chitosan-loaded liposomes: A more efficient and higher permeable ocular delivery platform for timolol maleate. Int. J. Biol. Macromol..

[297] Khalil M., Hashmi U., Riaz R., Rukh Abbas S. (2020). Chitosan coated liposomes (CCL) containing triamcinolone acetonide for sustained delivery: A potential topical treatment for posterior segment diseases. Int. J. Biol. Macromol..

[298] Chen H., Pan H., Li P., Wang H., Wang X., Pan W., Yuan Y. (2016). The potential use of novel chitosan-coated deformable liposomes in an ocular drug delivery system. Colloids Surf. B.

[299] Sun C.C., Chou S.F., Lai J.Y., Cho C.H., Lee C.H. (2016). Dependence of corneal keratocyte adhesion, spreading, and integrin β1 expression on deacetylated chitosan coating. Mater. Sci. Eng. C.

[300] Eid H.M., Elkomy M.H., El Menshawe S.F., Salem H.F. (2019). Development, optimization, and in vitro/in vivo characterization of enhanced lipid nanoparticles for ocular delivery of ofloxacin: The influence of pegylation and chitosan coating. AAPS PharmSciTech.

[301] Ban J., Zhang Y., Huang X., Deng G., Hou D., Chen Y., Lu Z. (2017). Corneal permeation properties of a charged lipid nanoparticle carrier containing dexamethasone. Int. J. Nanomed..

[302] Gelfuso G.M., Ferreira-Nunes R., Dalmolin L.F., Ana A.C., Dos Santos G.A., De Sá F.A.P., Cunha-Filho M., Alonso A., Neto S.A.M., Anjos J.L.V. (2020). Iontophoresis enhances voriconazole antifungal potency and corneal penetration. Int. J. Pharm..

[303] Seyfoddin A., Sherwin T., Patel D.V., McGhee C.N., Rupenthal I.D., Taylor J.A., Al-Kassas R. (2016). Ex vivo and in vivo evaluation of chitosan coated nanostructured lipid carriers for ocular delivery of acyclovir. Curr. Drug Deliv..

[304] Selvaraj K., Kuppusamy G., Krishnamurthy J., Mahalingam R., Singh S.K., Gulati M. (2019). Repositioning of itraconazole for the management of ocular neovascularization through surface-modified nanostructured lipid carriers. Assay Drug Dev. Technol..

[305] Wang F., Zhang M., Zhang D., Huang Y., Chen L., Jiang S., Shi K., Li R. (2018). Preparation, optimization, and characterization of chitosan-coated solid lipid nanoparticles for ocular drug delivery. J. Biomed. Res..

[306] Jurišić Dukovski B., Juretić M., Bračko D., Randjelović D., Savić S., Crespo Moral M., Diebold Y., Filipović-Grčić J., Pepić I., Lovrić J. (2020). Functional ibuprofen-loaded cationic nanoemulsion: Development and optimization for dry eye disease treatment. Int. J. Pharm..

[307] Zhang J., Liang X., Li X., Guan Z., Liao Z., Luo Y., Luo Y. (2016). Ocular delivery of cyanidin-3-glycoside in liposomes and its prevention of selenite-induced oxidative stress. Drug Dev. Ind. Pharm..

[308] Huang C., Li C., Muhemaitia P. (2019). Impediment of selenite-induced cataract in rats by combinatorial drug laden liposomal preparation. Libyan J. Med..

[309] Pai R.V., Vavia P.R. (2020). Chitosan oligosaccharide enhances binding of nanostructured lipid carriers to ocular mucins: Effect on ocular disposition. Int. J. Pharm..

[310] Li J., Liu D., Tan G., Zhao Z., Yang X., Pan W. (2016). A comparative study on the efficiency of chitosan-N-acetylcysteine, chitosan oligosaccharides or carboxymethyl chitosan surface modified nanostructured lipid carrier for ophthalmic delivery of curcumin. Carbohydr. Polym..

[311] Liu D., Li J., Pan H., He F., Liu Z., Wu Q., Bai C., Yu S., Yang X. (2016). Potential advantages of a novel chitosan-N-acetylcysteine surface modified nanostructured lipid carrier on the performance of ophthalmic delivery of curcumin. Sci. Rep..

[312] Li J., Tan G., Cheng B., Liu D., Pan W. (2017). Transport mechanism of chitosan-N-acetylcysteine, chitosan oligosaccharides or carboxymethyl chitosan decorated coumarin-6 loaded nanostructured lipid carriers across the rabbit ocular. Eur. J. Pharm. Biopharm..

[313] Liu D., Li J., Cheng B., Wu Q., Pan H. (2017). Ex vivo and in vivo evaluation of the effect of coating a coumarin-6-labeled nanostructured lipid carrier with chitosan-n-acetylcysteine on rabbit ocular distribution. Mol. Pharm..

[314] Salama A.H., Mahmoud A.A., Kamel R. (2016). A novel method for preparing surface-modified fluocinolone acetonide loaded plga nanoparticles for ocular use: In vitro and in vivo evaluations. AAPS PharmSciTech.

[315] Pandit J., Sultana Y., Aqil M. (2017). Chitosan-coated PLGA nanoparticles of bevacizumab as novel drug delivery to target retina: Optimization, characterization, and in vitro toxicity evaluation. Artif. Cells Nanomed. Biotechnol..

[316] Khan N., Ameeduzzafar, Khanna K., Bhatnagar A., Ahmad F.J., Ali A. (2018). Chitosan coated PLGA nanoparticles amplify the ocular hypotensive effect of forskolin: Statistical design, characterization and in vivo studies. Int. J. Biol. Macromol..

[317] Dyawanapelly S., Koli U., Dharamdasani V., Jain R., Dandekar P. (2016). Improved mucoadhesion and cell uptake of chitosan and chitosan oligosaccharide surface-modified polymer nanoparticles for mucosal delivery of proteins. Drug Deliv. Transl. Res..

[318] Mahaling B., Katti D.S. (2016). Physicochemical properties of core-shell type nanoparticles govern their spatiotemporal biodistribution in the eye. Nanomed. Nanotechnol. Biol. Med..

[319] Mahaling B., Katti D.S. (2016). Understanding the influence of surface properties of nanoparticles and penetration enhancers for improving bioavailability in eye tissues in vivo. Int. J. Pharm..

[320] Nasr F.H., Khoee S. (2015). Design, characterization and in vitro evaluation of novel shell crosslinked poly(butylene adipate)-co-N-succinyl chitosan nanogels containing loteprednol etabonate: A new system for therapeutic effect enhancement via controlled drug delivery. Eur. J. Med. Chem..

